# Design, synthesis, and biological evaluation of azole antifungals against multidrug-resistant *Candida auris*

**DOI:** 10.1039/d6md00330c

**Published:** 2026-07-14

**Authors:** Yiyuan Chen, Yunxiao Li, Kazi S. Nahar, Charlotte K. Hind, J. Mark Sutton, Khondaker Miraz Rahman

**Affiliations:** a Institute of Pharmaceutical Science, King's College London 150 Stamford Street London SE1 9NH UK mark.sutton@phe.gov.uk k.miraz.rahman@kcl.ac.uk +44 (0) 1980 612649 +44 (0) 2078 481891; b UK Health Security Agency, Countermeasures Development, Evaluation and Preparedness Manor Farm Road Salisbury SP4 0JG UK mark.sutton@ukhsa.gov.uk; c Department of Natural Sciences, University of Middlesex The Burroughs, Hendon London NW4 4BT UK

## Abstract

Invasive fungal infections cause over one million deaths annually, with the emerging multidrug-resistant pathogen *Candida auris* posing a major therapeutic challenge. Here, we report a series of structurally simplified fluconazole analogues designed to retain antifungal potency through targeted scaffold optimisation. The design strategy involved incorporation of a short heteroaliphatic linker, and replacement of one triazole ring with a substituted phenyl moiety to enable controlled modulation of lipophilicity and target interactions. Eighteen compounds were synthesised with calculated log *P* values of 2.36–3.61 and evaluated against eight *C. auris* isolates from multiple clades, alongside other clinically relevant *Candida* species. Several mono- and di-halogenated analogues showed marked potency gains over fluconazole, with MICs as low as 0.03 μg mL^−1^ against *C. auris* and ≤0.008 μg mL^−1^ against *C. albicans*, representing up to 512-fold improvements. Molecular docking supported enhanced binding to lanosterol 14α-demethylase (LDM) *via* engagement of a hydrophobic sub-pocket. Representative compounds displayed low *in vivo* toxicity in the *Galleria mellonella* model at 20 mg kg^−1^, and compounds 3 and 18 significantly improved survival in *C. auris*-infected larvae at 10 mg kg^−1^, whereas fluconazole showed no significant efficacy at up to 50 mg kg^−1^. These results demonstrate that rational modification of fluconazole scaffold can yield modified azoles with quantitatively improved activity against *C. auris* and other multidrug-resistant *Candida* species.

## Abstract 2

Invasive fungal infections cause over one million deaths annually, and the emerging multidrug-resistant pathogen *Candida auris* presents a major therapeutic challenge.

## Graphical abstract

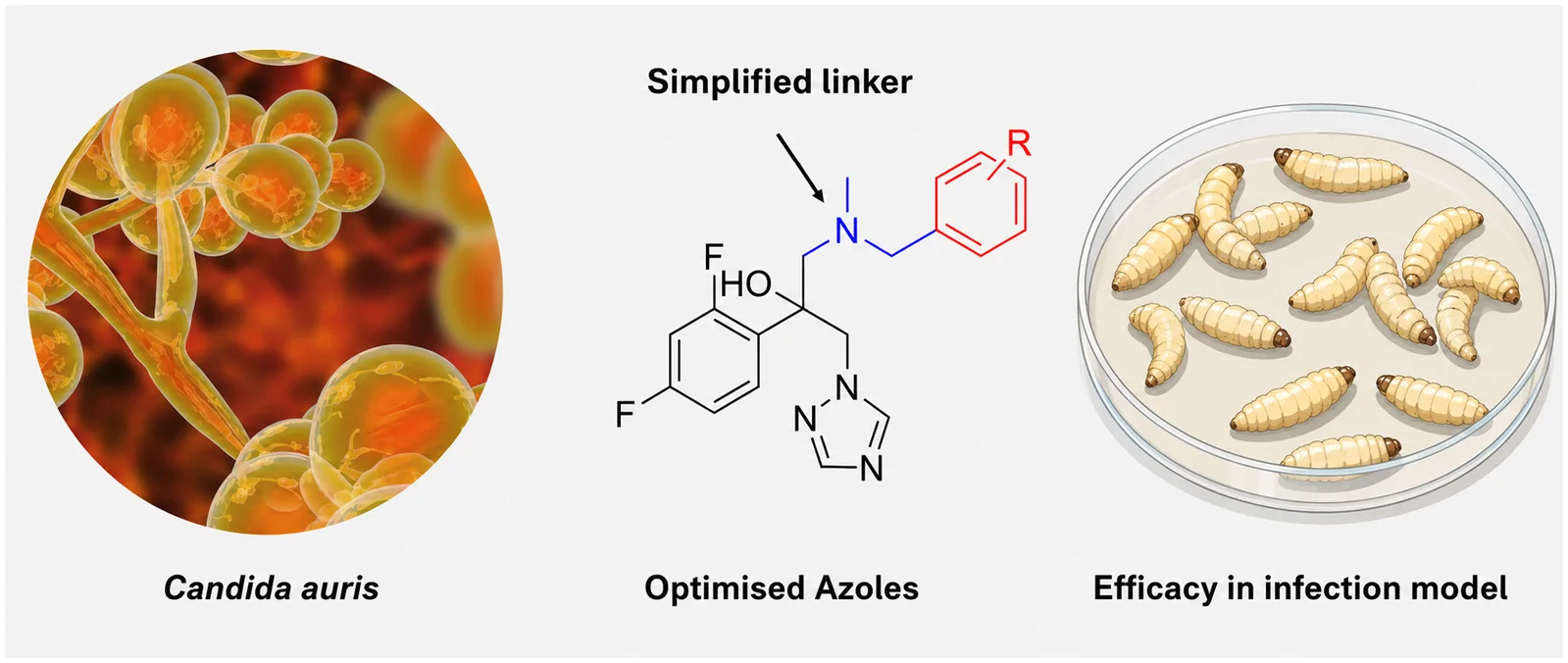

## Introduction

Globally, fungal infections affect over a billion people and contribute to more than one million deaths annually.^1,2^ The use of antifungal drugs is the common treatment for a variety of fungal infections, including candidiasis, aspergillosis, mucormycosis, and cryptococcosis.^3–5^ With the prolonged therapeutic use of antifungals, fungi can develop the ability to evade the effects of drugs designed to kill or inhibit their growth, leading to antifungal resistance, which results in severe and difficult-to-treat infections.^6,7^

Resistant *Candida* species are recognized as one of the main and relatively prevalent fungal pathogens in clinical settings. Data from the CDC indicates that approximately 7% of clinical strains isolated from hospitalized patients with bloodstream infections exhibit resistance to available antifungal drugs.^8^ Among all *Candida* species, *Candidozyma auris* (formerly *Candida auris*) is a rapidly rising multidrug-resistant yeast that can spread quickly and potentially cause acute infectious diseases.^9,10^ Approximately 90% of *C. auris* isolates were resistant to at least one commercially available antifungal drug, while 30% of the clinical strains exhibited resistance to multiple antifungal agents currently on the market.^10^

*C. auris* was first identified in 2006 from the external ear canal of an inpatient in Japan and was subsequently isolated and described in 2009.^11^ Clinically, *C. auris* isolates have been obtained from various specimen sources, including blood (67.5%), urine (6.2%), ear discharge (4.1%), the respiratory system (3.0%), and central venous catheters (2.6%).^12^ The data indicate that bloodstream infections (BSIs) are the most frequently identified invasive infections caused by *C. auris*. Additionally, infections involving *C. auris* are associated with a higher mortality rate, ranging from 33% to 72%, which varies significantly among different geographic areas and settings. Better survival rates have also been observed in paediatric patients.^13^

There are generally only four major types of antifungal drugs available for treating systemic fungal infections in clinical practice: azoles (fluconazole, itraconazole, voriconazole), polyenes (amphotericin B, nystatin), echinocandins (caspofungin, micafungin, anidulafungin), and pyrimidine analogues (mainly flucytosine).^14–18^ Azole antifungals, including imidazoles, triazoles, and tetrazoles, are the most commonly used drug class to disrupt fungal growth by inhibiting the synthesis of ergosterol through targeting the cytochrome P450 enzyme sterol 14α-demethylase.^19–21^

**Fig. 1:**
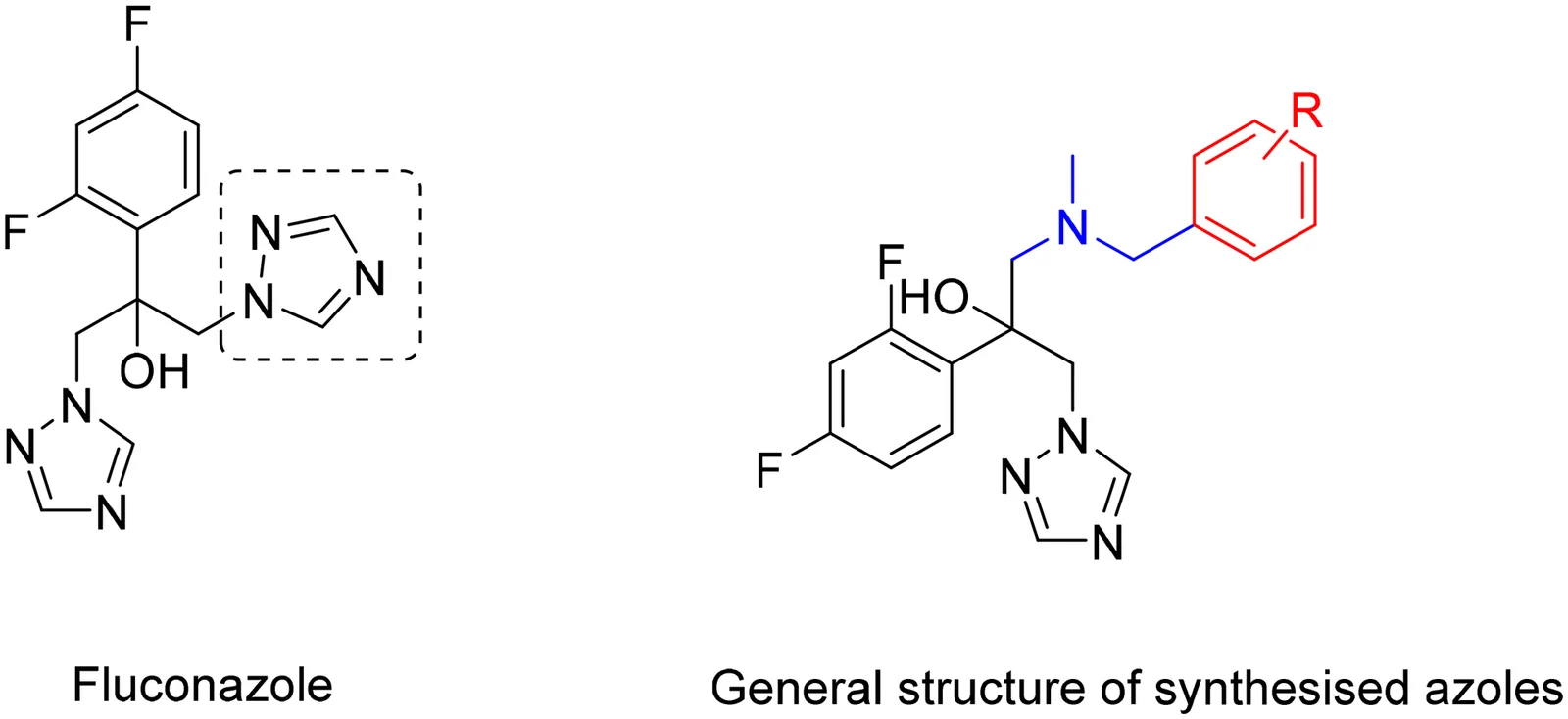
Structure of fluconazole and the general structure of the modified azole compounds.

Despite the widespread use of azole antifungals, their effectiveness has been significantly compromised by the emergence of resistance.^22,23^ A primary strategy of azole-resistant fungal pathogens is to reduce the intracellular concentration of the drug to non-lethal levels by overexpressing efflux pumps.^24,25^ In our recent work, we reported a series of structurally modified azole antifungals designed to overcome azole resistance in *C. auris* by introducing targeted aromatic substitutions to the fluconazole scaffold.^26^ These compounds demonstrated markedly improved *in vitro* potency against multiple *C. auris* isolates, validating the strategy of scaffold modification to restore antifungal activity. However, these first-generation analogues were significantly more hydrophobic than fluconazole and incorporated bulky cyclic amine motifs, features that may negatively impact solubility, distribution, and *in vivo* performance. In the present study, we sought to address these limitations through a second-generation design strategy focused on structural simplification while testing whether antifungal activity against *C. auris* and other multidrug resistant *Candida* species would be retained. The design strategy involved incorporation of a short heteroaliphatic linker, and replacement of one triazole ring of fluconazole with a substituted phenyl moiety to enable controlled modulation of lipophilicity and target interactions (Fig. 1). This approach allowed us to examine whether a less bulky and structurally simplified fluconazole-derived scaffold could retain activity against azole-resistant *Candida* species. Fluconazole was therefore used as the main comparator, as it provides the most relevant benchmark for assessing whether these modifications restore antifungal activity.

## Results and discussion

### Design of modified azole compounds

The modified azoles reported in this study were designed to investigate what types of structural modifications to the fluconazole scaffold could improve antifungal activity and overcome fluconazole resistance in *C. auris* and other clinically relevant *Candida* pathogens. In particular, we sought to determine whether targeted modification of fluconazole could generate compounds with enhanced potency against fluconazole-resistant isolates.

The design strategy focused on systematic alteration of key structural features of fluconazole while preserving elements required for azole-mediated target engagement. One triazole moiety was replaced with a substituted phenyl ring to explore whether modulation of hydrophobicity, electronics, and binding-site interactions could improve antifungal activity. Substituent variation on the phenyl ring enabled controlled exploration of structure–activity relationships and allowed assessment of how different aromatic groups influenced potency against susceptible and resistant *Candida* isolates.

A short heteroaliphatic linker was incorporated to connect the modified aromatic region to the core azole pharmacophore. This design allowed us to examine whether a relatively compact scaffold could maintain productive interactions within the lanosterol 14α-demethylase (LDM) binding site. The linker also provided a means to tune molecular flexibility, polarity, and spatial presentation of the substituted phenyl group without introducing excessive molecular bulk.

Overall, this approach was intended to define which fluconazole-derived structural modifications are compatible with improved antifungal activity, particularly against fluconazole-resistant *C. auris*. The resulting compound series enabled systematic evaluation of how changes in aromatic substitution, linker architecture, and scaffold composition influence activity across *C. auris* clades and other clinically important *Candida* species.

The designed compounds were subsequently used in computational analyses against the target LDM from various *Candida* species, alongside the clinically relevant drug fluconazole (Table 1). All designed compounds showed a higher ChemScore and more negative Δ*G* than fluconazole. This result indicated that they were estimated to have a larger affinity binding to target LDM than unmodified fluconazole (Table 1).

**Table 1:** The comparative binding affinity of novel azole analogues and commercially available azoles with fungal target lanosterol 14α-demethylase (LDM)

Compound	*C. albicans* calLDM		*C. auris* carLDM (A0A2H4QC40)		*C. auris* carLDM (A0A2Z4EHX0)	
	Chem score	Δ*G* (kcal mol^−1^)	Chem score	Δ*G* (kcal mol^−1^)	Chem score	Δ*G* (kcal mol^−1^)
Fluconazole	18.16	−18.94	16.07	−18.8	16.02	−18.51
1	36.53	−37.22	31.45	−32.51	31.78	−32.31
2	35.5	−36.8	30.6	−33.9	31	−34.2
3	35.8	−37.3	31.4	−34.9	31.7	−35.2
4	36.3	−38.2	33	−35.5	33.1	−34.6
5	35.09	−36.19	30.15	−34.36	30.77	−34.83
6	35.4	−36.9	30.8	−34.6	31.2	−34.7
7	36.28	−38.49	33.48	−35.72	32.95	−33.42
8	32	−34	30.5	−33	31.5	−33.5
9	32.23	−34.35	30.78	−33.37	32.77	−34.11
10	31.87	−34.64	30.29	−34.72	29.57	−31.67
11	33	−35.8	31	−33.8	31.5	−33.9
12	34.19	−36.92	32.01	−34.22	30.69	−33.1
13	30.9	−33.77	30.31	−34.67	30.89	−36.15
14	31.8	−35.5	31.3	−36.5	33	−37.2
15	31.76	−35.59	32.39	−38.02	35.37	−38.33
16	33.5	−36	32	−36	33	−36.8
17	34.5	−36.8	32.3	−36.4	33.5	−37.4
18	35.62	−37.35	32	−36.72	30.58	−32.55

### Synthesis of designed compounds

The modified azole series was designed to systematically evaluate the effect of aromatic substitution on antifungal activity through controlled variation of phenyl ring substituents. The initial set of compounds comprised mono-halogenated analogues bearing F, Cl, or Br at either the 3- or 4-position. These substituents were selected to modulate steric bulk, polarizability, and lipophilicity while maintaining a consistent scaffold. A second set of compounds incorporated a hydroxy group on the phenyl ring in either a 1,3- or 1,4-relationship relative to the halogen. This enabled assessment of positional effects on polarity and intramolecular interactions while retaining a halogen substituent for controlled hydrophobic contribution.

To complete the SAR series and isolate the impact of halogen substitution patterns, a final set of di-halogenated analogues was synthesised. These compounds contained a fluorine substituent combined with a second halogen (F, Cl, or Br) in a 1,3-relationship, allowing evaluation of cumulative halogen effects on steric and electronic properties within a fixed substitution geometry.

**Fig. 2:**
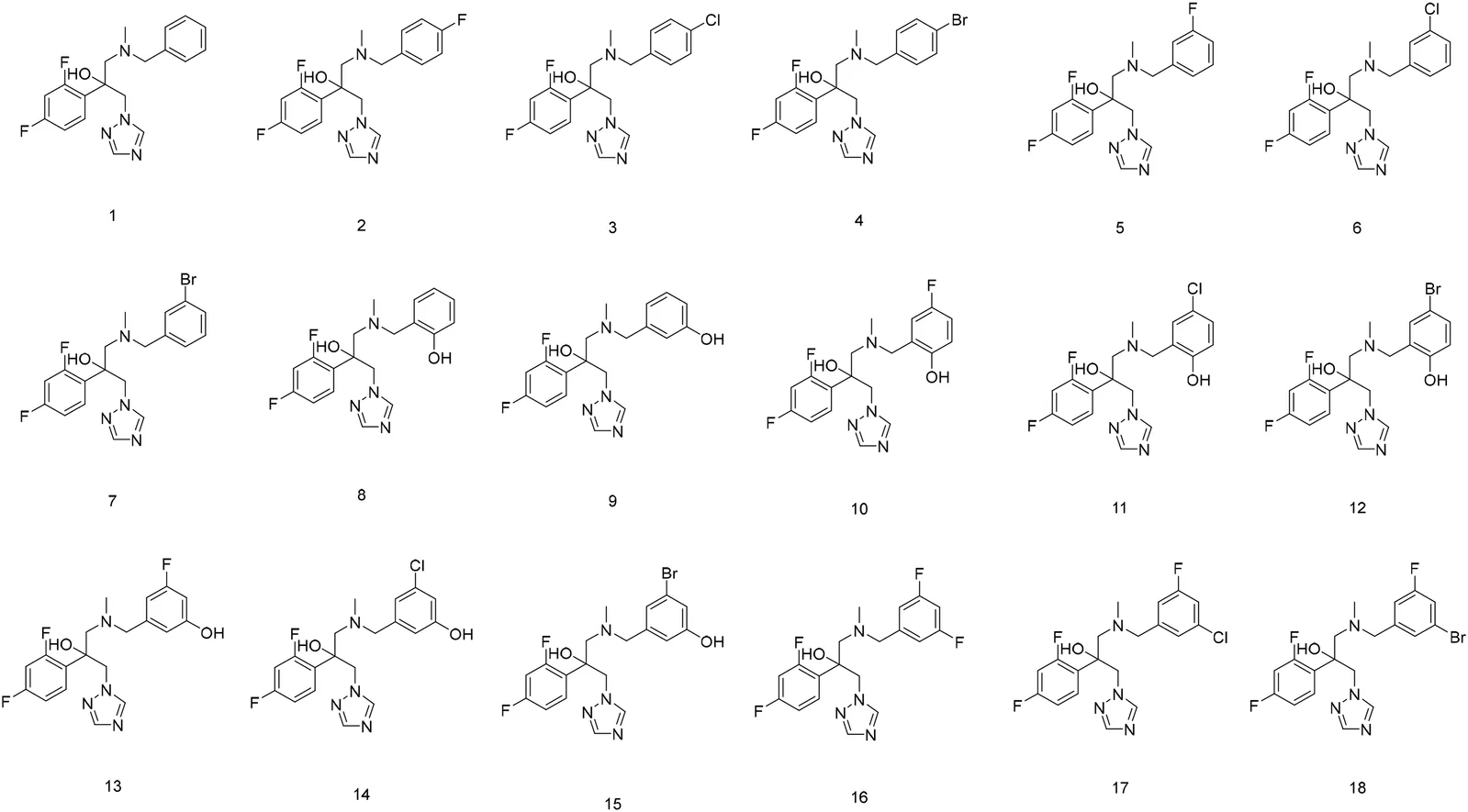
Structures of synthesised azole compounds.

**Scheme 1:**
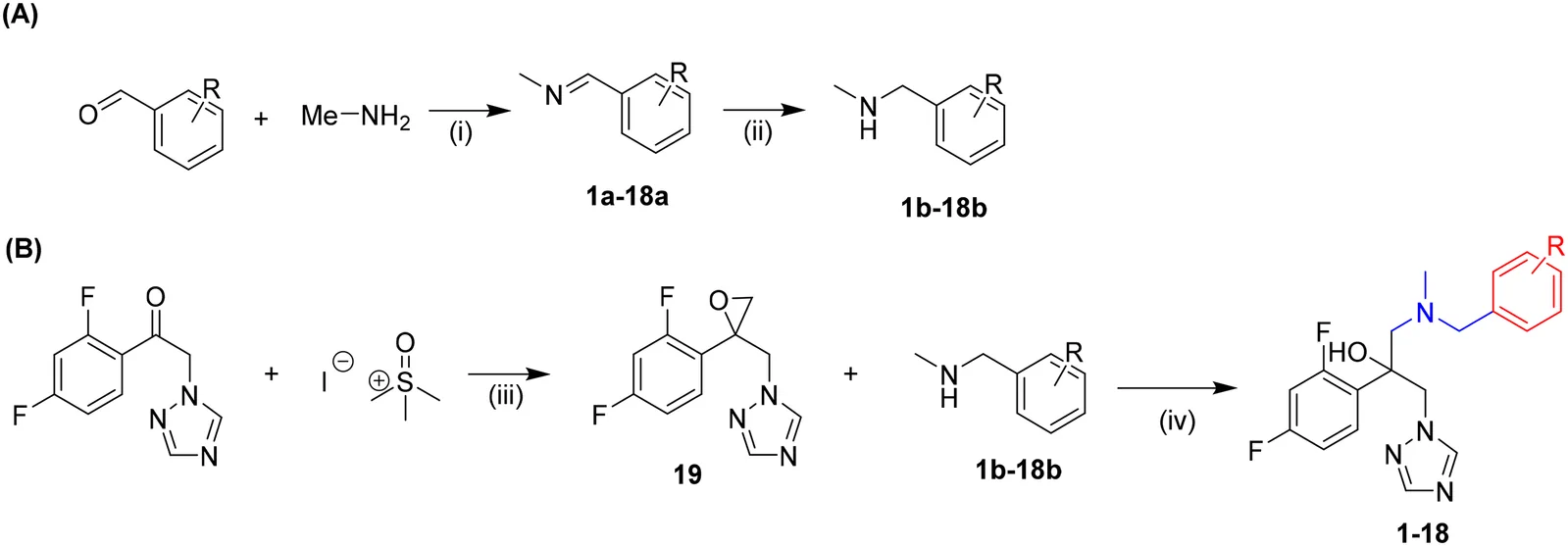
General synthetic routes for designed compounds where different aromatic fragments (A) were connected to the fluconazole core *via* a heteroaliphatic linker (B). Conditions: (i) MeOH, rt; (ii) NaBH(OAc)_3_, MeOH, 0 °C – rt; (iii) NaOH (aq.), toluene, 80 °C, microwave 50 min; (iv) TEA, EtOH, 80 °C, overnight.^28,29^

This library comprised 18 compounds (Fig. 2) prepared through a two-step synthetic seqeuence. In the first step, aldehydes and amines were condensed to form the corresponding imines (1a–18a), which were subsequently reduced to the respective amines (1b–18b) (Table S1), completing the reductive amination process. These amine fragments were then attached to the azole core through an epoxide ring-opening reaction to afford the final compounds (Scheme 1A).

The epoxide core of the azole compounds was prepared using the classic Corey–Chaykovsky reaction conditions^27^ (Scheme 1B). The final compounds were obtained by reacting amine fragments 1b–18b with the epoxide core 19 through a nucleophilic epoxide ring-opening reaction. This process yielded final compounds 1–18, which were subsequently evaluated for their antifungal activity.

### Antifungal activity of compounds

All 18 compounds were evaluated against two antifungal panels. Table 2 presents activity against four WHO priority public health *Candida* and *Candida* like species: *C. albicans*, *Nakaseomyces glabratus*, *C. tropicalis* and *C. parapsilosis*. Table 3 presents activity against an extended *C. auris* panel comprising eight isolates from multiple clades and azole susceptibility profiles.

**Table 2:** Antifungal activity (μg mL^−1^) of modified fluconazole compounds against *Candida* and *Candida*-like species. The MIC is defined as the lowest concentration to inhibit ≥50% growth, as per EUCAST guidelines for azoles

Compounds	Log *P*	*C. albicans* NCPF 3281	*N. glabratus* NCPF 8018	*C. tropicalis* NCPF 8760	*C. parapsilosis* NCPF 3209
Fluconazole	0.88	0.063–0.125	4	16	0.25
1	2.74	≤0.25	≤0.25	1	≤0.25
2	3.13	0.016	0.5	8	0.063
3	3.38	≤0.008	0.063	2	0.016
4	3.40	≤0.008	0.063	2	0.002–0.008
5	3.16	≤0.25	≤0.25	0.5	≤0.25
6	3.29	≤0.008	0.125	2	0.002–0.031
7	3.43	≤0.25	≤0.25	0.25–1	≤0.25
8	2.47	≤0.008	1	16	0.063
9	2.36	≤0.25	1–2	8	≤0.25
10	2.62	≤0.125	≤0.125–2	4–16	≤0.125
11	2.80	≤0.008	0.25	4	0.004–0.031
12	2.88	0.125	0.125	1	0.125
13	2.64	≤0.125	≤0.125–1	4	≤0.125
14	2.88	≤0.008	1	32	0.063
15	2.72	≤0.125	≤0.125	2	≤0.125
16	3.17	≤0.008	0.25	2	0.004–0.063
17	3.61	≤0.008	0.063	4	0.031
18	3.53	≤0.125	≤0.125	0.5–8	≤0.125

**Table 3:** Antifungal activity (μg mL^−1^) of modified fluconazole compounds against *C. auris* isolates. The MIC is defined as the lowest concentration to inhibit ≥50% growth, as per EUCAST guidelines for azoles

Compounds	Log *P*	*C. auris*							
		TDG 1912	TDG 2512	TDG 1102	TDG 2211	TDG 2506	NCPF 8984	NCPF 8971	NCPF8977
		Clade III	Clade I^a^	Clade III	Clade I	Clade II	Clade II	Clade I	Clade III
Fluconazole	0.88	>128	8	64	64	128	128	32	16
1	2.74	16	0.25	4	0.125	8	4	0.5	4
2	3.13	8	0.5	8	0.25	8	16	1	2
3	3.38	2	0.063	2	0.031	4	2	0.25	0.25
4	3.40	2	0.031	4	0.031	4	1	0.25	0.25
5	3.16	8	0.25	4	1	4	8	0.25	1
6	3.29	2	0.25	8	0.125	8	4	1	0.5
7	3.43	4	0.25	2	0.063	4	2	0.25	1
8	2.47	4	0.5	32	0.5	16	16	2	2
9	2.36	16	1	8	1	32	32	2	8
10	2.62	32	0.25	16	0.125	8	8	2	1
11	2.80	4	0.5	16	0.125	8	8	1	0.5
12	2.88	0.5–8	0.063	8	0.063	8	8	2	2
13	2.64	8–32	0.5	4	0.5	16	32	2	2
14	2.88	2	1	8	1	16	32	0.5	1
15	2.72	4	0.25	16	0.5	16	16	0.25	0.5
16	3.17	2	0.5	8	0.25	8	16	1	0.5
17	3.61	1	0.25	8	0.063	16	16	1	0.5
18	3.53	2	1	16	0.5	16	16	0.25	1

a Presumptive clade I.

The *C. auris* panel included isolates from clade I (TDG2211, NCPF8971), presumptive clade I (TDG2512), clade II (TDG2506, NCPF8984), and clade III (TDG1102, NCPF8977, TDG1912), encompassing both azole-susceptible and azole-resistant phenotypes. In the absence of defined EUCAST or CLSI fluconazole breakpoints for *C. auris*, a presumed breakpoint of 32 μg mL^−1^ was applied.

Overall, modification of the fluconazole scaffold resulted in improved antifungal activity across both the *C. auris* panel and the non-*auris Candida* species, with the magnitude of improvement varying by substitution pattern and species. Compound 1 showed a 4- to 512-fold reduction in MIC values relative to fluconazole across most strains tested, including multiple *C. auris* isolates, confirming that targeted scaffold modification could enhance activity in this priority pathogen. In contrast, strains already highly sensitive to fluconazole, such as *C. albicans* NCPF3281 and *C. parapsilosis*, showed smaller relative improvements, consistent with limited headroom for further potency gains.

To assess the effect of aromatic mono-halogenation, compound 1 was used as the reference. Against *C. auris*, mono-fluorinated analogues generally showed MIC values comparable to compound 1, with limited additional benefit. For example, compound 2 (*para*-fluoro) showed modest improvements against *C. albicans* and *C. parapsilosis* but little or no improvement against several *C. auris* isolates, including NCPF8984. Compound 5 (*meta*-fluoro) showed a four-fold improvement against *C. auris* NCPF8977 but reduced or unchanged activity in other *C. auris* strains. These trends indicate that fluorine substitution alone does not consistently enhance activity, particularly against *C. auris*.

In contrast, mono-chlorinated (compounds 3 and 6) and mono-brominated (compounds 4 and 7) analogues showed more consistent improvements across both *C. auris* and the non-*auris Candida* panel. *Para*-substituted chloro- and bromo-analogues generally outperformed their *meta*-substituted counterparts, with improved MIC values observed across multiple *C. auris* isolates as well as *C. albicans* and *C. parapsilosis*. Changes in halogen identity (Cl *versus* Br) at the same position resulted in relatively small differences in activity, suggesting that steric bulk and polarizability were tolerated across species. Fluorinated analogues were generally less active by comparison.

In line with the design rationale, a second subset of compounds incorporated a hydroxyl group on the phenyl ring. Across the *C. auris* panel, hydroxylation led to modest or neutral effects on activity relative to the corresponding mono-halogenated analogues, with MIC values typically within two-fold. Positional variation (1,3- *versus* 1,4-relationship between hydroxyl and halogen) had little effect on *C. auris* activity. In the supporting *Candida* species, particularly *C. parapsilosis* and *C. tropicalis*, hydroxylated analogues often showed MIC values comparable to fluconazole, indicating that increased polarity did not uniformly enhance activity across species.

To complete the SAR series and decouple polarity effects from halogen substitution, a final set of di-halogenated analogues was synthesised, replacing the hydroxyl group with a second halogen in a 1,3-relationship. Relative to the hydroxy-halogenated analogues, the di-halogenated compounds displayed improved antifungal activity, particularly in fluorinated and chlorinated derivatives. This trend was most pronounced against *C. albicans* and *C. parapsilosis*, and less evident in brominated analogues and across the *C. auris* panel.

Compounds 16 (F/F) and 17 (F/Cl) showed the most potent activity within this set, with MIC values of ≤0.008 μg mL^−1^ against *C. albicans* and 0.004–0.063 μg mL^−1^ against *C. parapsilosis*. These values represent a substantial improvement compared with compound 13, which contains a single halogen and a hydroxyl group at the same position and exhibited MIC values of ≤0.125 μg mL^−1^ against both species. Across the series, introduction of a second halogen generally enhanced activity against *C. albicans*, *N. glabratus*, and *C. parapsilosis* relative to mono-halogenated analogues. Against *C. auris*, these compounds also showed improved and more consistent activity relative to mono-halogenated and hydroxy-halogenated analogues, although the magnitude of improvement was generally smaller.

Comparison of di-halogenated compounds further indicated that fluorine–fluorine (16) and fluorine–chlorine (17) combinations were modestly more potent than the fluorine–bromine analogue (18) against *C. albicans* and *C. parapsilosis*. Compound 18 showed activity comparable to the fluorine–hydroxyl analogue 13, with MIC values of ≤0.125 μg mL^−1^ against both species. Relative to compounds 16 and 17, MIC values for compound 18 increased approximately 15-fold in *C. albicans* and up to 30-fold in *C. parapsilosis*. Across the series, the introduction of a second halogen generally enhanced activity relative to mono-halogenated analogues, while avoiding the polarity increase associated with hydroxyl substitution.

### Investigation of stereoselective synthesis of compound libraries using chiral HPLC

As part of the second-generation design strategy, smaller amine nucleophiles were used for epoxide ring opening compared to the large nucleophiles used for the previously reported azole modifications.^26^ This structural simplification raised the possibility that epoxide opening could proceed with reduced stereochemical control, potentially leading to racemic mixtures.^30^ It was therefore important to establish whether shortening the amine fragment compromised the stereochemical outcome of the synthetic route.

To address this, chiral high-performance liquid chromatography (HPLC) analysis was performed on two representative compounds: compound 3 (a mono-substituted analogue) and compound 18 (a di-substituted analogue). These compounds were selected to reflect both early and late stages of the SAR series and to assess whether increased substitution on the aromatic ring influenced stereochemical control. Chiral HPLC analysis was conducted using a Daicel CHIRALPAK AD-H column under multiple isocratic MeCN/H_2_O conditions (30–70%) containing 0.1% formic acid. The resolving capability of the method was validated using a racemic reference mixture, which showed separation of enantiomeric peaks under the reported conditions (Fig. S19).

**Fig. 3:**
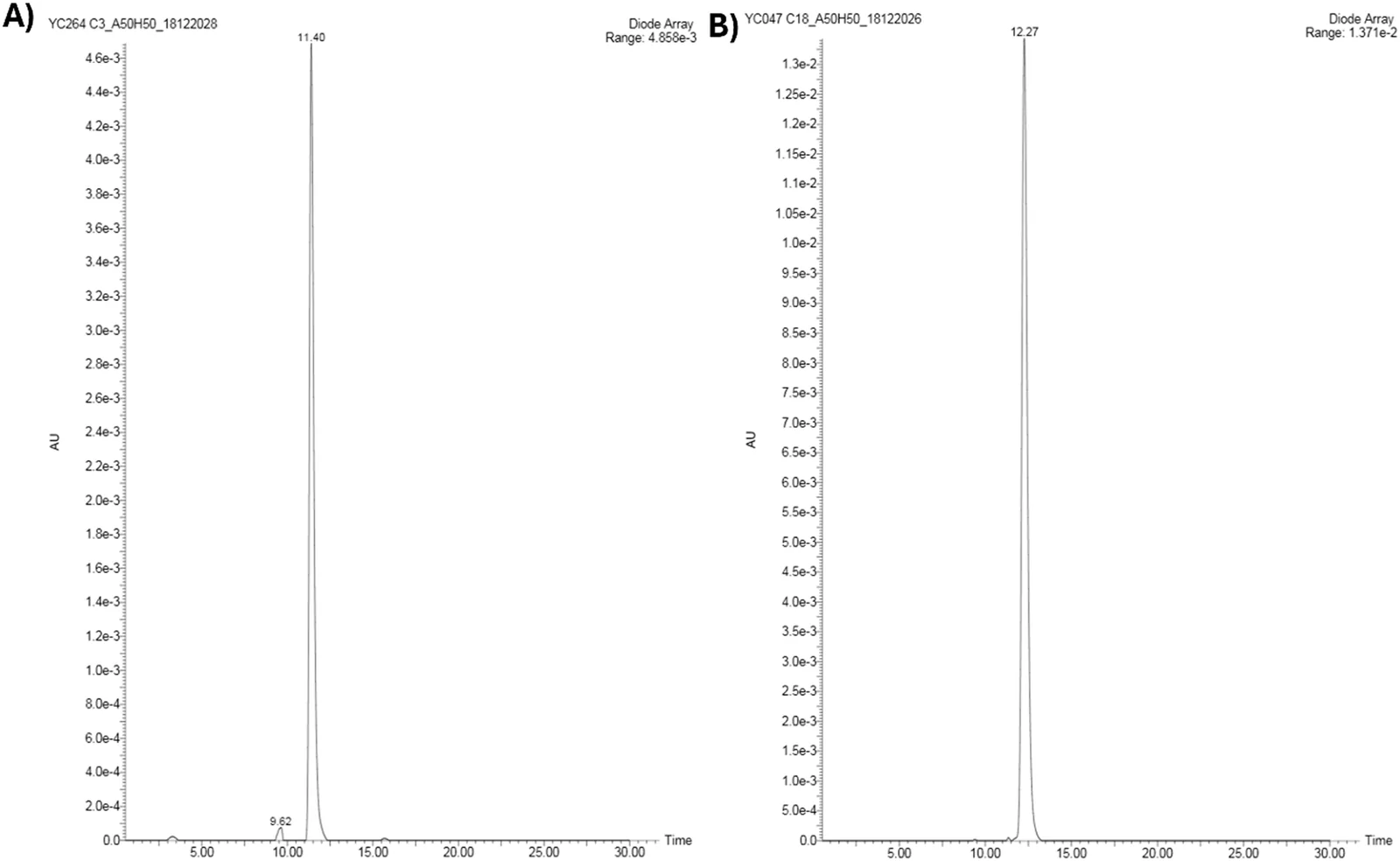
Reverse phase chiral HPLC analysis of A) compound 3 showing a single peak at RT 11.40 minutes and B) compound 18 showing a single peak at RT 12.27 minutes. HPLC method: MeCN/H_2_O/formic acid (50/50/0.1%), isocratic run for 30 minutes, flow rate 0.5 mL min^−1^, monitored at *λ* = 260 nm.

For compound 3, chiral HPLC analysis consistently revealed a single peak across all solvent systems tested (Fig. 3A and S20), with no evidence of additional enantiomeric or diastereomeric species. Similarly, compound 18 (Fig. 3B and S21) displayed a single, well-resolved peak under the conditions examined. Together with the racemic reference validation, these data support that compounds 3 and 18 were obtained as single detectable enantiomeric species rather than racemic mixtures.

These results demonstrate that, despite deliberate shortening of the amine fragment for these modified azole compounds, the nucleophilic epoxide ring-opening reaction remains highly stereoselective. This suggests that the remaining steric bulk and conformational constraints of the amine nucleophiles are sufficient to direct the attack in a stereochemically controlled manner. The findings are consistent with previous reports showing that epoxide ring opening with hindered or conformationally restricted amines can proceed with strong stereochemical preference and confirm that the revised synthetic strategy does not compromise stereochemical integrity.

### Mechanism of action study of modified azole compounds

#### *In silico* study with lanosterol 14α-demethylase (LDM) enzyme in *C. auris*

**Fig. 4:**
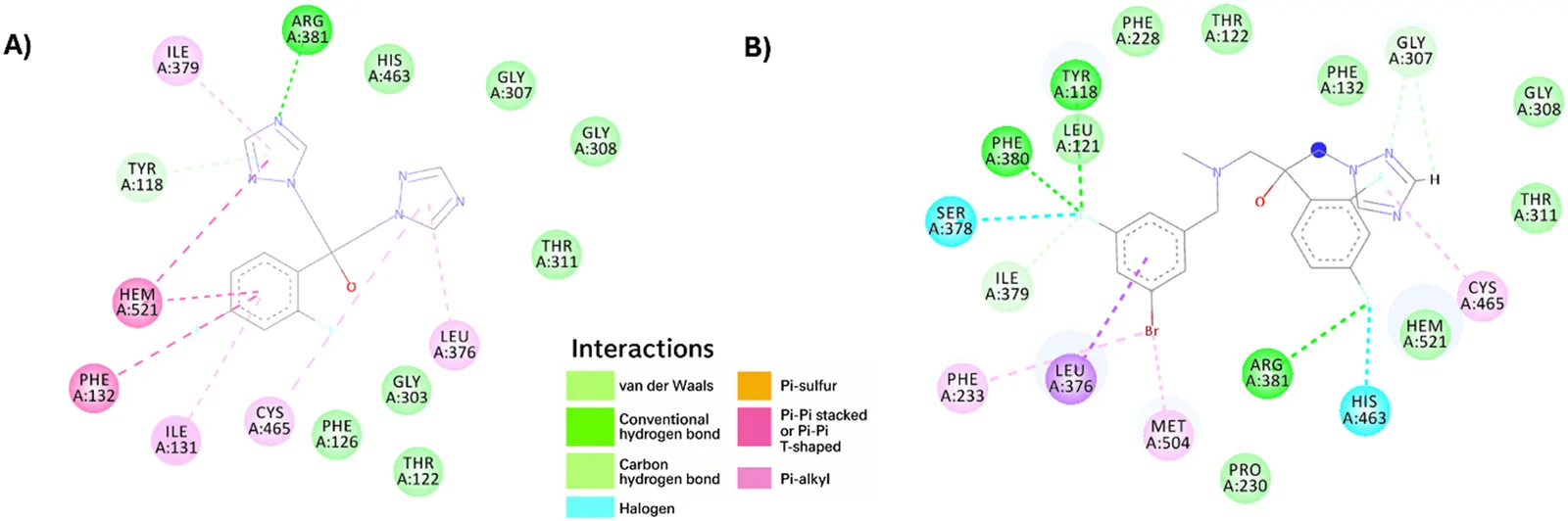
Differences in 2D binding interactions observed for fluconazole (A), and compound 18 (B) with lanosterol 14α-demethylase (LDM) enzyme in *C. auris*.

To further explore the differences in activity observed between fluconazole and the modified compound 18, particularly in *C. auris* strains, we evaluated the binding of these two compounds to the lanosterol 14α-demethylase (LDM) enzyme in *C. auris* using molecular docking. As illustrated by the 2D and 3D interaction maps (Fig. 4 and S22), fluconazole and compound 18 occupy overlapping but distinct regions within the LDM active site. Fluconazole primarily engages a more hydrophilic region of the binding cavity, with interactions dominated by polar and π–π contacts involving the triazole rings and surrounding residues. In contrast, compound 18 adopts a binding pose that extends further into a predominantly hydrophobic sub-pocket of the enzyme. This orientation enables compound 18 to engage a broader set of residues through hydrophobic, π-alkyl, and π–π interactions.

The di-halogenated aromatic ring of compound 18 appears to play a key role in stabilising this binding mode by promoting favourable interactions with hydrophobic residues lining the pocket, consistent with the design rationale aimed at modulating aromatic substitution patterns. The more extended interaction network observed for compound 18, relative to fluconazole, is also reflected in the higher ChemScore values and more favourable docking scoring-function outputs (Table 1), supporting a more favourable predicted binding pose within the LDM active site.

Importantly, these modelling results align with the experimental SAR, where di-halogenated analogues showed improved and more consistent activity against *C. auris* compared with mono-substituted and hydroxy-substituted compounds. While docking scores alone do not directly translate to biological potency, the observed shift towards a more hydrophobic binding environment and increased residue engagement provides a plausible structural basis for the enhanced activity of compound 18 against *C. auris*. This observation is in line with the results obtained for the first generation modified azole compounds reported by us.

### Toxicity Evaluation in the *Galleria mellonella* Model

To assess *in vivo* tolerability of representative compounds from the series, toxicity studies were conducted in the *Galleria mellonella* larval model using one mono-halogenated analogue (compound 3) and one di-halogenated analogue (compound 18), administered at a dose of 20 mg kg^−1^ (Table 4). These compounds were selected to reflect early and late stages of the aromatic substitution SAR.

**Table 4:** Toxicity tests in *G. mellonella* model for two selected compounds at 20 mg kg^−1^

	24 h	48 h	72 h	96 h	120 h	Survival rate
PBS	10	10	10	10	10	
3	10	8	8	8	7	70%
18	10	10	10	10	10	100%

Across the 120 hour observation period, both compounds demonstrated low toxicity relative to the phosphate-buffered saline (PBS) control. Compound 3 showed a modest reduction in larval survival over time, with a final survival rate of 70% at 120 hours, indicating limited toxicity at the tested dose. In contrast, compound 18 exhibited no observable toxicity, with 100% larval survival maintained throughout the entire study duration.

The favourable tolerability profile of compound 18 is notable given its enhanced antifungal activity against *C. auris* and supports the design strategy of controlled di-halogenation without introducing additional toxicity liabilities. Importantly, these results suggest that increasing aromatic substitution to improve antifungal activity does not necessarily compromise *in vivo* tolerability within this scaffold.

### Efficacy study in *Galleria mellonella* model

**Fig. 5:**
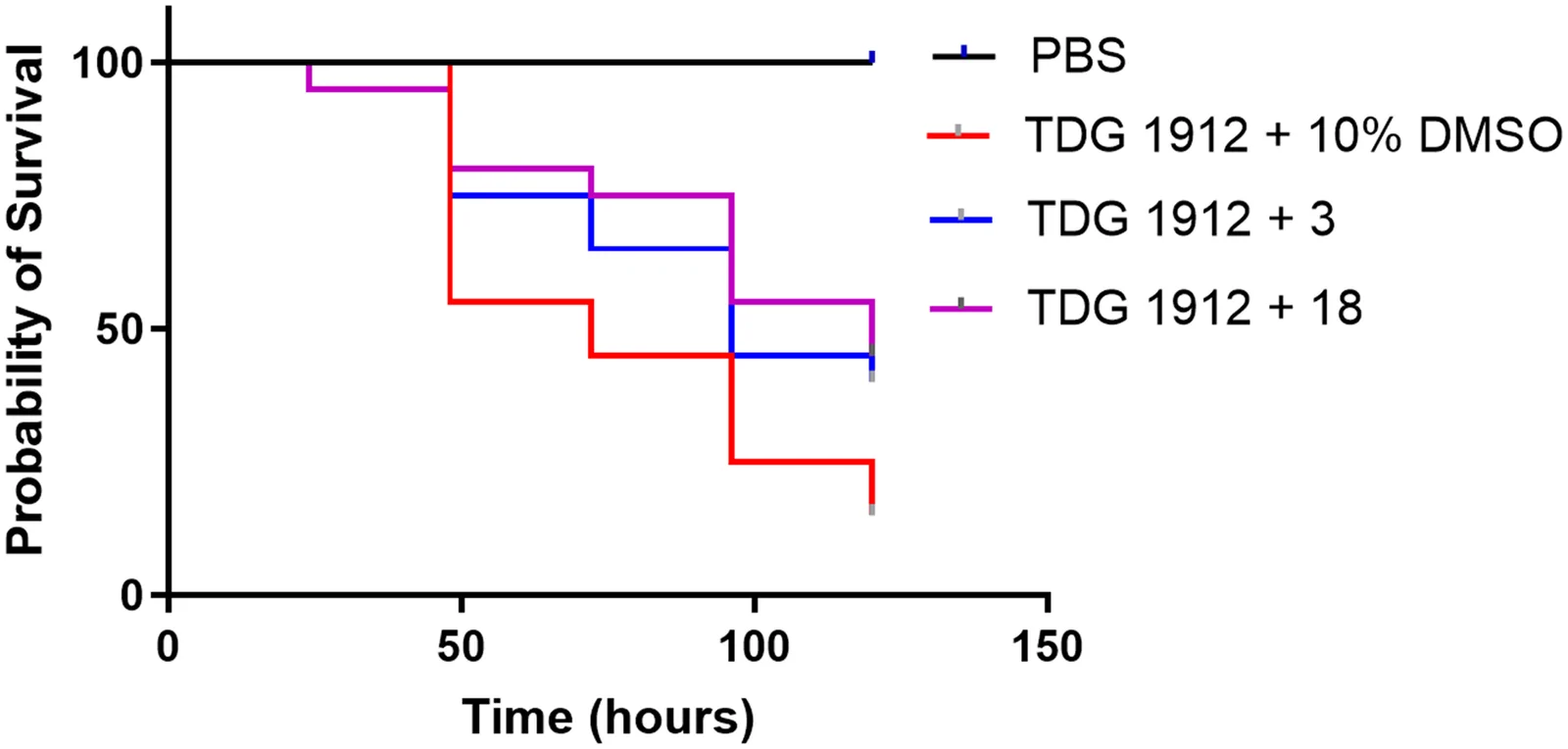
Efficacy of modified fluconazole compounds 3 and 18 against *C. auris* TDG1912 infection in *G. mellonella* model.

Compounds 3 and 18 were selected as representative examples to evaluate the *in vivo* efficacy of the modified azole series in a *G. mellonella* infection model^31^ infected with *C. auris* (TDG1912). In the *G. mellonella* model, compound 3 demonstrated significant antifungal efficacy at a dose of 10 mg kg^−1^, resulting in a clear survival benefit compared with infected controls (Fig. 5, *P* = 0.002). Similarly, compound 18 showed significant *in vivo* efficacy, with a substantial proportion of larvae surviving to the end of the experiment and a highly significant improvement in survival relative to controls (*P* = 0.0001). Fluconazole was used as a comparator and no significant efficacy observed at 10, 20 or 50 mg kg^−1^ (Fig. S23). These preliminary *in vivo* screening data demonstrate that rationally simplified, more drug-like azole analogues retain *in vivo* antifungal activity in *G. mellonella* infected with *C. auris*.

## Conclusion

In this study, we report the design, synthesis, and evaluation of modified azole antifungals developed to identify fluconazole scaffold modifications that can improve activity against fluconazole-resistant *C. auris* and other clinically relevant *Candida* pathogens. The series was constructed through systematic variation of aromatic substitution patterns, including mono-halogenated, hydroxy-halogenated, and di-halogenated analogues, enabling clear structure–activity relationship analysis within a synthetically accessible scaffold. The modified azoles showed improved activity relative to fluconazole against multiple *Candida* species, including *C. albicans*, *N. glabratus*, *C. tropicalis*, *C. parapsilosis*, and *C. auris*. Importantly, replacement of one triazole ring of fluconazole with a substituted phenyl ring improved activity against fluconazole-resistant isolates in *C. auris* and other multidrug-resistant *Candida* species. This provides an important medicinal chemistry design principle for developing potent azole compounds with improved activity against these resistant pathogenic fungi. Molecular modelling against *C. auris* lanosterol 14α-demethylase (LDM) revealed binding modes distinct from fluconazole, with di-halogenated compounds engaging a hydrophobic sub-pocket and showing stronger predicted binding. *In vivo* evaluation in the *Galleria mellonella* model demonstrated low toxicity, particularly for the di-halogenated lead compound. Collectively, these findings establish substituted phenyl replacement of one triazole ring as a promising strategy for improving activity against fluconazole-resistant isolates and provide a platform for further optimisation.

## Experimental section

### Chemistry

#### General chemistry

^1^H NMR and ^13^C NMR spectra were recorded on Bruker Spectrospin Spectrometer (400 MHz, 25 degree) equipped with SampleXpress autosampler system (Bruker). The NMR spectrometer uses the solvent stated as the internal reference and trimethylsilane (TMS) as the external reference (0.00 ppm). Chemical shifts δH and δC are recorded in parts per million (ppm) and referenced to the residual solvent peak. Coupling constants (*J*) are recorded to the nearest 0.01 Hz. Multiplicities are reported as broad (br), singlet (s), doublet (d), doublet of doublets (dd), double doublets of doublets (ddd), doublet of triplets (dt), triplet (t), triplet of doublets (td), quartet (q), quintet (quin) or multiplets (m). Assignment of ^1^H and ^13^C NMR spectra was made using the aid of TopSpin 3.5 software from Bruker, ACD/Labs or MestReNova from Mestrelab Research. Purity is calculated using the HPLC-LCMS peak areas.

LC-MS system used is a Waters Alliance 2695 system with an elution in gradient. Low resolution mass spectra were analyzed and recorded on a Waters QZ instrument using electrospray ionization (ESI) and is coupled to a high-performance liquid chromatography (HPLC) system. Selected mass-to-charge ratio peaks (*m*/*z*) are quoted in Daltons. HPLC grade solvents were used for mobile phase and a Phenomenex Monolithic C18 50 × 4.60 mm column was used for stationary phase. UV detection was performed on a Waters 2996 photodiode array detector.

Thin layer chromatography (TLC) was performed using Merck aluminium foil backed sheets precoated with 0.2 mm Kieselgel 60 F254. Product spots were visualized by UV irradiation (*λ*_max_ = 254 nm or 365 nm). Manual flash column chromatography was carried out using Aldrich silica gel 60 Å, 40–63 μm (230–400 mesh). Eluting solvents and retention factors (*R*_f_) were as indicated in the text. Solvents were evaporated under vacuum with a RC-600 evaporating system equipped with a SC-920 G vacuum pump from KNF. The final compounds were dried using a Schlenk line under high-pressure vacuum.

The initial optical rotation of compounds was measured with single wavelength polarimeter ADP440 by Bellingham & Stanley at *l* = 589 nm. Further optical rotatory dispersion (ORD) measurements of selected compounds were measured with Chirascan spectrometers by Applied Photophysics, and were analysed with Pro-Data Chirascan V. 4.5.1848.0. The temperature control model used was Quantum TC125.

All reactions were carried out in oven-dried glassware and all reagents were obtained commercially either from Aldrich Chemicals Ltd., Alfa Aesar Ltd, Fluorochem, Fisher Scientific or Apollo Chemicals Ltd and used as supplied.

### Chemical synthesis

#### General procedure for fluconazole core epoxide synthesis

##### 1-((2-(2,4-Difluorophenyl)oxiran-2-yl)methyl)-1*H*-1,2,4-triazole (19)

Trimethyl sulphoxonium iodide (2 equiv.) was added to toluene (0.12 mmol mL^−1^) containing 1-(2,4-difluorophenyl)-2-(1*H*-1,2,4-triazol-1-yl)ethan-1-one (1 equiv.) and sodium hydroxide 30% (w/w) aqueous solution (10 equiv.). The mixture was heated under microwave (MW) radiation for 50 minutes at 80 °C. Then the mixture was diluted with water and extracted with ethyl acetate. The organic layer was combined and washed with saturated brine, dried over anhydrous magnesium sulphate, and concentrated *in vacuo*.

Pale yellow solid (52%); ^1^H NMR (400 MHz, 25 degree, CDCl_3_) *σ*: 2.83 (1H, d, *J* = 5 Hz), 2.90 (1H, d, *J* = 5 Hz), 4.47 (1H, d, *J* = 15 Hz), 4.79 (1H, d, *J* = 15 Hz), 6.73–6.82 (2H, m), 7.11–7.16 (1H, m), 7.81 (1H, s), 8.03 (1H, s); ^13^C NMR (101 MHz, 25 degree, CDCl_3_) *σ*: 52.1, 53.4 (d, *J* = 3 Hz), 56.1 (d, *J* = 1 Hz), 104.0 (dd, *J* = 26 Hz, *J* = 25 Hz), 111.6 (dd, *J* = 21 Hz, *J* = 3 Hz), 119.4 (dd, *J* = 15 Hz, 4 Hz), 129.5 (dd, *J* = 15 Hz, *J* = 10 Hz, 5 Hz), 144.0, 151.7, 160.5 (dd, *J* = 249 Hz, 12 Hz), 163.0 (dd, *J* = 251 Hz, 12 Hz); *m*/*z*: (ESI^+^) 238.1 ([M + H]^+^); *R*_f_: 0.24 (1 : 1 ethyl acetate : hexane); purity: 95%.

#### General procedure for intermediate synthesis

Anhydrous methylamine (8.0 equiv.) 2.0 M solution in MeOH was added to the benzaldehyde (1.0 equiv.) in ice bath. The mixture was stirred until all starting material was consumed. After the solvent was evaporated, the residue was re-dissolved in MeOH (0.25 mmol mL^−1^), and NaBH(OAc)_3_ (1.0 equiv.) was added in ice bath and the mixture was stirred overnight. After concentrated under reduced pressure, the mixture was diluted with 1 N NaOH and extracted with Et_2_O, after dried with MgSO_4,_ the organic layer was concentrated *in vacuo*. The crude product was purified with gradient *flash* column chromatography eluting with 0–10% MeOH in chloroform to give the pure intermediate compounds.

##### *N*-Methyl-1-phenylmethanamine (1b)

Pale white liquid (87%); ^1^H NMR (400 MHz, 25 degree, MeOH-*d*_4_) *σ*: 2.41 (3H, s), 3.76 (2H, s), 7.26–7.32 (1H, m), 7.35 (4H, d, *J* = 5 Hz); ^13^C NMR (101 MHz, 25 degree, MeOH-*d*_4_) *σ*: 35.1, 55.9, 128.6, 129.6, 129.8, 139.0; *R*_f_: 0.06 (8 : 2 ethyl acetate : MeOH); purity: 96%.

##### 1-(4-Fluorophenyl)-*N*-methylmethanamine (2b)

Pale white liquid (65%); ^1^H NMR (400 MHz, 25 degree, CDCl_3_) *σ*: 2.44 (3H, s), 3.31 (1H, s, br), 3.77 (2H, s), 7.02 (2H, dddd, *J* = 9 Hz, 9 Hz, 4 Hz, 2 Hz), 7.33 (2H, dddd, *J* = 9 Hz, 5 Hz, 3 Hz, 2 Hz); ^13^C NMR (101 MHz, 25 degree, CDCl_3_) *σ*: 35.0, 54.6, 115.5 (d, *J* = 21 Hz), 129.5 (d, *J* = 9 Hz), 130.4 (d, *J* = 8 Hz), 162.4 (d, *J* = 246 Hz); *R*_f_: 0.3 (9 : 1 DCM : MeOH); purity: 96%.

##### 1-(4-Chlorophenyl)-*N*-methylmethanamine (3b)

Pale white liquid (87%); ^1^H NMR (400 MHz, 25 degree, MeOH-*d*_4_) *σ*: 2.35 (3H, s), 3.66 (2H, s), 7.31 (4H, d, *J* = 1 Hz); ^13^C NMR (101 MHz, 25 degree, MeOH-*d*_4_) *σ*: 35.5, 55.5, 129.5, 131.1, 134.0, 139.2; *m*/*z*: (ESI^+^) 157.1 ([M + H]^+^); *R*_f_: 0.2 (8 : 2 ethyl acetate : MeOH); purity: 99%.

##### 1-(3-Fluorophenyl)-*N*-methylmethanamine (5b)

Pale white liquid (75%); ^1^H NMR (400 MHz, 25 degree, MeOH-*d*_4_) *σ*: 2.47 (3H, s), 3.85 (2H, s), 7.05 (1H, td, *J* = 8 Hz, 2 Hz), 7.15 (1H, dt, *J* = 10 Hz, 2 Hz), 7.19 (1H, d, *J* = 8 Hz), 7.38 (1H, td, *J* = 8 Hz, 6 Hz); ^13^C NMR (101 MHz, 25 degree, MeOH-*d*_4_) *σ*: 34.8, 54.9, 115.6 (d, *J* = 21 Hz), 116.6 (d, *J* = 22 Hz), 125.7 (d, *J* = 3 Hz), 131.5 (d, *J* = 8 Hz), 164.4 (d, *J* = 245 Hz), 173.8; *R*_f_: 0.2 (8 : 2 ethyl acetate : MeOH); purity: 96%.

##### 1-(3-Chlorophenyl)-*N*-methylmethanamine (6b)

Pale white liquid (82%); ^1^H NMR (400 MHz, 25 degree, CDCl_3_) *σ*: 2.46 (3H, s), 2.63 (1H, s), 3.77 (2H, s), 7.22–7.26 (3H, m), 7.36 (1H, m); ^13^C NMR (101 MHz, 25 degree, CDCl_3_) *σ*: 35.7, 55.2, 126.6, 127.5, 128.5, 129.8, 134.4, 141.5; *m*/*z*: (ESI^+^) 156.1 ([M + H]^+^); *R*_f_: 0.2 (9 : 1 ethyl acetate : MeOH); purity: 98%.

##### 1-(3-Bromophenyl)-*N*-methylmethanamine (7b)

Pale white liquid (69%); ^1^H NMR (400 MHz, 25 degree, MeOH-*d*_4_) *σ*: 2.36 (3H, s), 3.69 (2H, s), 7.23–7.31 (2H, m), 7.42 (1H, dt, *J* = 8 Hz, 2 Hz), 7.53 (1H, t, *J* = 2 Hz); ^13^C NMR (101 MHz, 25 degree, MeOH-*d*_4_) *σ*: 35.4, 55.6, 123.4, 128.4, 131.3, 131.3, 132.5, 142.9; *m*/*z*: (ESI^+^) 200.0 ([M + H]^+^); *R*_f_: 0.2 (8 : 2 ethyl acetate : MeOH); purity: 99%.

##### 2-((Methylamino)methyl)phenol (8b)

Transparent solid (87%); ^1^H NMR (400 MHz, 25 degree, MeOH-*d*_4_) *σ*: 2.23 (3H, s), 3.67 (2H, s), 6.56–6.61 (2H, m), 6.91–6.97 (2H, m); ^13^C NMR (101 MHz, 25 degree, MeOH-*d*_4_) *σ*: 34.8, 53.2, 116.7, 119.8, 124.5, 129.7, 130.6, 158.9; *R*_f_: 0.1 (8 : 2 ethyl acetate : MeOH); purity: 93%.

##### 3-((Methylamino)methyl)phenol (9b)

Transparent liquid (87%); ^1^H NMR (400 MHz, 25 degree, MeOH-*d*_4_) *σ*: 2.38 (3H, s), 3.66 (2H, s), 6.70 (1H, ddd, *J* = 8 Hz, 2 Hz, 1 Hz), 6.76–6.77 (1H, m), 6.79 (1H, d, *J* = 8 Hz), 7.14 (1H, t, *J* = 8 Hz); ^13^C NMR (101 MHz, 25 degree, MeOH-*d*_4_) *σ*: 35.2, 56.0, 115.4, 116.5, 120.6, 130.6, 140.9, 158.8; *R*_f_: 0.06 *R*_f_: 0.2 (8 : 2 ethyl acetate : MeOH); purity: 94%.

##### 4-Chloro-2-((methylamino)methyl)phenol (11b)

Transparent liquid (78%); ^1^H NMR (400 MHz, 25 degree, CDCl_3_) *σ*: 2.48 (3H, s), 3.12 (1H, s), 3.94 (2H, s), 6.61 (1H, s, br), 6.80 (1H, d, *J* = 9 Hz), 7.00 (1H, d, *J* = 3 Hz), 7.11 (1H, dd, *J* = 9 Hz, 3 Hz); ^13^C NMR (101 MHz, 25 degree, CDCl_3_) *σ*: 34.7, 53.3, 117.8, 122.6, 123.7, 128.8, 129.1, 156.7; *m*/*z*: (ESI^+^) 172.1 ([M + H]^+^); *R*_f_: 0.2 (9 : 1 ethyl acetate : MeOH); purity: 95%.

##### 4-Bromo-2-((methylamino)methyl)phenol (12b)

Pale white liquid (85%); ^1^H NMR (400 MHz, 25 degree, MeOH-*d*_4_) *σ*: 2.46 (3H, s), 3.87 (2H, s), 6.69 (1H, d, *J* = 9 Hz), 7.23 (1H, dd, *J* = 9 Hz, 2 Hz), 7.27 (1H, d, *J* = 2 Hz); ^13^C NMR (101 MHz, 25 degree, MeOH-*d*_4_*I*) *σ*: 34.4, 52.0, 11.9, 118.7, 125.8, 132.9, 133.4, 158.7; *m*/*z*: (ESI^+^) 216.0 ([M + H]^+^); *R*_f_: 0.1 (9 : 1 ethyl acetate : MeOH); purity: 97%.

##### 3-Fluoro-5-((methylamino)methyl)phenol (13b)

White solid (86%); ^1^H NMR (400 MHz, 25 degree, MeOH-*d*_4_) *σ*: 2.40 (3H, s), 3.66 (2H, s), 6.42 (1H, dt, *J* = 11 Hz, 2 Hz), 6.54 (1H, d, *J* = 9 Hz), 6.58 (1H, s); ^13^C NMR (101 MHz, 25 degree, MeOH-*d*_4_) *σ*: 35.0, 55.5, 102.8 (d, *J* = 24 Hz), 106.8 (d, *J* = 22 Hz), 112.7 (d, *J* = 3 Hz), 142.3 (d, *J* = 9 Hz), 160.8 (d, *J* = 12 Hz), 165.2 (d, *J* = 243 Hz); *m*/*z*: (ESI^+^) 156.1 ([M + H]^+^); *R*_f_: 0.07 (9 : 1 ethyl acetate : MeOH); purity: 94%.

##### 3-Chloro-5-((methylamino)methyl)phenol (14b)

White solid (80%); ^1^H NMR (400 MHz, 25 degree, MeOH-*d*_4_) *σ*: 2.35 (3H, s), 3.65 (2H, s), 6.61 (1H, dd, *J* = 2 Hz, 1 Hz), 6.63 (1H, t, *J* = 2 Hz), 6.73 (1H, t, *J* = 2 Hz); ^13^C NMR (101 MHz, 25 degree, MeOH-*d*_4_) *σ*: 34.5, 54.6, 115.8, 116.4, 120.5, 135.9, 140.3, 160.4; *m*/*z*: (ESI^+^) 197.1 ([M + Na]^+^); *R*_f_: 0.1 (8 : 2 ethyl acetate : MeOH); purity: 93%.

##### 3-Bromo-5-((methylamino)methyl)phenol (15b)

White solid (82%); ^1^H NMR (400 MHz, 25 degree, MeOH-*d*_4_) *σ*: 2.39 (3H, s), 3.65 (2H, s), 6.72 (1H, s), 6.87 (1H, t, *J* = 2 Hz), 6.96 (1H, s); ^13^C NMR (101 MHz, 25 degree, MeOH-*d*_4_) *σ*: 35.1, 55.4, 115.8, 118.7, 123.3, 123.6, 142.7, 160.3; *m*/*z*: (ESI^+^) 216.0 ([M + H]^+^); *R*_f_: 0.1 (9 : 1 ethyl acetate : MeOH); purity: 95%.

##### 1-(3-Chloro-5-fluorophenyl)-*N*-methylmethanamine (17b)

White liquid (59%); ^1^H NMR (400 MHz, 25 degree, CDCl_3_) *σ*: 1.90 (1H, s), 2.42 (3H, s), 3.71 (2H, s), 6.95 (2H, dd, *J* = 9 Hz, 1 Hz), 7.11 (1H, s); ^13^C NMR (101 MHz, 25 degree, CDCl_3_) *σ*: 35.9, 55.0 (d, *J* = 2 Hz), 113.5 (d, *J* = 21 Hz), 114.8 (d, *J* = 25 Hz), 124.1 (d, *J* = 3 Hz), 135.0 (d, *J* = 11 Hz), 144.0 (d, *J* = 8 Hz), 162.9 (d, *J* = 249 Hz); *m*/*z*: (ESI^+^) 174.1 ([M + H]^+^); *R*_f_: 0.1 (ethyl acetate); purity: 96%.

##### 1-(3-Bromo-5-fluorophenyl)-*N*-methylmethanamine (18b)

White liquid (80%); ^1^H NMR (400 MHz, 25 degree, MeOH-*d*_4_) *σ*: 2.36 (3H, s), 3.70 (2H, s), 7.10 (1H, d, *J* = 9 Hz), 7.23 (1H, dt, *J* = 8 Hz, 2 Hz), 7.36 (1H, s); ^13^C NMR (101 MHz, 25 degree, MeOH-*d*_4_) *σ*: 34.0, 53.8, 113.9 (d, *J* = 22 Hz), 117.1 (d, *J* = 25 Hz), 122.1 (d, *J* = 10 Hz), 127.1 (d, *J* = 3 Hz), 144.0 (d, *J* = 8 Hz), 162.8 (d, *J* = 249 Hz); *m*/*z*: (ESI^+^) 217.9 ([M + H]^+^); *R*_f_: 0.1 (ethyl acetate); purity: 96%.

#### General method for the synthesis of final compounds

Fluconazole core (1 equiv.) and triethylamine (1.5 equiv.) were added into the solution of amine (1.5 equiv.) in ethanol (0.07 mmol mL^−1^) under stirring. The mixture was heated under stirring at 80 °C overnight until all starting material disappeared. The solvent was removed under reduced pressure and the product was purified by flash column chromatography.

##### 1-(Benzyl(methyl)amino)-2-(2,4-difluorophenyl)-3-(1*H*-1,2,4-triazol-1-yl)propan-2-ol (1)

White gel (54%); [*α*]^25^_D_: 10 (*c* = 2.0 in methanol); ^1^H NMR (400 MHz, 25 degree, MeOH-*d*_4_) *σ*: 2.12 (3H, s), 2.89 (1H, d, *J* = 14 Hz), 3.05 (1H, dd, *J* = 3.0 Hz, 1.2 Hz), 3.46 (1H, d, *J* = 13.0 Hz), 3.51 (1H, d, *J* = 13 Hz), 4.55 (2H, s), 6.82–6.93 (2H, m), 7.15–7.27 (5H, m), 7.52 (1H, td, *J* = 8.9 Hz, 6.9 Hz), 7.71 (1H, s), 8.28 (1H, s); ^13^C NMR (101 MHz, 25 degree, MeOH-*d*_4_) *σ*: 44.5, 57.5 (d, *J* = 5 Hz), 63.3 (d, *J* = 4 Hz), 64.5, 74.9 (d, *J* = 6 Hz), 104.9 (dd, *J* = 28 Hz, 26 Hz), 112.0 (dd, *J* = 21 Hz, 3.4 Hz), 127.5 (dd, *J* = 13 Hz, 4 Hz), 128.3, 129.3, 130.1, 131.0 (dd, *J* = 10 Hz, 6 Hz), 139.8, 146.0, 151.1, 160.3 (dd, *J* = 246 Hz, 12 Hz), 164.1 (dd, *J* = 248 Hz, 12 Hz); *m*/*z*: (ESI^+^) 359.1 ([M + H]^+^); HRMS: (ESI^+^) found 359.1672, ([M + H]^+^) requires 359.1678; *R*_f_: 0.2 (1 : 1 ethyl acetate : hexane); purity: 98%.

##### 2-(2,4-Difluorophenyl)-1-((4-fluorobenzyl)(methyl)amino)-3-(1*H*-1,2,4-triazol-1-yl)propan-2-ol (2)

Light yellow liquid (38%); [*α*]^25^_D_: 11 (*c* = 1.2 in methanol); ^1^H NMR (400 MHz, 25 degree, MeOH-*d*_4_) *σ*: 2.12 (3H, s), 2.87 (1H, d, *J* = 14 Hz), 3.05 (1H, dd, *J* = 14 Hz, 2 Hz), 3.46 (2H, s), 4.56 (2H, s), 6.82–6.93 (2H, m), 6.98 (2H, dd, *J* = 9 Hz, 6 Hz), 7.16 (2H, dd, *J* = 9 Hz, 6 Hz), 7.52 (1H, td, *J* = 9 Hz, 7 Hz), 7.72 (1H, s), 8.30 (1H, s); ^13^C NMR (101 MHz, 25 degree, MeOH-*d*_4_) *σ*: 44.4, 57.5 (dd, *J* = 5 Hz, 1 Hz), 63.2 (d, *J* = 4 Hz), 63.6, 75.1 (d, *J* = 6 Hz), 104.8 (dd, *J* = 26 Hz, 25 Hz), 112.0 (dd, *J* = 21 Hz, 3 Hz), 116.0 (d, *J* = 21 Hz), 127.4 (dd, *J* = 13 Hz, 4 Hz), 131.0 (dd, *J* = 9 Hz, 6 Hz), 131.8 (d, *J* = 8 Hz), 135.9 (d, *J* = 3 Hz), 146.0, 151.1, 160.6 (dd, *J* = 246 Hz, 12 Hz), 163.5 (d, *J* = 244 Hz), 164.1 (dd, *J* = 248 Hz, 12 Hz); *m*/*z*: (ESI^+^) 377.1 ([M + H]^+^); HRMS: (ESI^+^) found 377.1581, ([M + H]^+^) requires 377.15837; *R*_f_: 0.2 (1 : 1 DCM : ethyl acetate); purity: 98%.

##### 1-((4-Chlorobenzyl)(methyl)amino)-2-(2,4-difluorophenyl)-3-(1*H*-1,2,4-triazol-1-yl)propan-2-ol (3)

Yellow liquid (67%); [*α*]^24^_D_: −12 (*c* = 0.7 in methanol); ^1^H NMR (400 MHz, 25 degree, MeOH-*d*_4_) *σ*: 2.13 (3H, s), 2.87 (1H, d, *J* = 14 Hz), 3.06 (1H, dd, *J* = 14 Hz, 2 Hz), 3.47 (2H, s), 4.57 (2H, s), 6.82–6.92 (2H, m), 7.13 (2H, ddd, *J* = 9 Hz, 2 Hz, 2 Hz), 7.24 (2H, ddd, *J* = 9 Hz, 2 Hz, 2 Hz), 7.51 (1H, td, *J* = 9 Hz, 7 Hz), 7.72 (1H, s), 8.30 (1H, s); ^13^C NMR (101 MHz, 25 degree, MeOH-*d*_4_) *σ*: 44.5, 57.5 (d, *J* = 5 Hz), 63.3 (d, *J* = 5 Hz), 63.6, 75.2 (d, *J* = 5 Hz), 104.9 (dd, *J* = 28 Hz, 26 Hz), 112.0 (dd, *J* = 21 Hz, 3 Hz), 127.3 (dd, *J* = 13 Hz, 4 Hz), 129.37, 131.0 (dd, *J* = 10 Hz, 6 Hz), 131.6, 134.0, 138.7, 146.1, 151.1, 160.7 (dd, *J* = 246 Hz, 12 Hz), 164.2 (dd, *J* = 248 Hz, 12 Hz); *m*/*z*: (ESI^+^) 393.1 ([M + H]^+^); HRMS: (ESI^+^) found 393.13, ([M + H]^+^) requires 393.12882; *R*_f_: 0.2 (1 : 1 ethyl acetate : hexane); purity: 98%.

##### 1-((4-Bromobenzyl)(methyl)amino)-2-(2,4-difluorophenyl)-3-(1*H*-1,2,4-triazol-1-yl)propan-2-ol (4)

Whitish liquid (39%); [*α*]^25^_D_: 5 (*c* = 0.8 in methanol); ^1^H NMR (400 MHz, 25 degree, MeOH-*d*_4_) *σ*: 2.11 (3H, s), 2.86 (1H, d, *J* = 14 Hz), 3.05 (1H, dd, *J* = 14 Hz, 2 Hz), 3.44 (2H, s), 4.57 (2H, s), 6.81–6.91 (2H, m), 7.06 (2H, d, *J* = 8 Hz), 7.37 (2H, d, *J* = 8 Hz), 7.51 (1H, td, *J* = 9 Hz, 7 Hz), 7.72 (1H, s), 8.30 (1H, s); ^13^C NMR (101 MHz, 25 degree, MeOH-*d*_4_) *σ*: 44.5, 57.5 (d, *J* = 5 Hz), 63.3 (d, *J* = 4 Hz), 63.6, 75.2 (d, *J* = 6 Hz), 104.9 (dd, *J* = 28 Hz, 26 Hz), 112.0 (dd, *J* = 21 Hz, 3 Hz), 121.9, 127.3 (dd, *J* = 13 Hz, 4 Hz), 130.9 (dd, *J* = 10 Hz, 6 Hz), 131.9, 132.4, 139.2, 146.0, 151.1, 160.6 (dd, *J* = 246 Hz, 12 Hz), 164.1 (dd, *J* = 248 Hz, 12 Hz); *m*/*z*: (ESI^+^) 437.0 ([M + H]^+^); HRMS: (ESI^+^) found 437.0783, ([M + H]^+^) requires 437.0783; *R*_f_: 0.6 (ethyl acetate); purity: 98%.

##### 2-(2,4-Difluorophenyl)-1-((3-fluorobenzyl)(methyl)amino)-3-(1*H*-1,2,4-triazol-1-yl)propan-2-ol (5)

Off-white liquid (60%); [*α*]^23^_D_: 10 (*c* = 0.7 in methanol); ^1^H NMR (400 MHz, 25 degree, MeOH-*d*_4_) *σ*: 2.13 (3H, s), 2.86 (1H, d), 3.08 (1H, dd, *J* = 14 Hz, 2 Hz), 3.50 (2H, d, *J* = 2 Hz), 4.58 (2H, s), 6.83–6.97 (5H, m), 7.25 (1H, ddd, *J* = 6 Hz, 8 Hz, 8 Hz), 7.52 (1H, ddd, *J* = 7 Hz, 9 Hz, 9 Hz), 7.73 (1H, s), 8.29 (1H, s); ^13^C NMR (101 MHz, 25 degree, MeOH-*d*_4_) *σ*: 44.5, 57.5 (d, *J* = 5 Hz), 63.4 (d, *J* = 4 Hz), 63.8 (d, *J* = 2 Hz), 75.4 (d, *J* = 6 Hz), 104.9 (dd, *J* = 28 Hz, 26 Hz), 112.0 (dd, *J* = 21 Hz, 3 Hz), 114.9 (d, *J* = 21 Hz), 116.5 (d, *J* = 22 Hz), 125.7 (d, *J* = 3 Hz), 127.3 (dd, *J* = 13 Hz, 4 Hz), 130.9 (d, *J* = 8 Hz), 131.0 (dd, *J* = 10 Hz, 6 Hz), 143.0 (d, *J* = 7 Hz), 146.1, 151.1, 160.6 (dd, *J* = 246 Hz, 12 Hz), 164.2 (dd, *J* = 248 Hz, 12 Hz), 164.3 (d, *J* = 244 Hz); *m*/*z*: (ESI^+^) 377.1 ([M + H]^+^); HRMS: (ESI^+^) found 377.1578, ([M + H]^+^) requires 377.1584; *R*_f_: 0.3 (7 : 3 ethyl acetate : hexane); purity: 98%.

##### 1-((3-Chlorobenzyl)(methyl)amino)-2-(2,4-difluorophenyl)-3-(1*H*-1,2,4-triazol-1-yl)propan-2-ol (6)

Transparent liquid (52%); [*α*]^23^_D_: 11 (*c* = 0.5 in methanol); ^1^H NMR (400 MHz, 25 degree, MeOH-*d*_4_) *σ*: 2.13 (3H, s), 2.84 (1H, d, *J* = 14 Hz), 3.09 (1H, dd, *J* = 14 Hz, 2 Hz), 3.45 (1H, d, *J* = 13 Hz), 3.51 (1H, d, *J* = 13 Hz), 4.57 (2H, s), 6.82–6.93 (2H, m), 7.05–7.09 (2H, m), 7.17–7.23 (2H, m), 7.52 (1H, td, *J* = 9 Hz, 7 Hz), 7.74 (1H, s), 8.29 (1H, s); ^13^C NMR (101 MHz, 25 degree, MeOH-*d*_4_) *σ*: 44.5, 57.5 (d, *J* = 5 Hz), 63.5 (d, *J* = 4 Hz), 63.7, 75.5 (d, *J* = 6 Hz), 104.9 (dd, *J* = 28 Hz, 26 Hz), 112.0 (dd, *J* = 21 Hz, 3 Hz), 127.2 (dd, *J* = 13 Hz, 4 Hz), 128.3, 128.3, 129.9, 130.7, 131.0 (dd, *J* = 10 Hz, 6 Hz), 135.2, 142.5, 146.0, 151.1, 160.6 (dd, *J* = 246 Hz, 12 Hz), 164.1 (dd. *J* = 248 Hz, 12 Hz); *m*/*z*: (ESI^+^) 393.1 ([M + H]^+^); HRMS: (ESI^+^) found 393.1281, ([M + H]^+^) requires 393.1288; *R*_f_: 0.2 (1 : 1 ethyl acetate : hexane); purity: 98%.

##### 1-((3-Bromobenzyl)(methyl)amino)-2-(2,4-difluorophenyl)-3-(1*H*-1,2,4-triazol-1-yl)propan-2-ol (7)

White liquid (54%); [*α*]^25^_D_: 10 (*c* = 0.65 in methanol); ^1^H NMR (400 MHz, 25 degree, MeOH-*d*_4_) *σ*: 2.13 (3H, s), 2.83 (1H, d, *J* = 14 Hz), 3.10 (1H, dd, *J* = 14 Hz, 2 Hz), 3.44 (1H, d *J* = 13 Hz), 3.51 (1H, d, *J* = 13 Hz), 4.57 (2H, s), 6.83–6.93 (2H, m), 7.09–7.17 (2H, m), 7.24 (1H, s), 7.34 (1H, dt, *J* = 8 Hz, 2 Hz), 7.51 (1H, ddd, *J* = 7 Hz, 9 Hz, 9 Hz), 7.74 (1H, s), 8.29 (1H, s); ^13^C NMR (101 MHz, 25 degree, MeOH-*d*_4_) *σ*: 44.5, 57.5 (d, *J* = 5 Hz), 63.5 (d, *J* = 4 Hz), 63.6, 75.5 (d, *J* = 6 Hz), 104.9 (dd, *J* = 28 Hz, 26 Hz), 112.0 (dd, *J* = 21 Hz, 3 Hz), 123.3, 127.2 (dd, *J* = 13 Hz, 4 Hz), 128.7, 131.0 (dd, *J* = 9 Hz, 6 Hz), 131.0, 131.3, 132.9, 142.8, 146.0, 151.1, 160.6 (dd, *J* = 246 Hz, 12 Hz), 164.1 (dd, *J* = 248 Hz); *m*/*z*: (ESI^+^) 437.0 ([M + H]^+^); HRMS: (ESI^+^) found 437.0776, ([M + H]^+^) requires 437.0783; *R*_f_: 0.3 (4 : 6 ethyl acetate : hexane); purity: 98%.

##### 2-(((2-(2,4-Difluorophenyl)-2-hydroxy-3-(1*H*-1,2,4-triazol-1-yl)propyl)(methyl)amino)methyl)phenol (8)

Light white solid (43%); M.P. = 119–123 °C; [*α*]^24^_D_: 10 (*c* = 2.0 in methanol); ^1^H NMR (400 MHz, 25 degree, MeOH-*d*_4_) *σ*: 2.08 (3H, s), 2.89 (1H, d, *J* = 14 Hz), 3.21 (1H, dd, *J* = 14 Hz, 2 Hz), 3.60 (1H, d, *J* = 13 Hz), 3.68 (1H, d, *J* = 13 Hz), 4.56 (1H, d, *J* = 14 Hz), 4.64 (1H, d, *J* = 14 Hz), 6.67–6.74 (2H, m), 6.82–6.93 (2H, m), 6.96 (1H, dd, *J* = 8 Hz, 2 Hz), 7.08 (1H, td, *J* = 8 Hz, 2 Hz), 7.50 (1H, td, *J* = 9 Hz, 7 Hz), 7.76 (1H, s), 8.28 (1H, s); ^13^C NMR (101 MHz, 25 degree, MeOH-*d*_4_) *σ*: 43.6, 57.5 (d, *J* = 5 Hz), 62.5, 64.3 (d, *J* = 4 Hz), 76.0 (d, *J* = 6 Hz), 105.0 (dd, *J* = 28 Hz, 26 Hz), 112.2 (dd, *J* = 21 Hz, 3 Hz), 116.4, 120.3, 124.6, 126.2 (dd, *J* = 13 Hz, 4 Hz), 129.7, 130.9, 131.2 (dd, *J* = 10 Hz, 4 Hz), 145.9, 151.2, 157.7, 160.5 (dd, *J* = 246 Hz, 12 Hz), 164.2 (dd, *J* = 248 Hz, 12 Hz); *m*/*z*: (ESI^+^) 375.1 ([M + H]^+^); HRMS: (ESI^+^) found, 375.1625 ([M + H]^+^) requires 375.1627; *R*_f_: 0.2 (7 : 3 ethyl acetate : hexane); purity: 96%.

##### 3-(((2-(2,4-Difluorophenyl)-2-hydroxy-3-(1*H*-1,2,4-triazol-1-yl)propyl)(methyl)amino)methyl)phenol (9)

Transparent liquid (54%); [*α*]^25^_D_: 6 (*c* = 1.5 in methanol); ^1^H NMR (400 MHz, 25 degree, DMSO-*d*_6_) *σ*: 2.07 (3H, s), 2.77 (1H, d, *J* = 14 Hz), 2.97 (1H, d, *J* = 14 Hz), 3.35 (1H, d, *J* = 13 Hz), 3.44 (1H, d, *J* = 13 Hz), 4.51 (1H, d, *J* = 14 Hz), 4.57 (1H, d, *J* = 14 Hz), 5.71 (1H, s), 6.51 (1H, d, *J* = 8 Hz), 6.55 (1H, s), 6.60 (1H, dd, *J* = 8 Hz, 2 Hz), 6.95 (1H, td, *J* = 8 Hz, 2 Hz), 7.02 (1H, t, *J* = 8 Hz), 7.13 (1H, ddd, *J* = 12 Hz, 9 Hz, 2 Hz), 7.41 (1H, dd, *J* = 16 Hz, 9 Hz), 7.73 (1H, s), 8.29 (1H, s), 9.22 (1H, s); ^13^C NMR (101 MHz, DMSO) *δ* 193.64, 165.96, 163.57, 158.53, 157.71 (d), 154.74 (d), 152.06, 151.21, 150.85, 146.17, 145.56, 145.33, 144.47, 140.79, 130.51, 129.41 (d), 127.65 (t), 124.97 (d), 123.43, 120.07, 119.88, 119.63, 118.18, 117.30, 116.04, 114.30, 114.00, 113.75, 103.43, 103.20, 95.56, 95.29, 76.94, 76.45, 75.41, 75.36, 72.94, 69.63, 66.08, 63.52, 63.33, 63.28, 57.03, 56.30, 55.89, 53.87, 44.04, 40.39, 35.16; *m*/*z*: (ESI^+^) 375.1 ([M + H]^+^); HRMS: (ESI^+^) found 375.1623, ([M + H]^+^) requires 375.1627; *R*_f_: 0.1 (7 : 3 ethyl acetate : hexane); purity: 96%.

##### 2-(((2-(2,4-Difluorophenyl)-2-hydroxy-3-(1*H*-1,2,4-triazol-1-yl)propyl)(methyl)amino)methyl)-4-fluorophenol (10)

Off-white liquid (52%); [*α*]^25^_D_: 3 (*c* = 1.6 in methanol); ^1^H NMR (400 MHz, 25 degree, MeOH-*d*_4_) *σ*: 2.10 (3H, s), 2.89 (1H, d, *J* = 14 Hz), 3.22 (1H, d, *J* = 14 Hz), 3.57 (1H, d, *J* = 13 Hz), 3.67 (1H, d, *J* = 13 Hz), 4.57 (1H, d, *J* = 14 Hz), 4.64 (2H, d, *J* = 14 Hz), 6.64 (1H, dd, *J* = 9 Hz, 5 Hz), 6.74 (1H, dd, *J* = 9 Hz, 5 Hz), 6.80 (1H, td, *J* = 8 Hz, 2 Hz), 6.83–6.94 (2H, m), 7.50 (1H, td, *J* = 8 Hz, 6 Hz), 7.76 (1H, s), 8.28 (1H, s); ^13^C NMR (101 MHz, 25 degree, MeOH-*d*_4_) *σ*: 43.7, 57.5 (d, *J* = 5 Hz), 61.9 (d, *J* = 1 Hz), 64.2 (d, *J* = 4 Hz), 76.0, 76.0, 105.0 (dd, *J* = 28 Hz, 26 Hz), 112.2 (dd, *J* = 21 Hz, *J* = 4 Hz), 115.6 (d, *J* = 24 Hz), 117.0 (d, *J* = 22 Hz), 117.1 (d, *J* = 8 Hz), 126.1 (d, *J* = 7 Hz), 126.3 (dd, *J* = 13 Hz, 4 Hz); 160.6 (dd, *J* = 246 Hz, 12 Hz), 164.2 (dd, *J* = 248 Hz, 12 Hz); *m*/*z*: (ESI^+^) 393.1 ([M + H]^+^); HRMS: (ESI^+^) found 393.1526, ([M + H]^+^) requires 393.1533; *R*_f_: 0.5 (ethyl acetate); purity: 96%.

##### 4-Chloro-2-(((2-(2,4-difluorophenyl)-2-hydroxy-3-(1*H*-1,2,4-triazol-1-yl)propyl)(methyl)amino)methyl)phenol (11)

Transparent liquid (45%); [*α*]^25^_D_: 6 (*c* = 1.1 in methanol); ^1^H NMR (400 MHz, 25 degree, MeOH-*d*_4_) *σ*: 2.09 (3H, s), 2.87 (1H, d, *J* = 16 Hz), 3.21 (1H, dd, *J* = 15 Hz, 1 Hz), 3.55 (1H, d, *J* = 13 Hz), 3.66 (1H, d, *J* = 13 Hz), 4.57 (1H, d, *J* = 14 Hz), 4.63 (1H, d, *J* = 14 Hz), 6.65 (1H, d, *J* = 9 Hz), 6.83–6.94 (2H, m), 6.96 (1H, d, *J* = 3 Hz), 7.04 (1H, dd, *J* = 9 Hz, 3 Hz), 7.50 (1H, td, *J* = 9 Hz, 7 Hz), 7.77 (1H, s), 8.28 (1H, s); ^13^C NMR (101 MHz, 25 degree, MeOH-*d*_4_) *σ*: 43.7, 57.5 (d, *J* = 5 Hz), 61.7, 64.1(d, *J* = 4 Hz), 76.0 (d, *J* = 6 Hz), 105.0 (dd, *J* = 28 Hz, 26 Hz), 112.2 (dd, *J* = 21 Hz, 3 Hz), 117.8, 124.7, 126.2 (dd, *J* = 13 Hz, 4 Hz), 126.6, 129.3, 130.5, 131.2 (dd, J = 10 Hz, 6 Hz), 146.0, 151.3, 156.7, 160.5 (dd, *J* = 246 Hz, 12 Hz), 164.2 (dd, J = 248 Hz, 12 Hz); *m*/*z*: (ESI^+^) 409.1 ([M + H]^+^); HRMS: (ESI^+^) found 409.1234, ([M + H]^+^) requires 409.1237; *R*_f_: 0.5 (ethyl acetate); purity: 96%.

##### 4-Bromo-2-(((2-(2,4-difluorophenyl)-2-hydroxy-3-(1*H*-1,2,4-triazol-1-yl)propyl)(methyl)amino)methyl)phenol (12)

Transparent liquid (67%); [*α*]^25^_D_: 3 (*c* = 1.5 in methanol); ^1^H NMR (400 MHz, 25 degree, MeOH-*d*_4_) *σ*: 2.09 (3H, s), 2.87 (1H, d, *J* = 16 Hz), 3.22 (1H, dd, *J* = 15 Hz, 2 Hz), 3.56 (1H, d, *J* = 11 Hz), 3.67 (1H, d, *J* = 8 Hz), 4.57 (1H, d, *J* = 15 Hz), 4.63 (1H, d, *J* = 12 Hz), 6.60 (1H, d, *J* = 14 Hz), 6.86 (1H, td, *J* = 9 Hz, 2 Hz), 6.92 (1H, td, *J* = 11 Hz, 3 Hz), 7.10 (1H, d, *J* = 3 Hz), 7.18 (1H, dd, *J* = 9 Hz, 2 Hz), 7.50 (1H, td, *J* = 10 Hz, 9 Hz), 7.77 (1H, s), 8.28 (1H, s); ^13^C NMR (101 MHz, 25 degree, MeOH-*d*_4_) *σ*: 42.3, 56.1, 60.3, 62.7, 74.6, 103.6 (dd, *J* = 30 Hz, 22 Hz), 110.4, 110.8 (dd, *J* = 16 Hz, 8 Hz), 116.9, 124.9 (dd, *J* = 14 Hz, 4 Hz), 125.8, 129.8 (dd, *J* = 9 Hz, 4 Hz), 131.0, 132.0, 144.6, 149.9, 155.8, 159.2 (dd, *J* = 246 Hz, 12 Hz), 162.8 (dd, *J* = 248 Hz, 12 Hz); *m*/*z*: (ESI^+^) 453.0 ([M + H]^+^); HRMS: (ESI^+^) found 453.0729, ([M + H]^+^) requires 453.0732; *R*_f_: 0.1 (9 : 1 ethyl acetate : MeOH); purity: 98%.

##### 3-(((2-(2,4-Difluorophenyl)-2-hydroxy-3-(1*H*-1,2,4-triazol-1-yl)propyl)(methyl)amino)methyl)-5-fluorophenol (13)

Off-white liquid (50%); [*α*]^25^_D_: 3 (*c* = 1.0 in methanol); ^1^H NMR (400 MHz, 25 degree, DMSO-*d*_6_) *σ*: 2.07 (3H, s), 2.74 (1H, d, *J* = 12 Hz), 3.00 (1H, d, *J* = 15 Hz), 3.30 (1H, d, *J* = 11 Hz), 3.48 (1H, d, *J* = 15 Hz), 4.54 (2H, d, *J* = 5 Hz), 5.74 (1H, s), 6.31 (1H, dd, *J* = 44 Hz, 9 Hz), 6.38 (2H, s), 6.95 (1H, td, *J* = 13 Hz, 2 Hz), 7.13 (1H, td, *J* = 10 Hz, 2 Hz), 7.42 (1H, dd, *J* = 16 Hz, 11 Hz), 7.75 (1H, s), 8.29 (1H, s), 9.73 (1H, s); ^13^C NMR (101 MHz, 25 degree, MeOH-*d*_4_) *σ*: 44.6, 57.5 (d, *J* = 5 Hz), 63.4 (d, *J* = 3 Hz), 64.1, 75.1 (d, *J* = 6 Hz), 102.4 (d, *J* = 25 Hz), 104.9 (dd, *J* = 28 Hz, 26 Hz), 107.4 (d, *J* = 21 Hz), 112.0 (dd, *J* = 22 Hz, 3 Hz), 112.7, 127.4 (dd, *J* = 13 Hz, 4 Hz), 131.0 (dd, *J* = 10 Hz, 6 Hz), 143.3 (d, *J* = 9 Hz), 146.1, 151.1, 160.0, 160.6 (dd, *J* = 248 Hz, 12 Hz), 163.3 (dd, *J* = 248 Hz, 13 Hz) 165.0 (d, *J* = 241 Hz); *m*/*z*: (ESI^+^) 393.1 ([M + H]^+^); HRMS: (ESI^+^) found 393.1527, ([M + H]^+^) requires 393.1533; *R*_f_: 0.6 (ethyl acetate); purity: 97%.

##### 3-Chloro-5-(((2-(2,4-difluorophenyl)-2-hydroxy-3-(1*H*-1,2,4-triazol-1-yl)propyl)(methyl)amino)methyl)phenol (14)

Whitish liquid (53%); [*α*]^25^_D_: 4 (*c* = 1.2 in methanol); ^1^H NMR (400 MHz, 25 degree, MeOH-*d*_4_) *σ*: 2.13 (3H, s), 2.84 (1H, d, *J* = 14 Hz), 3.06 (1H, dd, *J* = 14 Hz, 2 Hz), 3.38 (2H, s), 4.58 (2H, d, *J* = 2 Hz), 6.53 (1H, dd, *J* = 2 Hz, 2 Hz), 6.57 (1H, t, *J* = 2 Hz), 6.64 (1H, t, *J* = 2 Hz), 6.82–6.93 (2H, m), 7.51 (1H, td, *J* = 9 Hz, 7 Hz), 7.74 (1H, s), 8.29 (1H, s); ^13^C NMR (101 MHz, 25 degree, MeOH-*d*_4_) *σ*: 44.6, 57.5 (d, *J* = 5 Hz), 63.4 (d, *J* = 4 Hz), 63.9, 75.2 (d, *J* = 6 Hz), 104.9 (dd, *J* = 28 Hz, 26 Hz), 112.0 (dd, *J* = 21 Hz, 3 Hz), 115.4, 115.5, 120.8, 127.3 (dd, *J* = 13 Hz, 4 Hz), 130.9 (dd, *J* = 10 Hz, 6 Hz), 135.5, 143.2, 146.1, 151.1, 159.7, 160.6 (dd, *J* = 246 Hz, 12 Hz), 164.1 (dd, *J* = 248 Hz, 12 Hz); *m*/*z*: (ESI^+^) 409.1 ([M + H]^+^); *R*_f_: 0.1 (ethyl acetate); purity: 95%.

##### 3-Bromo-5-(((2-(2,4-difluorophenyl)-2-hydroxy-3-(1*H*-1,2,4-triazol-1-yl)propyl)(methyl)amino)methyl)phenol (15)

Light yellow liquid (60%); [*α*]^25^_D_: −10 (*c* = 0.9 in methanol); ^1^H NMR (400 MHz, 25 degree, MeOH-*d*_4_) *σ*: 2.13 (3H, s), 2.84 (1H, d, *J* = 14 Hz), 3.06 (1H, d, *J* = 14 Hz), 3.39 (2H, s), 4.57 (2H, s), 6.57 (1H, s), 6.72 (1H, s), 6.80 (1H, s), 6.83–6.93 (2H, m), 7.51 (1H, td, *J* = 9 Hz, 7 Hz), 7.74 (1H, s), 8.30 (1H, s); ^13^C NMR (101 MHz, 25 degree, MeOH-*d*_4_) *σ*: 44.6, 57.5 (d, *J* = 7 Hz), 63.4 (d, *J* = 7 Hz), 63.8, 75.3 (d, *J* = 6 Hz), 104.9 (dd, *J* = 28 Hz, 26 Hz), 112.1 (dd, *J* = 21 Hz, 3 Hz), 115.9, 118.3, 123.4, 123.9, 127.2 (dd, *J* = 13 Hz, 4 Hz), 131.0 (dd, *J* = 10 Hz, 6 Hz), 143.4, 146.1, 151.1, 159.6, 160.6 (dd, *J* = 246 Hz, 12 Hz), 164.2 (dd, *J* = 248 Hz, 12 Hz); *m*/*z*: (ESI^+^) 453.0 ([M + H]^+^); HRMS: (ESI^+^) found 453.0725, ([M + H]^+^) requires 453.0732; *R*_f_: 0.6 (ethyl acetate); purity: 95%.

##### 1-((3,5-Difluorobenzyl)(methyl)amino)-2-(2,4-difluorophenyl)-3-(1*H*-1,2,4-triazol-1-yl)propan-2-ol (16)

Transparent liquid (48%); [*α*]^24^_D_: −15 (*c* = 0.6 in methanol); ^1^H NMR (400 MHz, 25 degree, MeOH-*d*_4_) *σ*: 2.15 (3H, s), 2.83 (1H, d, *J* = 14 Hz), 3.11 (1H, dd, *J* = 14 Hz, 2 Hz), 3.46 (1H, d, *J* = 14 Hz), 3.55 (1H, d, *J* = 14 Hz), 4.59 (2H, s), 6.71–6.77 (3H, m), 6.83–6.93 (2H, m), 7.52 (1H, td, *J* = 9 Hz, 7 Hz), 7.74 (1H, s), 8.30 (1H, s); ^13^C NMR (101 MHz, 25 degree, MeOH-*d*_4_) *σ*: 44.6, 57.5 (d, *J* = 5 Hz), 63.5 (t, *J* = 2 Hz), 63.6 (d, *J* = 4 Hz), 75.8 (d, *J* = 6 Hz), 103.2 (t, *J* = 26 Hz), 104.9 (dd, *J* = 26 Hz, 28 Hz), 112.0 (dd, *J* = 21 Hz, 8 Hz), 112.3 (dd, *J* = 19 Hz, 7 Hz), 127.1 (dd, *J* = 13 Hz, 4 Hz), 131.1 (dd, *J* = 10 Hz, 6 Hz), 145.1 (t, *J* = 9 Hz), 146.1, 151.2, 160.6 (dd, *J* = 246 Hz, 12 Hz), 164.2 (dd, *J* = 248 Hz, 12 Hz), 164.4 (dd, *J* = 247 Hz, 12 Hz); *m*/*z*: (ESI^+^) 395.1 ([M + H]^+^); HRMS: (ESI^+^) found 395.1484, ([M + H]^+^) requires 395.14895; *R*_f_: 0.3 (1 : 1 ethyl acetate : hexane); purity: 96%.

##### 1-((3-Chloro-5-fluorobenzyl)(methyl)amino)-2-(2,4-difluorophenyl)-3-(1*H*-1,2,4-triazol-1-yl)propan-2-ol (17)

Whitish liquid (50%); [*α*]^24^_D_: 11 (*c* = 0.5 in methanol); ^1^H NMR (400 MHz, 25 degree, MeOH-*d*_4_) *σ*: 2.14 (3H, s), 2.81 (1H, d, *J* = 14 Hz), 3.12 (1H, dd, *J* = 14 Hz, 2 Hz), 3.43 (1H, d, *J* = 14 Hz), 3.55 (1H, d, *J* = 14 Hz), 4.59 (2H, s), 6.80–6.92 (4H, m), 7.00 (1H, dt, *J* = 9 Hz, 2 Hz), 7.52 (1H, td, *J* = 9 Hz, 7 Hz), 7.75 (1H, s), 8.29 (1H, s); ^13^C NMR (101 MHz, 25 degree, MeOH-*d*_4_) *σ*: 44.6, 57.5 (d, *J* = 5 Hz), 63.3 (d, *J* = 2 Hz), 63.6 (d, *J* = 4 Hz), 75.9 (d, *J* = 6 Hz), 104.9 (dd, *J* = 28 Hz, 26 Hz), 112.0 (dd, *J* = 21 Hz, 3 Hz), 115.1 (d, *J* = 22 Hz), 115.6 (d, *J* = 25 Hz), 125.8 (d, *J* = 3 Hz), 127.1 (dd, *J* = 13 Hz, 4 Hz), 131.0 (dd, *J* = 9 Hz, 6 Hz), 135.8 (d, *J* = 11 Hz), 145.0 (d, *J* = 8 Hz), 146.1, 151.2, 160.6 (dd, *J* = 246 Hz, 11.9 Hz), 164.0 (d, *J* = 248 Hz), 164.2 (dd, *J* = 248 Hz, 12 Hz); *m*/*z*: (ESI^+^) 411.1 ([M + H]^+^); HRMS: (ESI^+^) found 411.1194, ([M + H]^+^) requires 411.11941; *R*_f_: 0.6 (ethyl acetate); purity: 95%.

##### 1-((3-Bromo-5-fluorobenzyl)(methyl)amino)-2-(2,4-difluorophenyl)-3-(1*H*-1,2,4-triazol-1-yl)propan-2-ol (18)

Off-white liquid (52%); [*α*]^24^_D_: 3 (*c* = 1.2 in methanol); ^1^H NMR (400 MHz, 25 degree, MeOH-*d*_4_) *σ*: 2.15 (3H, s), 2.80 (1H, d, *J* = 14 Hz), 3.13 (1H, dd, *J* = 14 Hz, 2 Hz), 3.43 (1H, d, *J* = 14 Hz), 3.56 (1H, d, *J* = 14 Hz), 4.59 (2H, s), 6.86 (2H, d, *J* = 9 Hz), 6.91 (1H, td, *J* = 10 Hz, 2 Hz), 7.06 (1H, s), 7.14 (1H, dt, *J* = 8 Hz, 2 Hz), 7.51 (1H, td, *J* = 9 Hz, 7 Hz), 7.76 (1H, s), 8.30 (1H, s); ^13^C NMR (101 MHz, 25 degree, MeOH-*d*_4_) *σ*: 44.6, 57.5 (d, *J* = 6 Hz), 63.1 (d, *J* = 2 Hz), 63.6 (d, *J* = 4 Hz), 76.0 (d, *J* = 6 Hz), 104.9 (dd, *J* = 28 Hz, 26 Hz), 112.0 (dd, *J* = 21 Hz, 4 Hz), 115.5 (d, *J* = 21 Hz), 118.5 (d, *J* = 25 Hz), 123.3 (d, *J* = 10 Hz), 127.1 (dd, *J* = 13 Hz, 4 Hz), 128.7 (d, *J* = 3 Hz), 131.1 (dd, *J* = 10 Hz, 6 Hz), 145.3 (d, *J* = 8 Hz), 146.1, 151.2, 160.7 (dd, *J* = 246 Hz, 12 Hz), 164.0 (d, *J* = 250 Hz), 164.2 (dd, *J* = 248 Hz, 12 Hz); *m*/*z*: (ESI^+^) 457.0 ([M + H]^+^); HRMS: (ESI^+^) found 457.0661, ([M + H]^+^) requires 457.0670; *R*_f_: 0.6 (ethyl acetate); purity: 98%.

### Molecular modelling

The ERG11-coded lanosterol 14α-demethylase structures used in the docking experiments were generated by AlphaFold 3.^32^ The heme cofactor was included in the CYP51 active site, and docking was configured to allow azole–heme coordination. To identify binding sites, 3D structures of known substrates and inhibitors were prepared. AutoDock SMINA was initially used to identify preferred binding pockets,^33^ and PyMOL was used to visualise inhibitor and substrate binding poses. The parameters were kept at default settings. After the locating of most favoured binding sites, GOLD was then used for final docking of the compounds into the selected binding sites.

### Chiral HPLC analysis

Samples were analysed by reverse-phase chiral HPLC using isocratic elution with varying ratios of water and acetonitrile (MeCN), with 0.1% formic acid added as an additive. Analyses were performed at a flow rate of 0.5 mL min^−1^, and eluted compounds were monitored by UV detection at 260 nm. Separations were carried out using a Daicel CHIRALPAK® AD-H analytical column (250 mm × 4.6 mm, 5 μm).

### MIC susceptibility tests

Microbroth MICs were conducted following EUCAST E.DEF 7.3.1 guidelines for yeast susceptibility testing. A 96-well plate was filled with 100 μL RPMI (MOPS) + 2% glucose in each well from columns 2 to 12. Stock solutions (1–5 mg mL^−1^) in DMSO were diluted to working solutions of 256–2 μg mL^−1^ in RPMI (MOPS) +2% glucose. Working solution (200 μL) was added to column 1 and serially diluted two-fold to column 11. Fungal strains, back-diluted from overnight cultures, were added to each well at a final concentration of 0.5–5 × 10^5^ CFU mL^−1^. The maximum DMSO concentration was 1.6% in the highest concentration well, with lower DMSO concentrations in subsequent dilutions; no growth inhibition was observed at these DMSO concentrations. Plates were incubated at 37 °C for 24 h, and growth was measured at OD530 nm using a FLUOstar Omega plate reader (BMG Labtech). For azole and azole-based antifungals, MIC was defined as the lowest concentration inhibiting ≥50% growth relative to the drug-free control. Tests were performed in at least three independent biological replicates, or until a modal MIC value was obtained where possible. MIC ranges in Tables 2 and 3 reflect replicate variation where a single modal MIC could not be assigned. Plates contained positive growth controls and negative media-only controls.

### *Galleria mellonella* assay

For toxicity testing, *G. mellonella* larvae were injected with 10 μL of antimicrobial agent or 10% DMSO in PBS in the first right proleg; 10 larvae were used per condition. For efficacy testing, larvae were injected with 10 μL of 1 × 10^7^ CFU mL^−1^*C. auris* TDG1912 in the first right proleg, incubated at room temperature for 30 min and then injected with 10 μL of antimicrobial agent or 10% DMSO vehicle control in the first left proleg. Controls were injected with PBS alone. For efficacy studies, 10 larvae were treated per group per repeat across three independent biological repeats, giving 30 larvae per treatment group; pooled survival data are shown in Fig. 5. *G. mellonella* were stored at 4 °C, allowed to reach room temperature for at least 1 h before use and used within 2 weeks of receipt. Larvae were incubated at 37 °C and assessed for survival daily for 5 days. Survival curves and *P* values were analysed using GraphPad Prism v9.5.0 and the Mantel–Cox log-rank test.

## Author contributions

YC, YL, KSN, CKH and KMR carried out experiments, YC, YL, CKH and KMR analysed data, CKH, JMS and KMR supervised the experiments, YL and KMR wrote the manuscript, All authors contributed to the edit and review of the manuscript. All authors agreed to the final version of the manuscript.

## Conflicts of interest

The authors declare no competing financial interest.

## Supplementary Material

MD-017-D6MD00330C-s001

## Data Availability

The data supporting this article have been included as part of the supplementary information (SI). Supplementary information: LC–MS method, experimental details for the synthesis of azoles, NMR spectra and HRMS, molecular formula strings (CSV). See DOI: https://doi.org/10.1039/d6md00330c.
