# Supplementary material for: Design, synthesis, and biological evaluation of azole antifungals against multidrug-resistant Candida auris
Source: RSC Med Chem. 2026 Jul 14. Online ahead of print. doi: 10.1039/d6md00330c (PMC13441049; doi:10.1039/d6md00330c)
Supplement: Supplementary file 1 [file MD-OLF-D6MD00330C-s001.pdf]

Supplementary Information (SI) for RSC Medicinal Chemistry.
This journal is © The Royal Society of Chemistry 2026

Design, Synthesis, and Biological Evaluation of Azole Antifungals Against Multidrug-resistant *Candida auris*

Yiyuan Chen[1], Yunxiao Li[1], Kazi S. Nahar[1,3], Charlotte K. Hind[2], J. Mark Sutton[1, 2] *, Khondaker Miraz Rahman[1] *

[1]Institute of Pharmaceutical Science, King's College London, 150 Stamford Street, London. SE1 9NH, United Kingdom.

[2]UK Health Security Agency, Countermeasures, Development, Evaluation and Preparedness, Manor Farm Road, Salisbury. SP4 0JG, United Kingdom.

[3]Department of Natural Sciences, University of Middlesex, The Burroughs, Hendon, London NW4 4BT

***Corresponding authors:**

JMS: Tel.: +44 (0) 1980 612649; e-mail: mark.sutton@phe.gov.uk

KMR: Tel: +44 (0) 2078 481891; e-mail: k.miraz.rahman@kcl.ac.uk

Supporting Information

Purity determination of synthesized final compounds

The level of purity of the compounds for biological testing has been evaluated through LC-MS analysis, using two different gradient methods, reported hereafter. LC-MS analyses were performed on a Waters Alliance 2695 system (from Waters), with elution in gradient. HPLC grade solvents were used as mobile phase while a Monolithic C18 50 X 4.60 mm column (from Phenomenex) was used as stationary phase. UV detection was performed using a Waters 2996 photo array detector (from Waters). Injection volume has been set to 10 µL. The compounds have been dissolved in a mixture of H2O/ACN (50/50, v/v) or DMSO/ACN (50/50, v/v) accordingly to the solubility. The area of the peak corresponding to the compound has been automatically determined by the software included in the LC-MS system. The eventual presence of solvent UV trace has been subtracted to the total in order to determine the percentage of purity. All compounds showed at least 95% purity in both methods.

LC-MS methods:

Method A: flow 0.5 mL/min

A) water + 0.1 % formic acid

B) acetonitrile + 0.1% formic acid

Time (min)	0	3	3.5	4.5	5
A (%)	95	10	5	5	95
B (%)	5	90	95	95	5

Method B: flow 1 mL/min

A) water + 0.1 % formic acid

B) acetonitrile + 0.1% formic acid

Time (min)	0	2	5	6	7.5	9	10
A (%)	95	95	50	50	5	95	95
B (%)	5	5	50	50	95	5	5

NMR and MS Spectra of Final Compounds

1-(benzyl(methyl)amino)-2-(2,4-difluorophenyl)-3-(1H-1,2,4-triazol-1-yl)propan-2-ol (1)

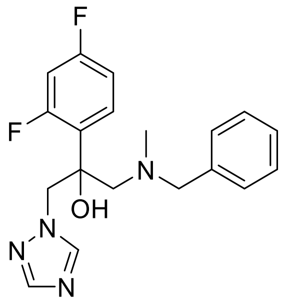

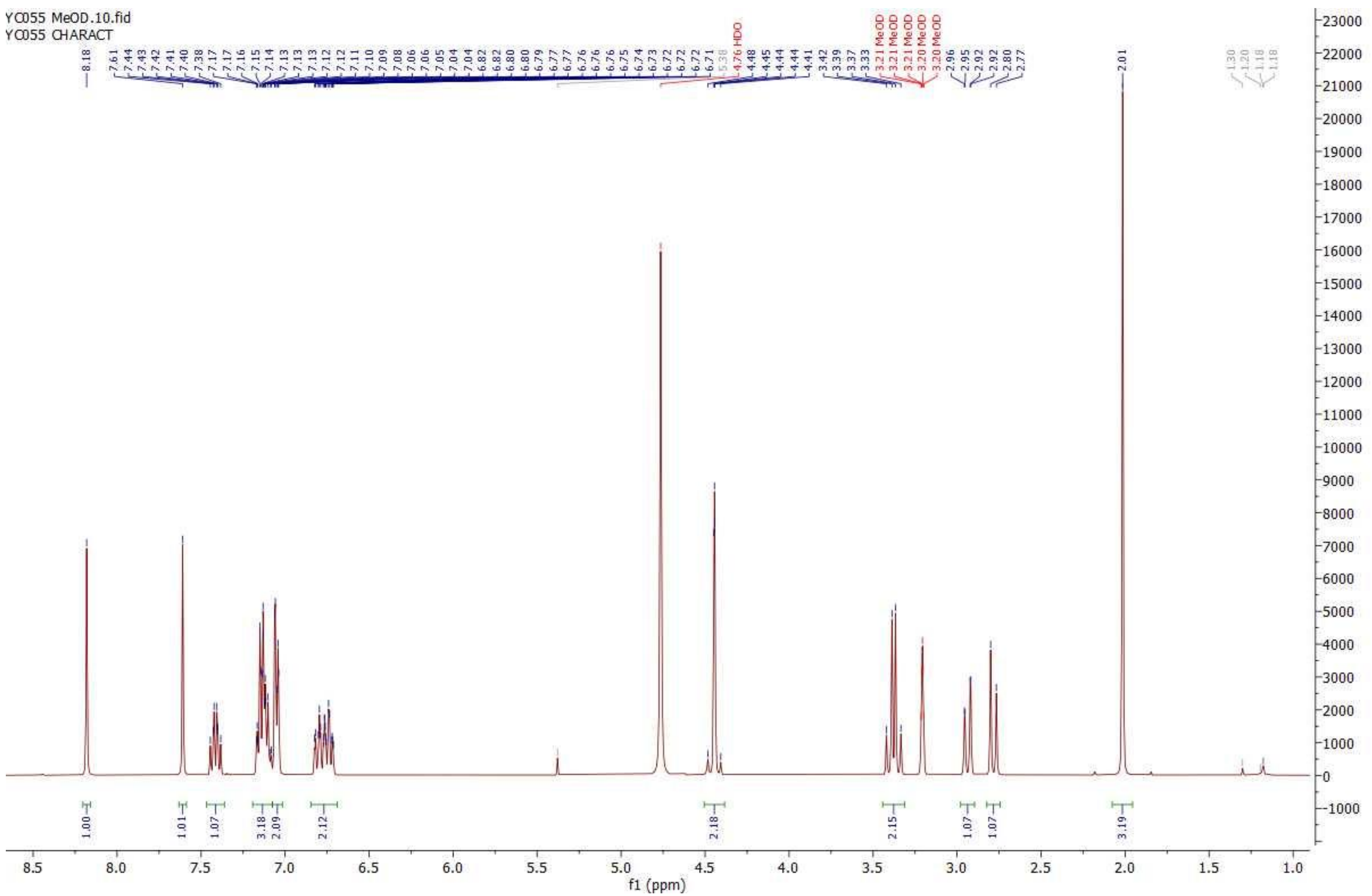

Figure S1-1: Proton ^{1}H NMR spectrum of Compound **1.**

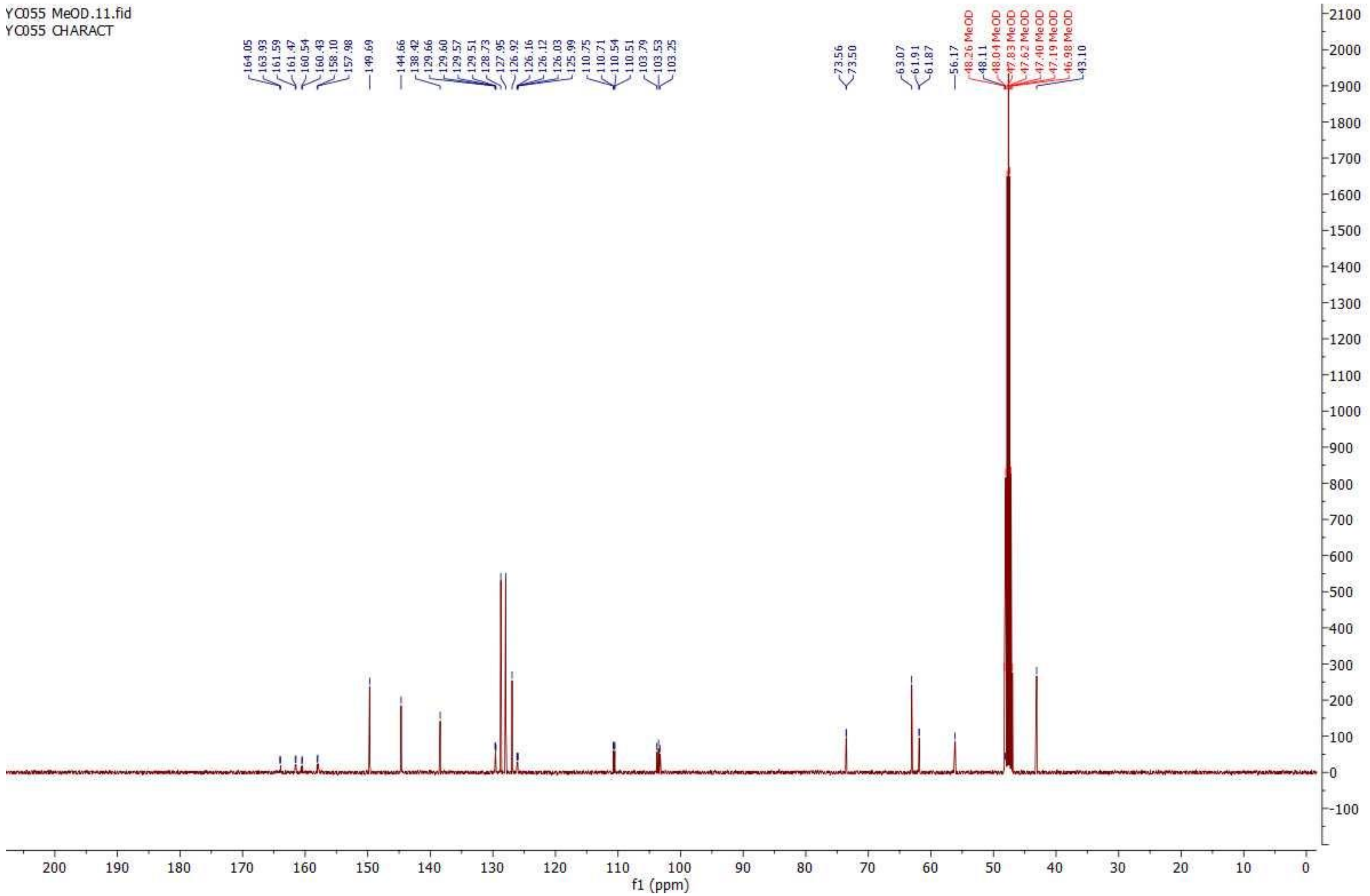

Figure S1-2: Carbon ^{13}C NMR spectrum of Compound **1.**

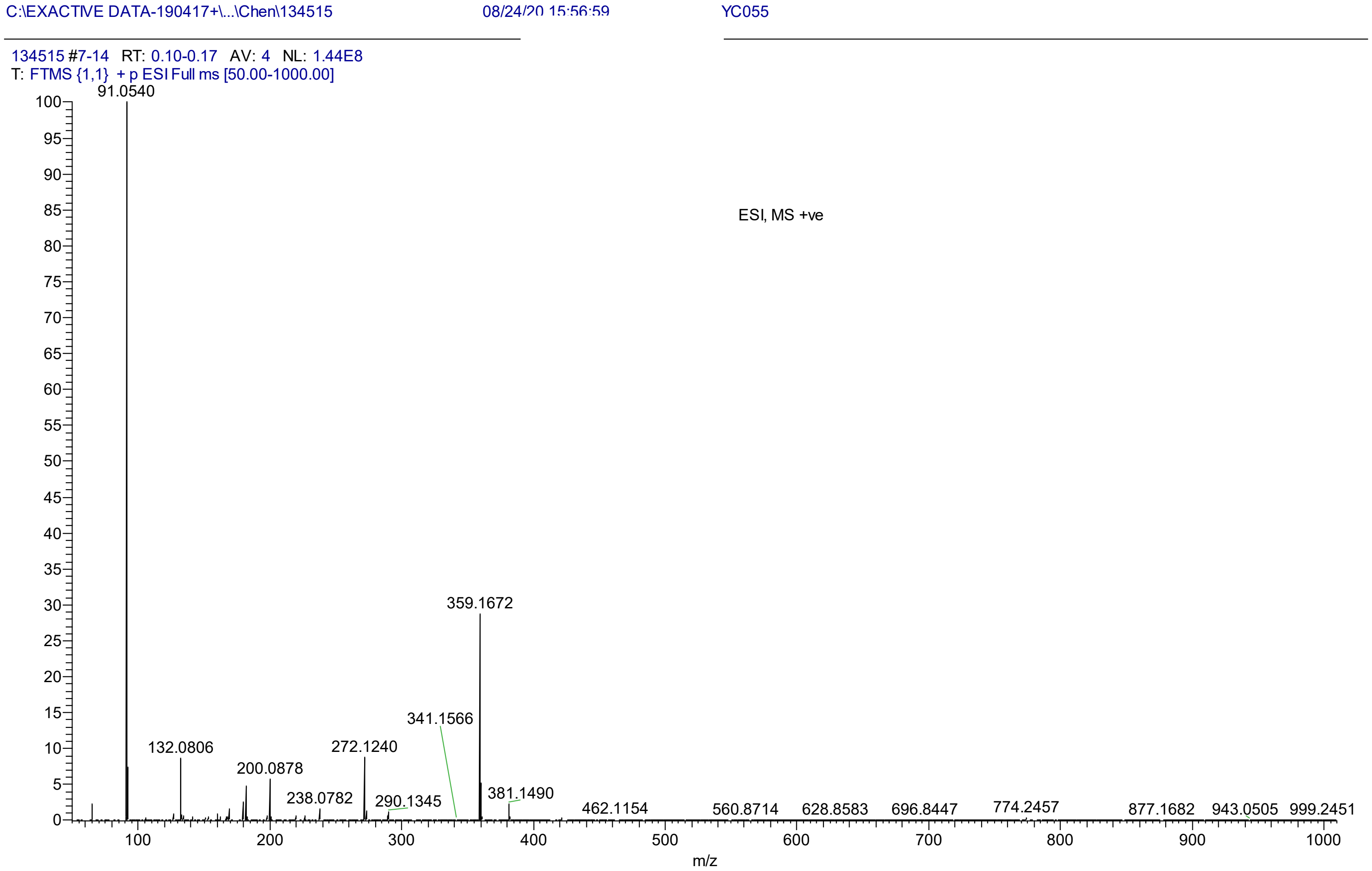

Figure S1-3: HRMS spectrum of Compound **1.**

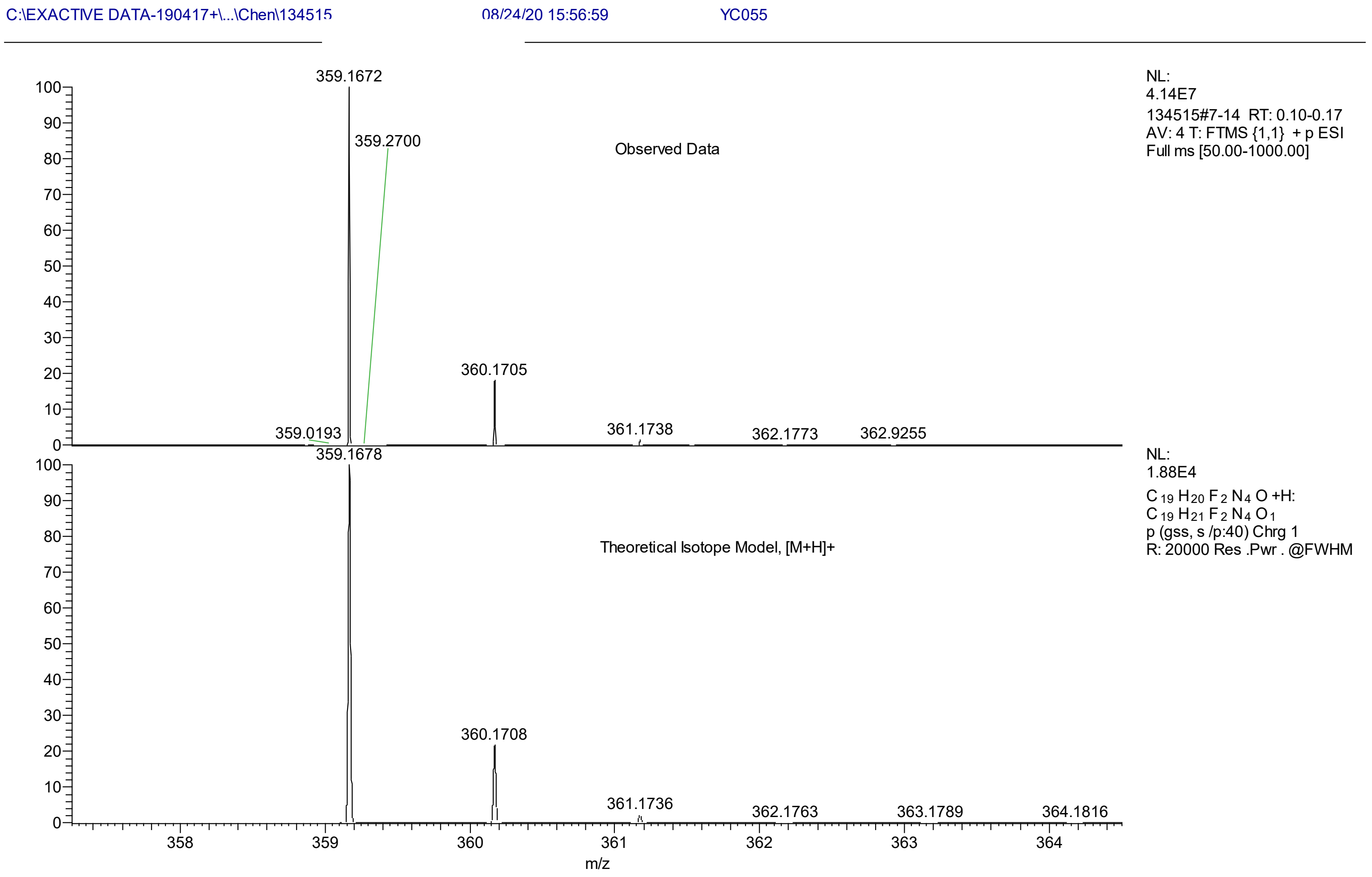

Figure S1-4: HRMS spectrum of Compound **1.**

2-(2,4-difluorophenyl)-1-((4-fluorobenzyl)(methyl)amino)-3-(1H-1,2,4-triazol-1-yl)propan-2-ol (2)

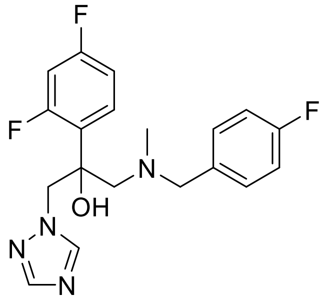

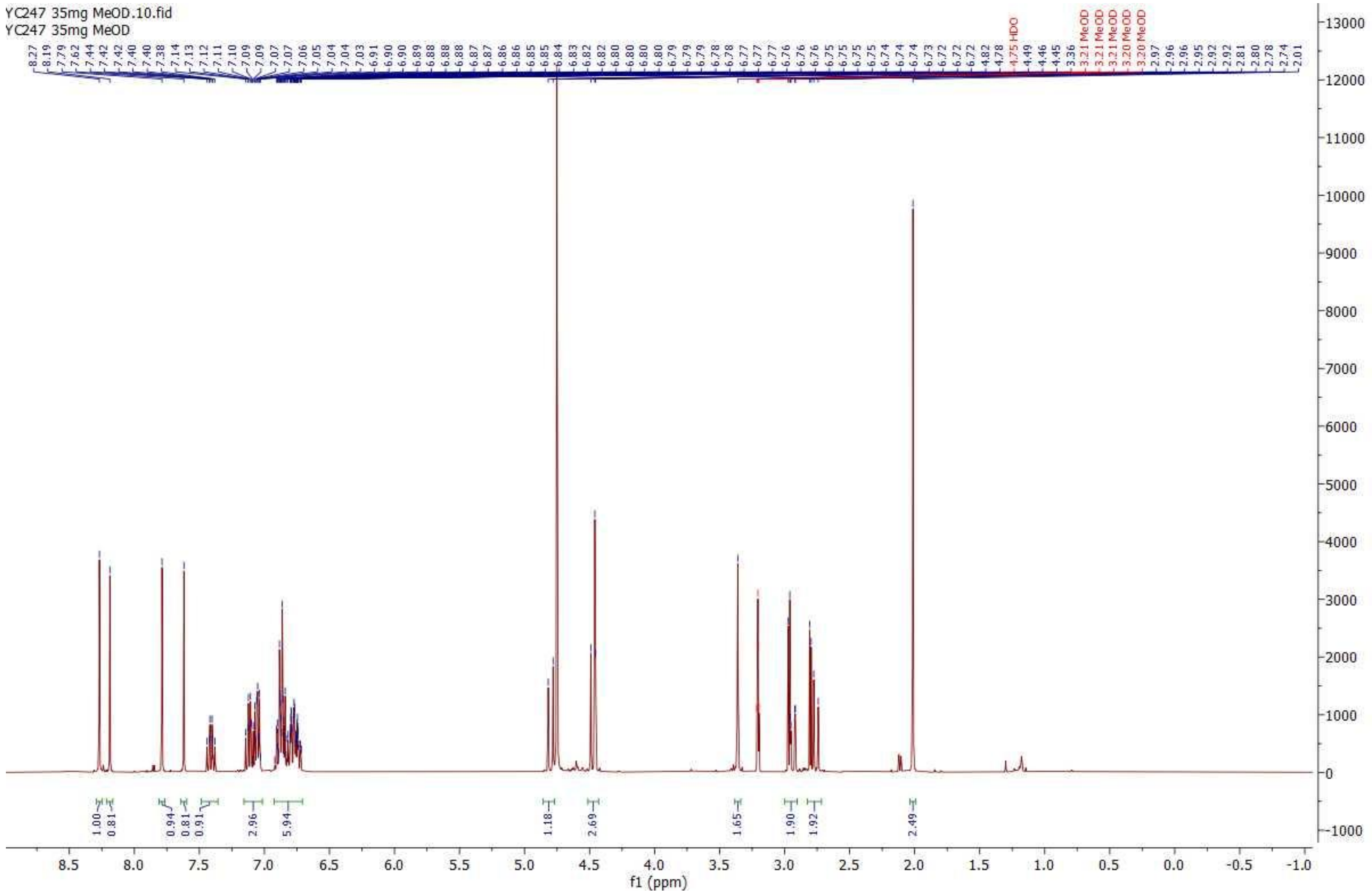

Figure S2-1: Proton ^{1}H NMR spectrum of Compound **2.**

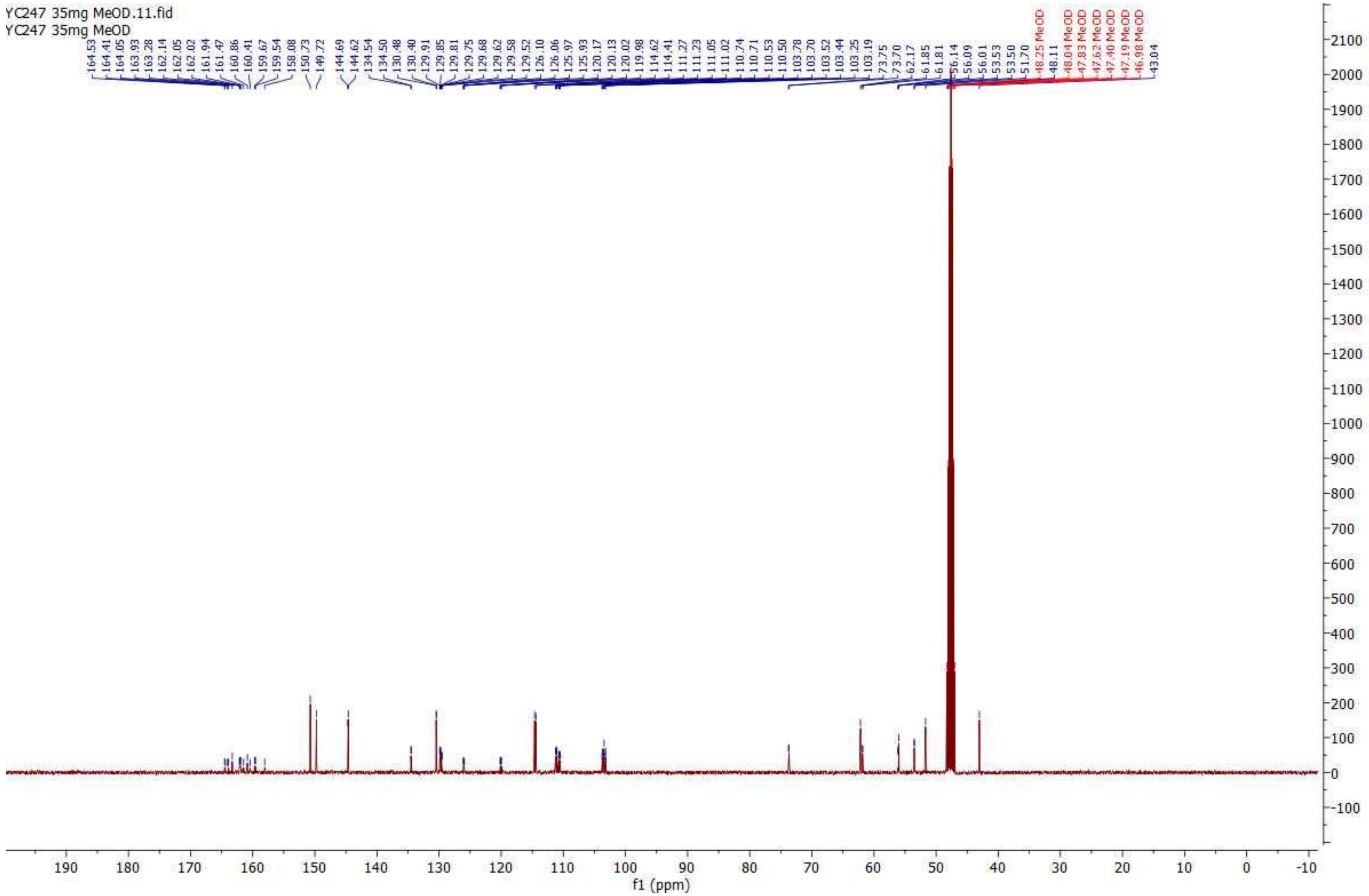

Figure S2-2: Carbon ^{13}C NMR spectrum of Compound **2.**

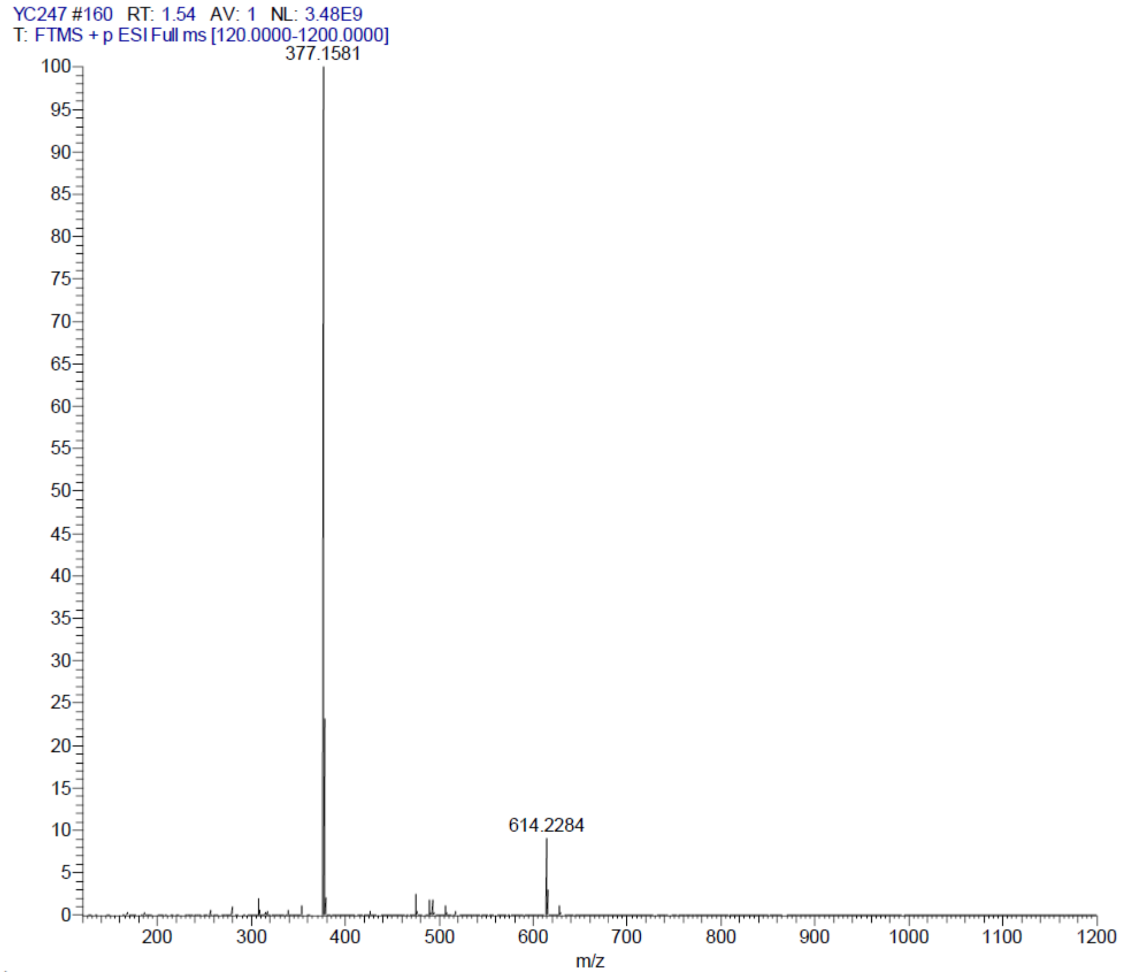

Figure S2-3: HRMS spectrum of Compound **2.**

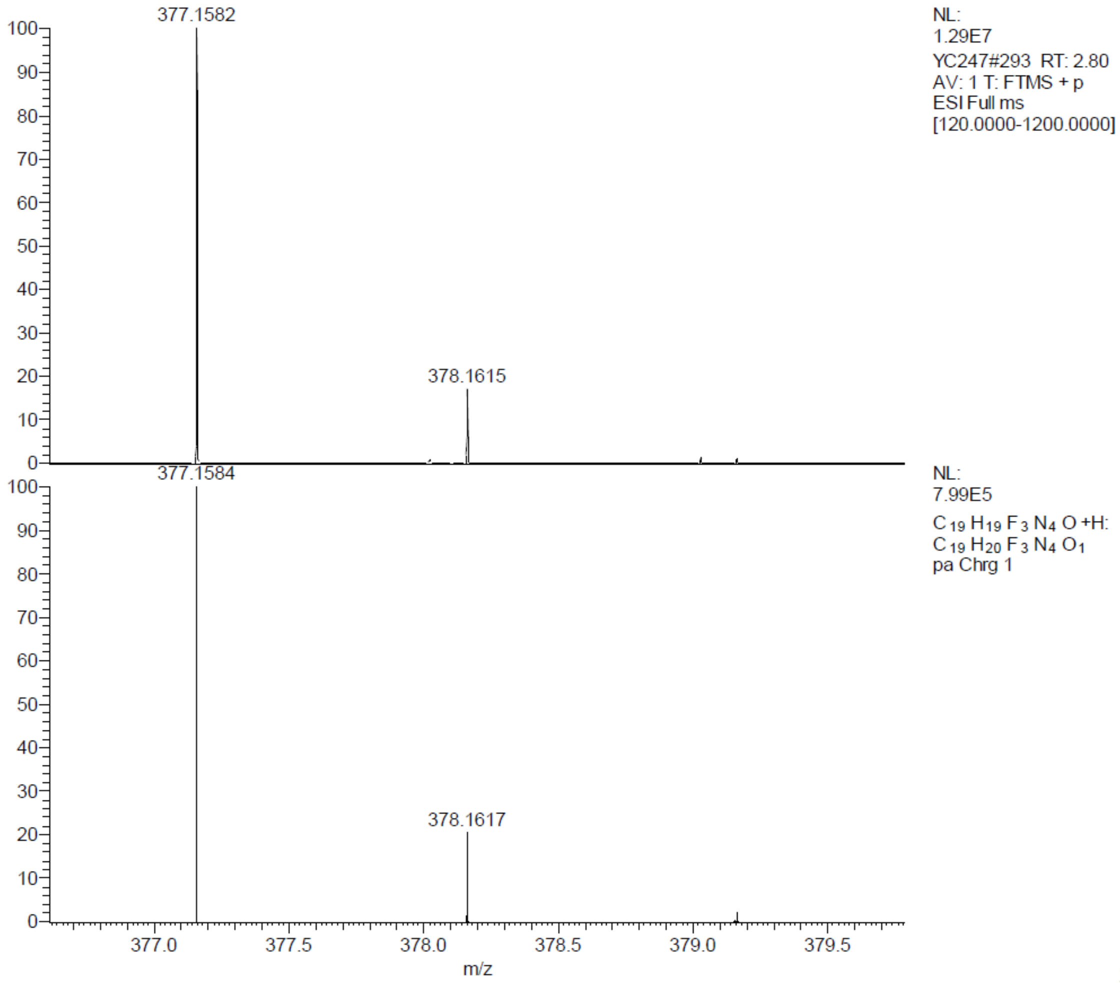

Figure S2-4: HRMS spectrum of Compound **2.**

1-((4-chlorobenzyl)(methyl)amino)-2-(2,4-difluorophenyl)-3-(1H-1,2,4-triazol-1-yl)propan-2-ol (3)

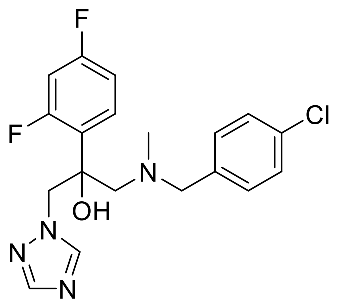

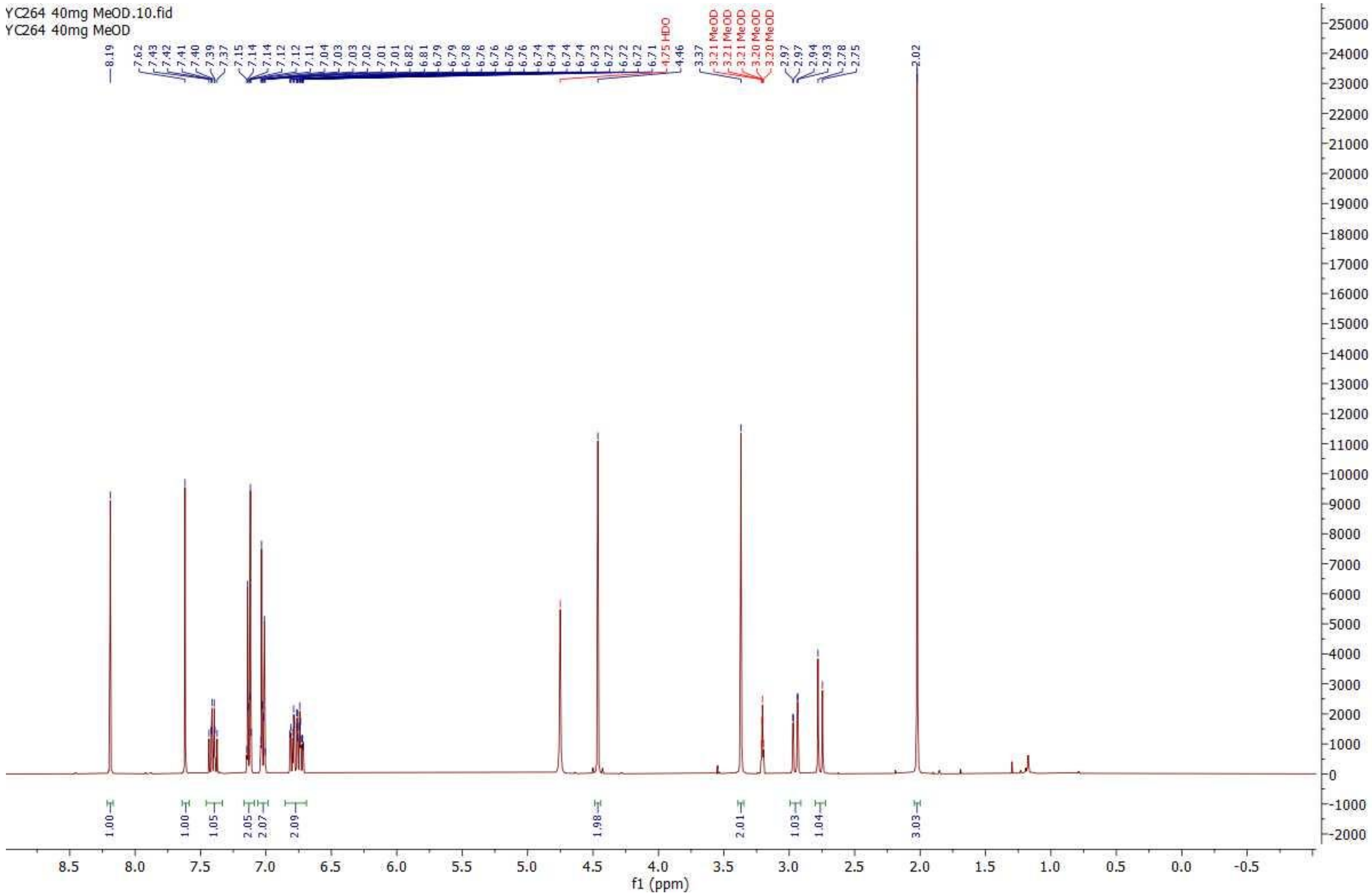

Figure S3-1: Proton ^{1}H NMR spectrum of Compound **3.**

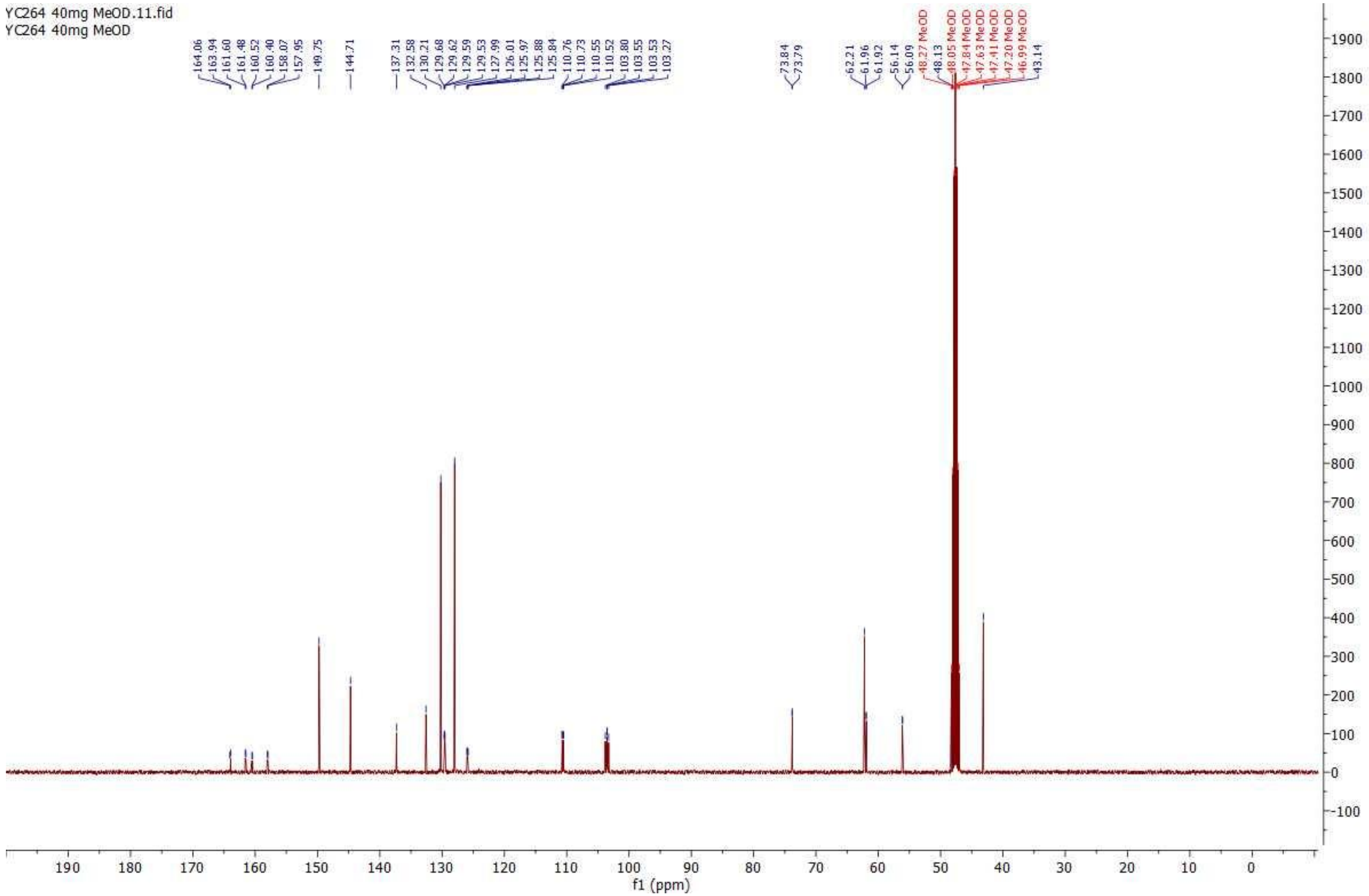

Figure S3-2: Carbon ^{13}C NMR spectrum of Compound **3.**

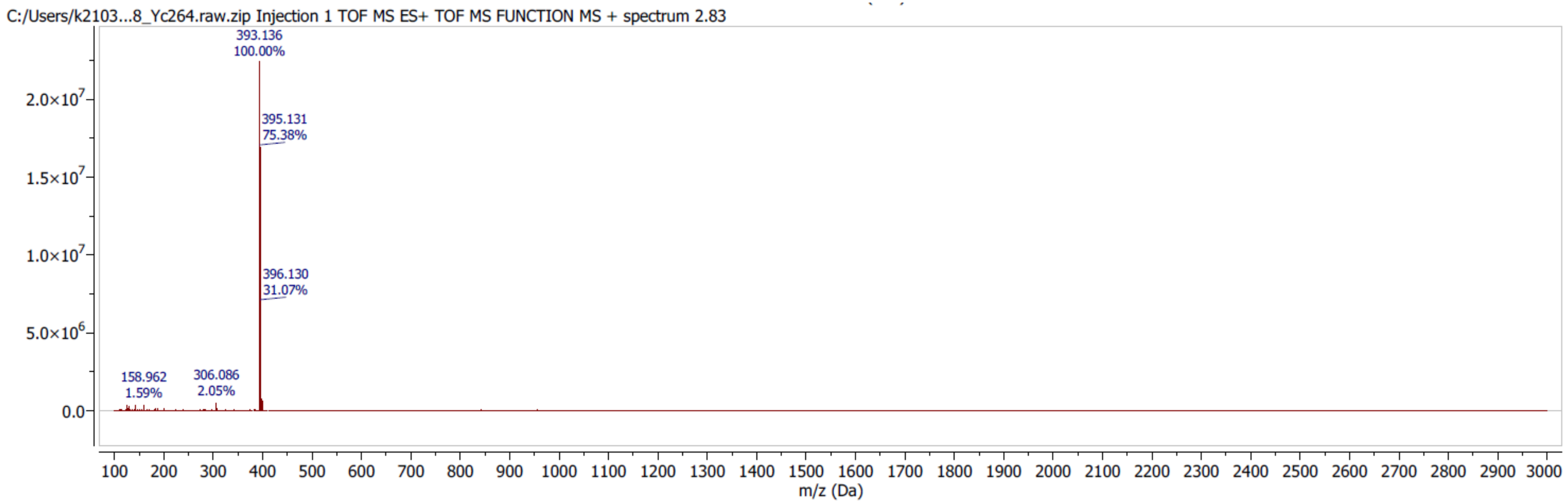

Figure S3-3: HRMS spectrum of Compound **3.**

1-((4-bromobenzyl)(methyl)amino)-2-(2,4-difluorophenyl)-3-(1H-1,2,4-triazol-1-yl)propan-2-ol (4)

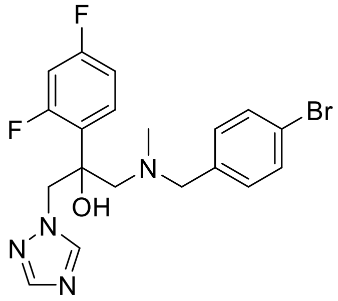

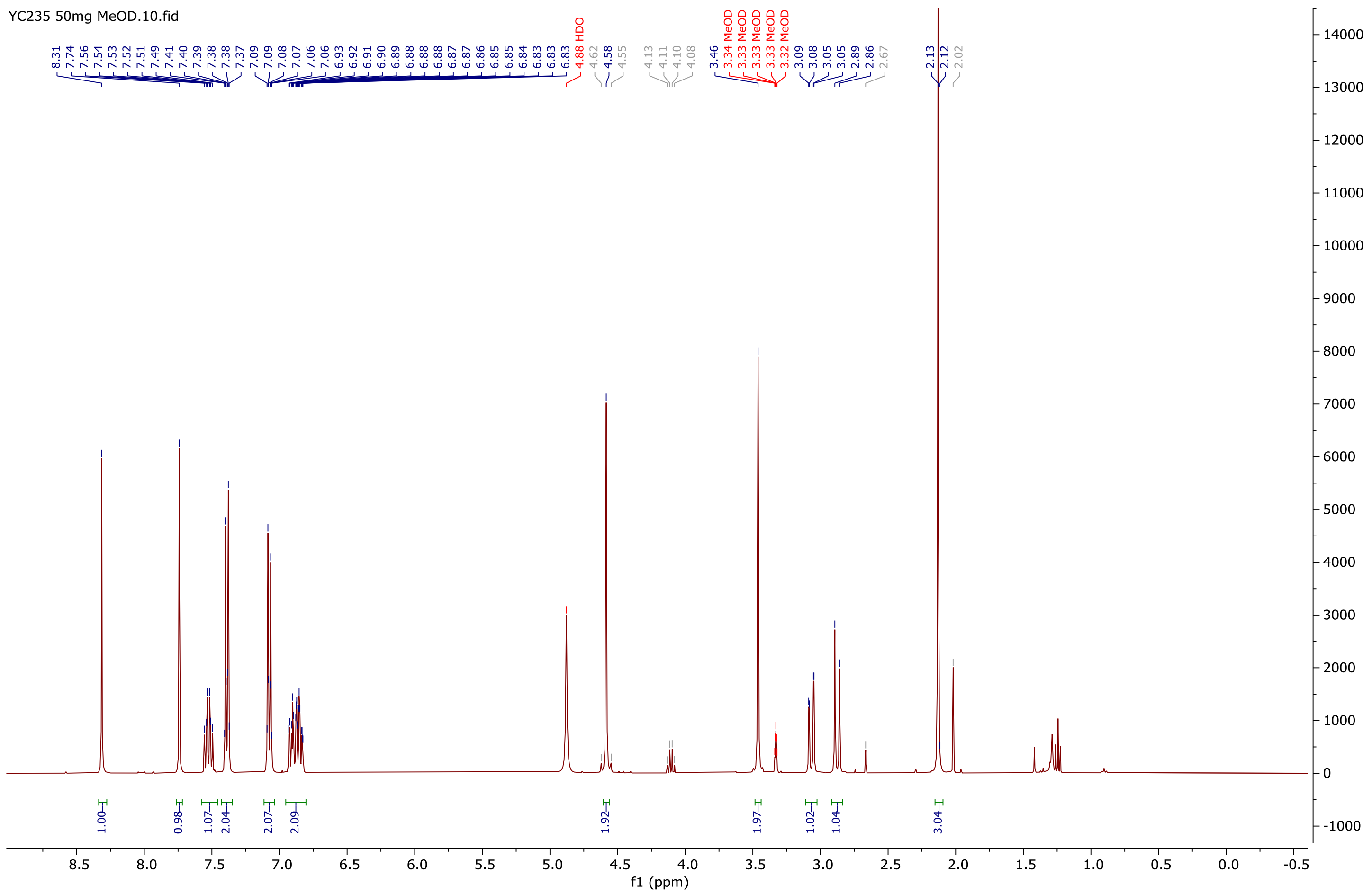

Figure S4-1: Proton ^{1}H NMR spectrum of Compound **4.**

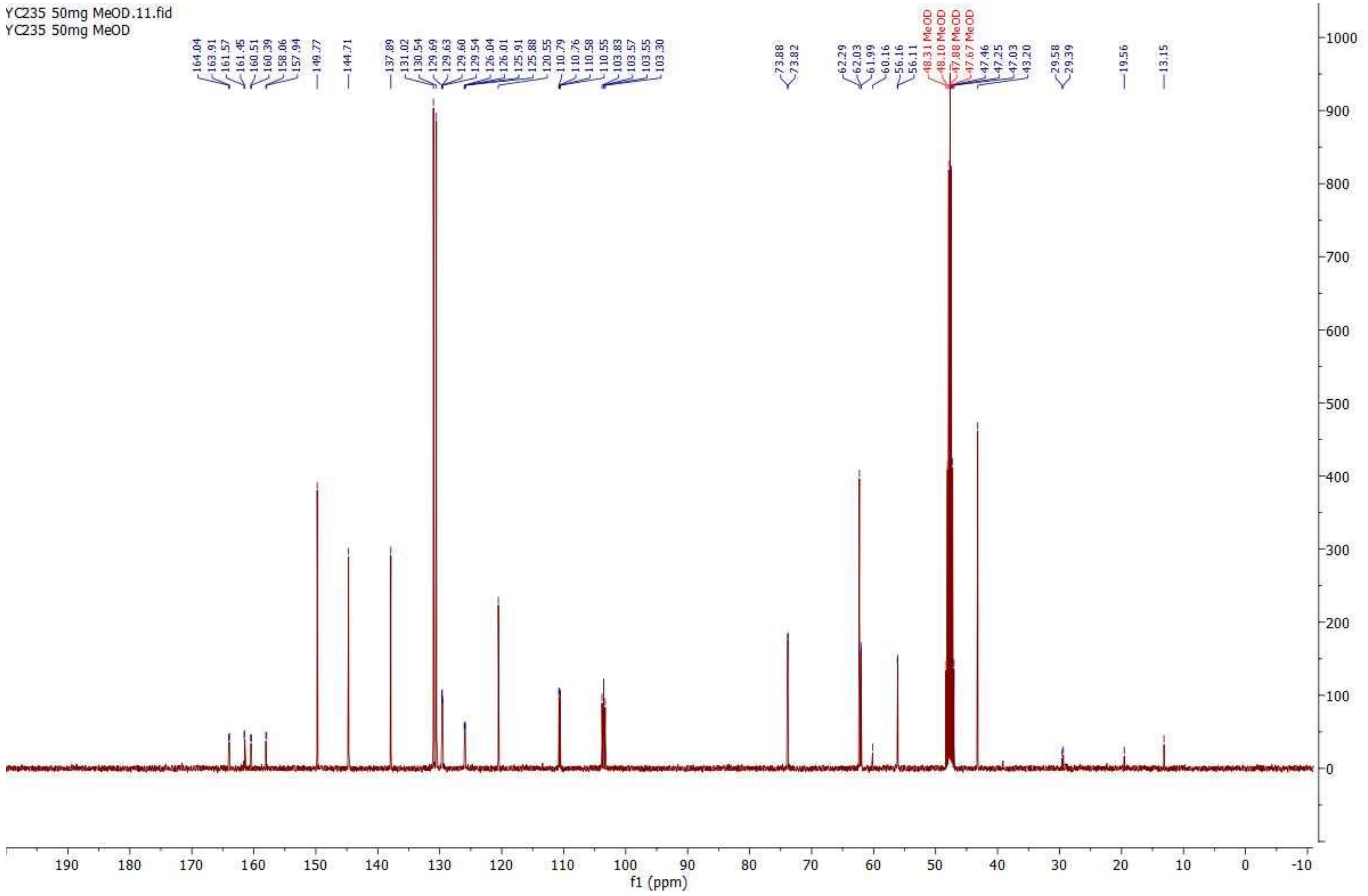

Figure S4-2: Carbon ^{13}C NMR spectrum of Compound **4.**

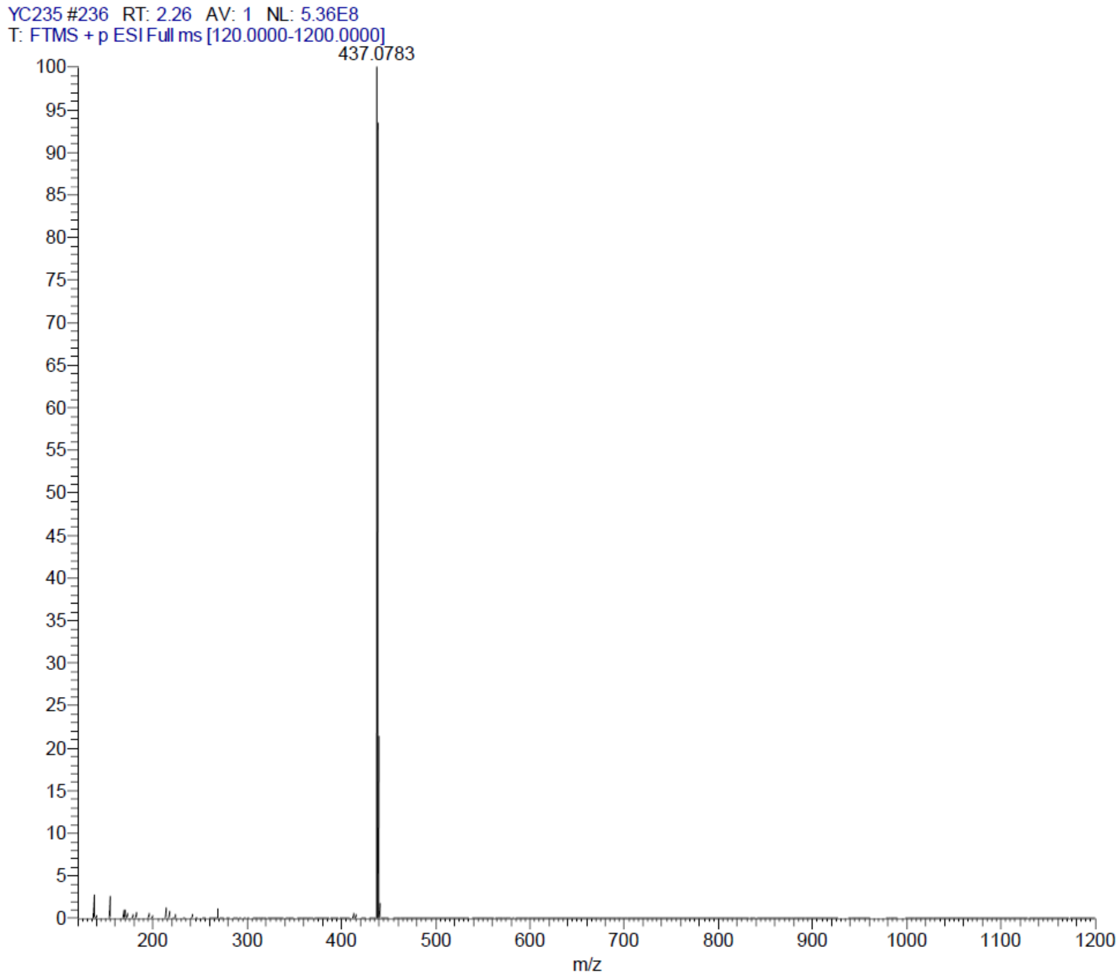

Figure S4-3: HRMS spectrum of Compound **4.**

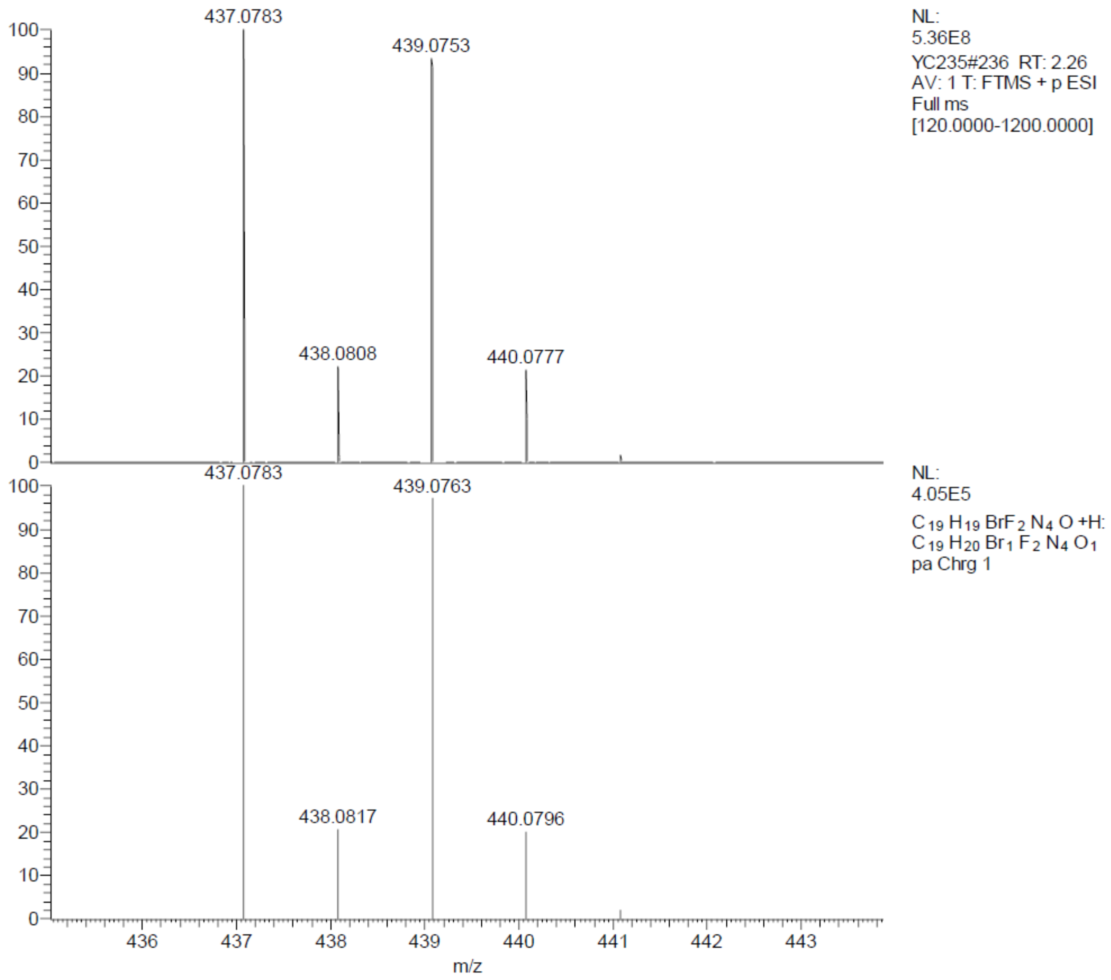

Figure S4-4: HRMS spectrum of Compound **4.**

2-(2,4-difluorophenyl)-1-((3-fluorobenzyl)(methyl)amino)-3-(1H-1,2,4-triazol-1-yl)propan-2-ol (5)

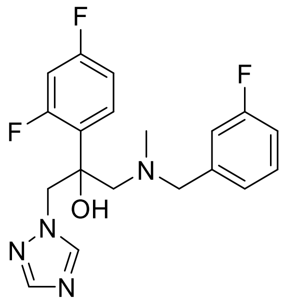

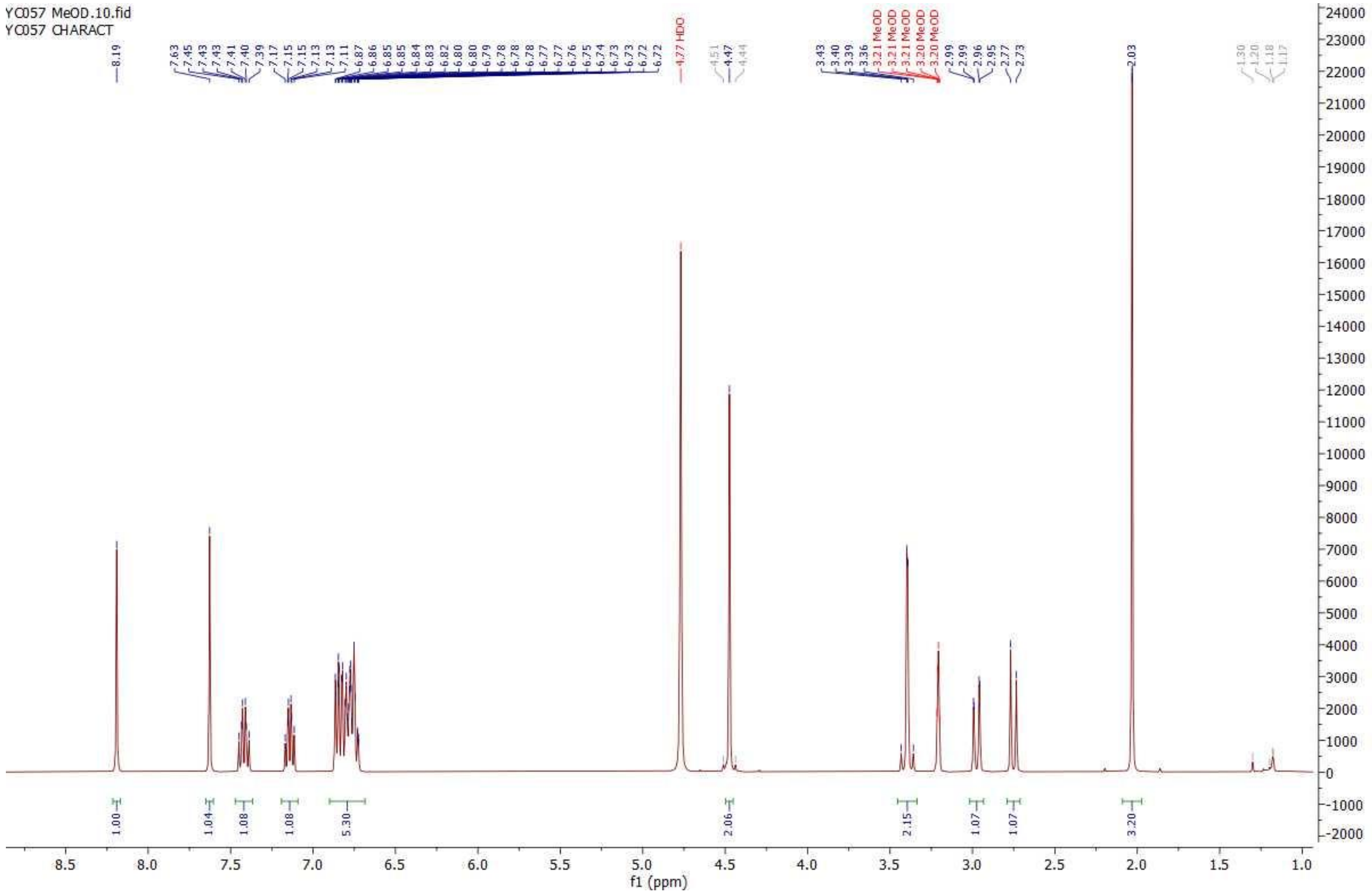

Figure S5-1: Proton ^{1}H NMR spectrum of Compound **5.**

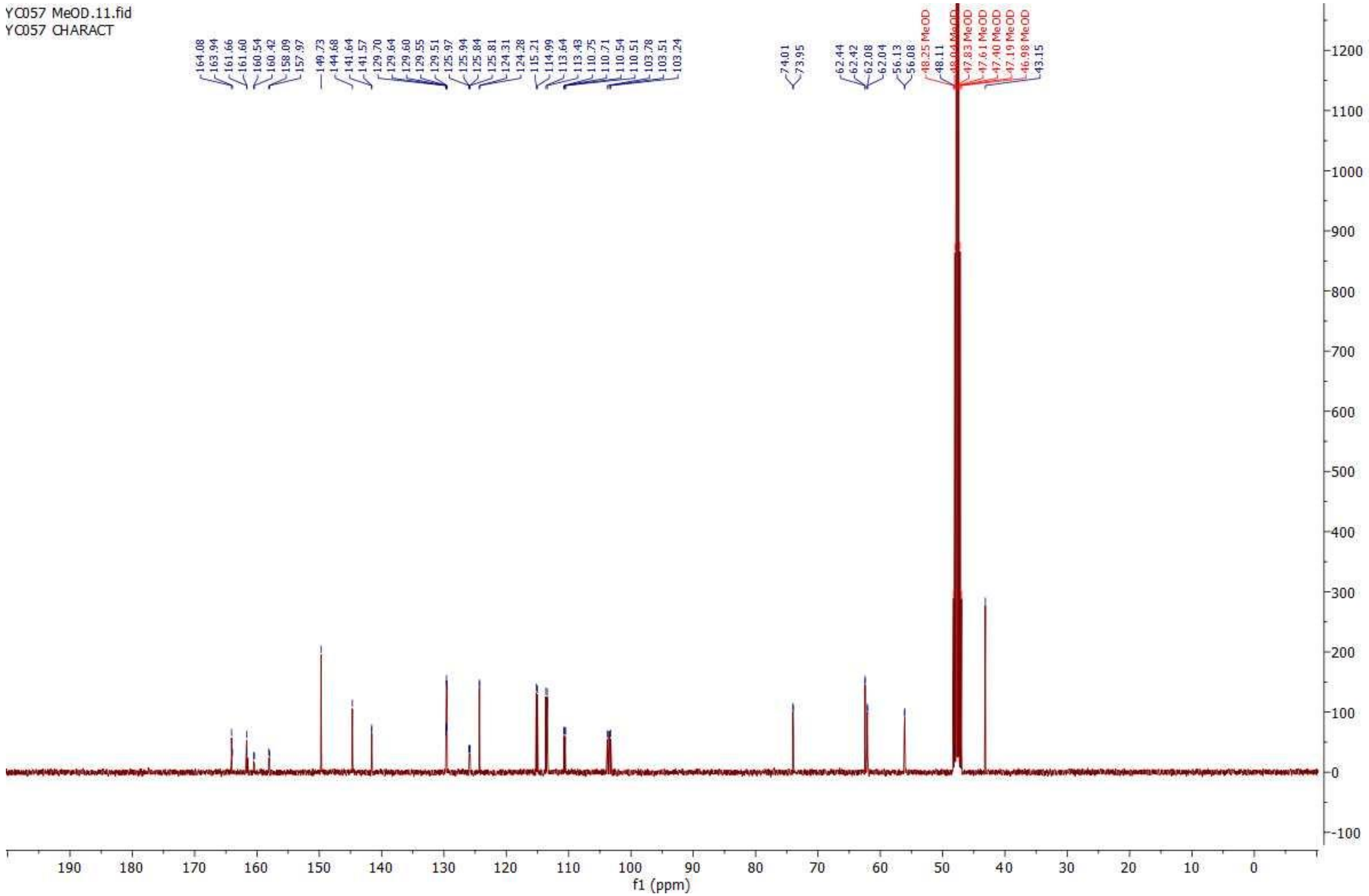

Figure S5-2: Carbon ^{13}C NMR spectrum of Compound **5.**

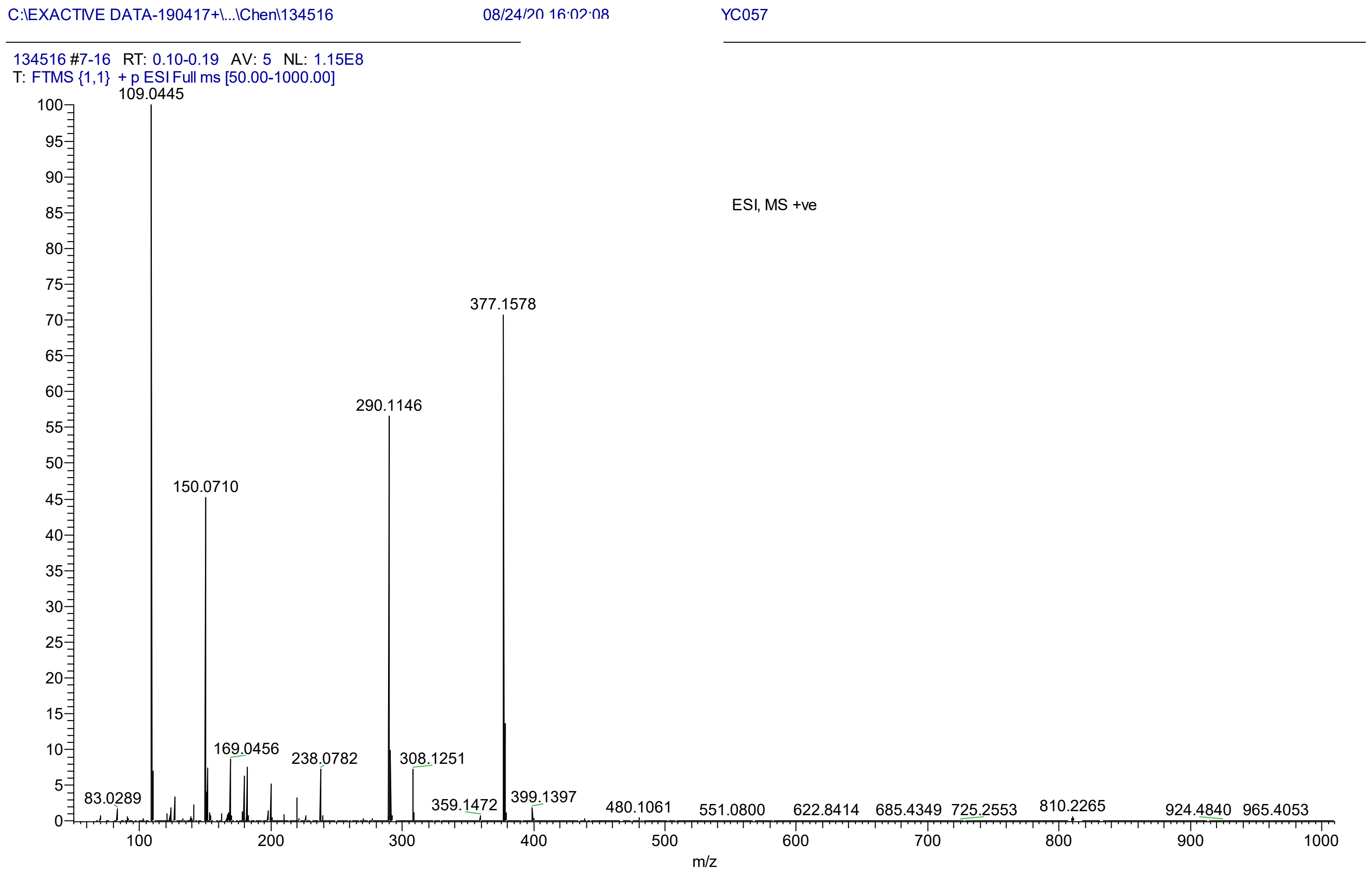

Figure S5-3: HRMS spectrum of Compound **5.**

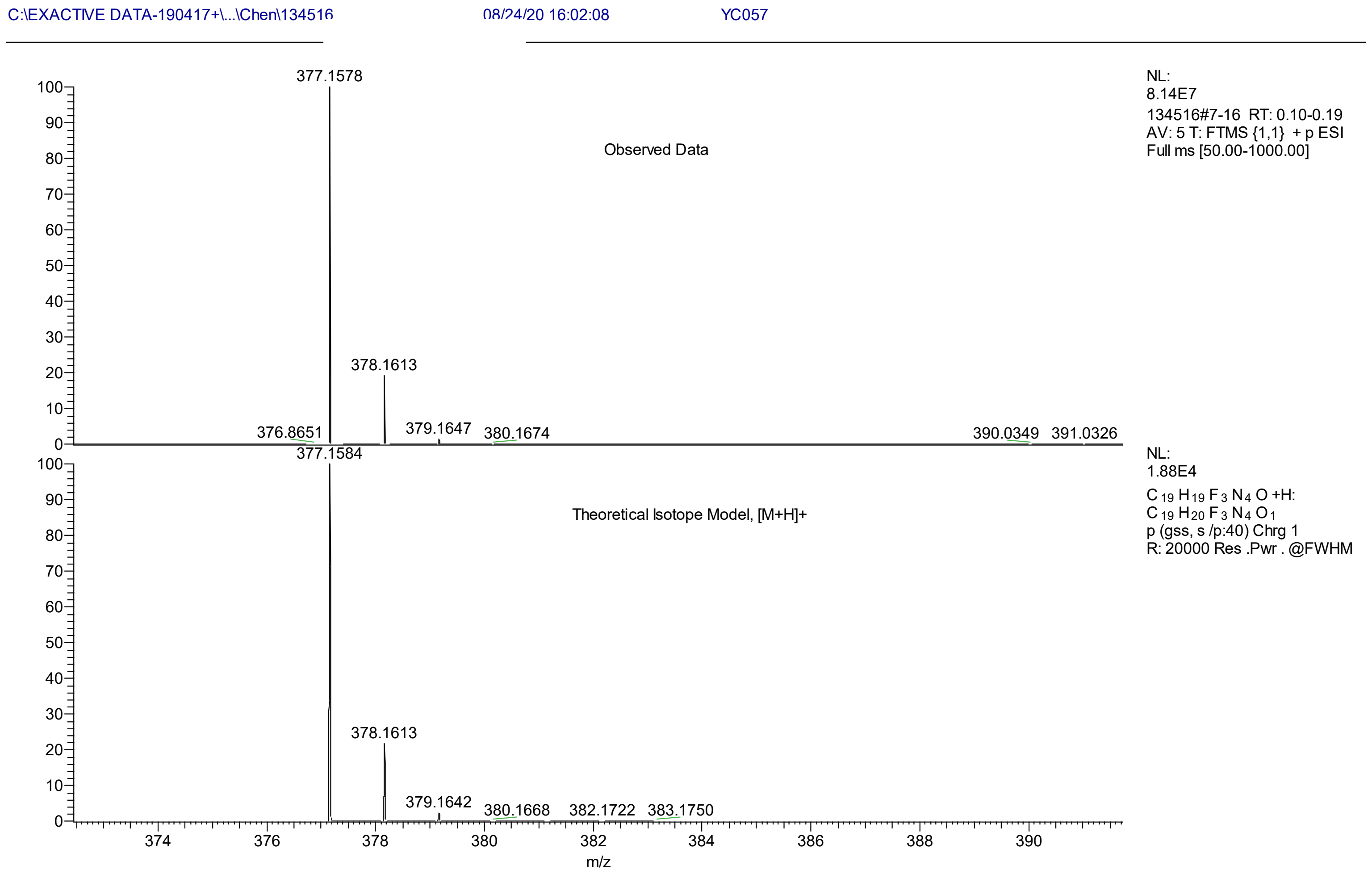

Figure S5-4: HRMS spectrum of Compound **5.**

1-((3-chlorobenzyl)(methyl)amino)-2-(2,4-difluorophenyl)-3-(1H-1,2,4-triazol-1-yl)propan-2-ol **(6)**

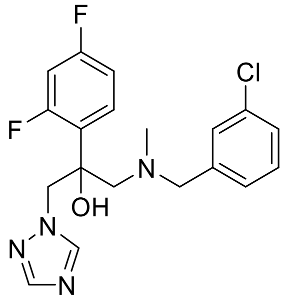

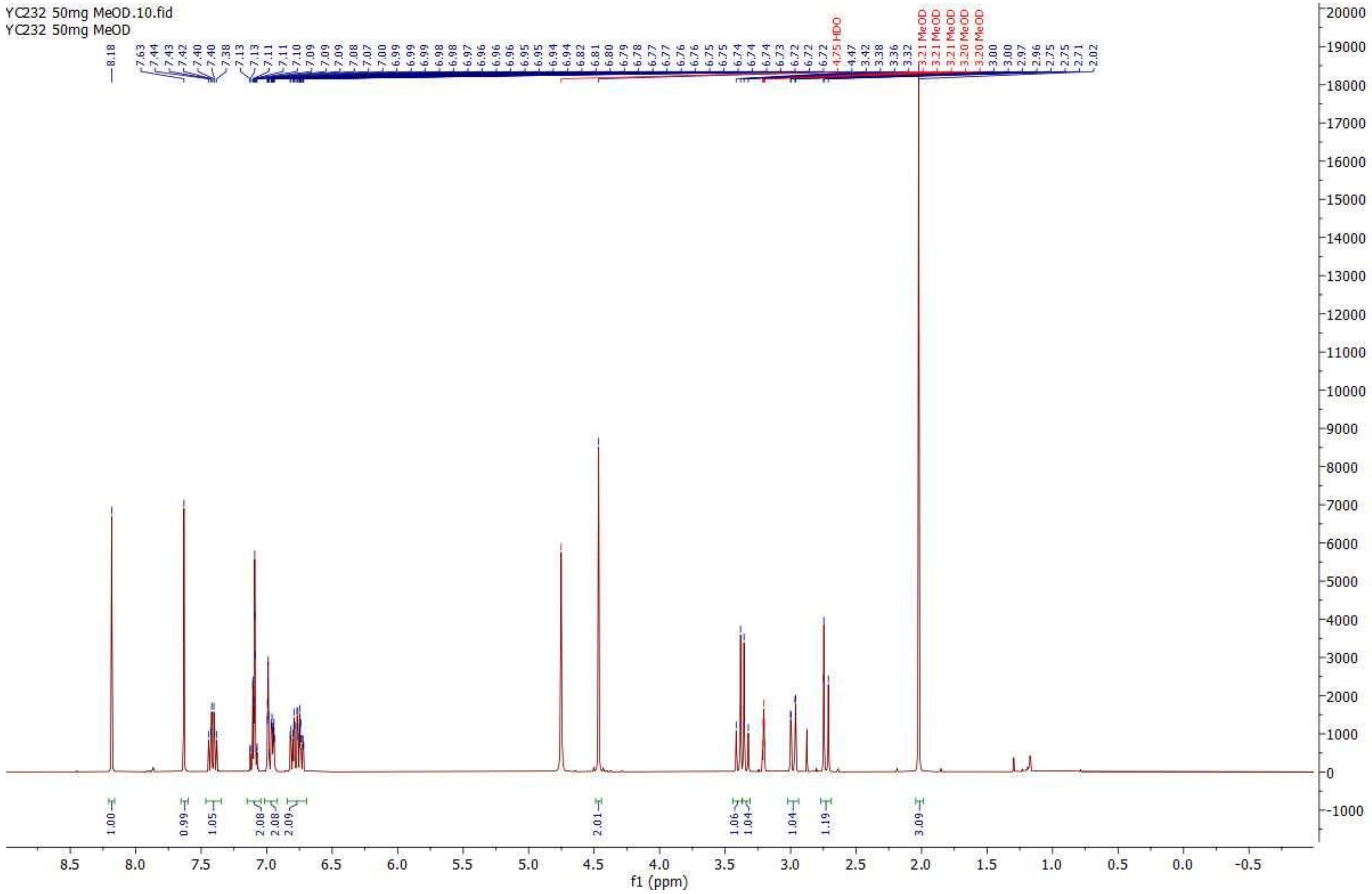

Figure S6-1: Proton ^{1}H NMR spectrum of Compound **6.**

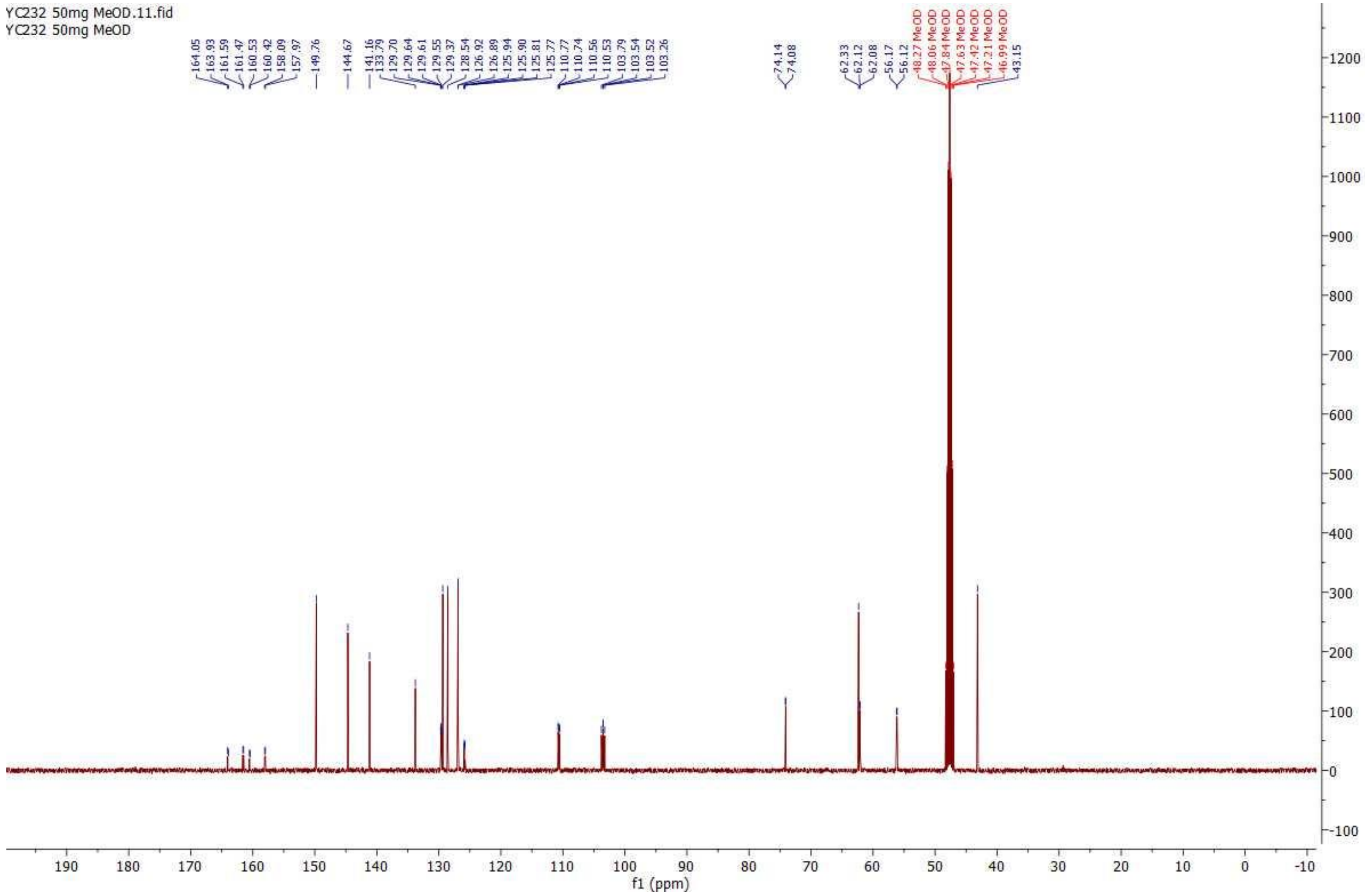

Figure S6-2: Carbon ^{13}C NMR spectrum of Compound **6.**

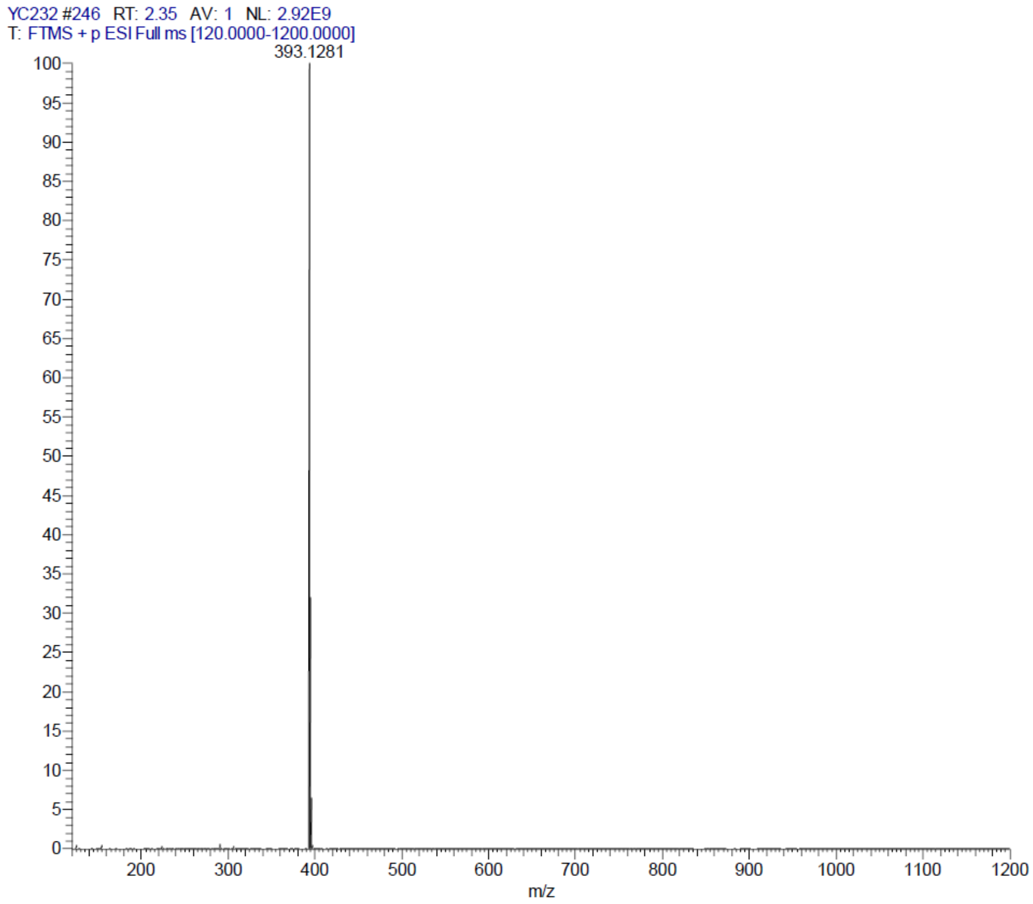

Figure S6-3: HRMS spectrum of Compound **6.**

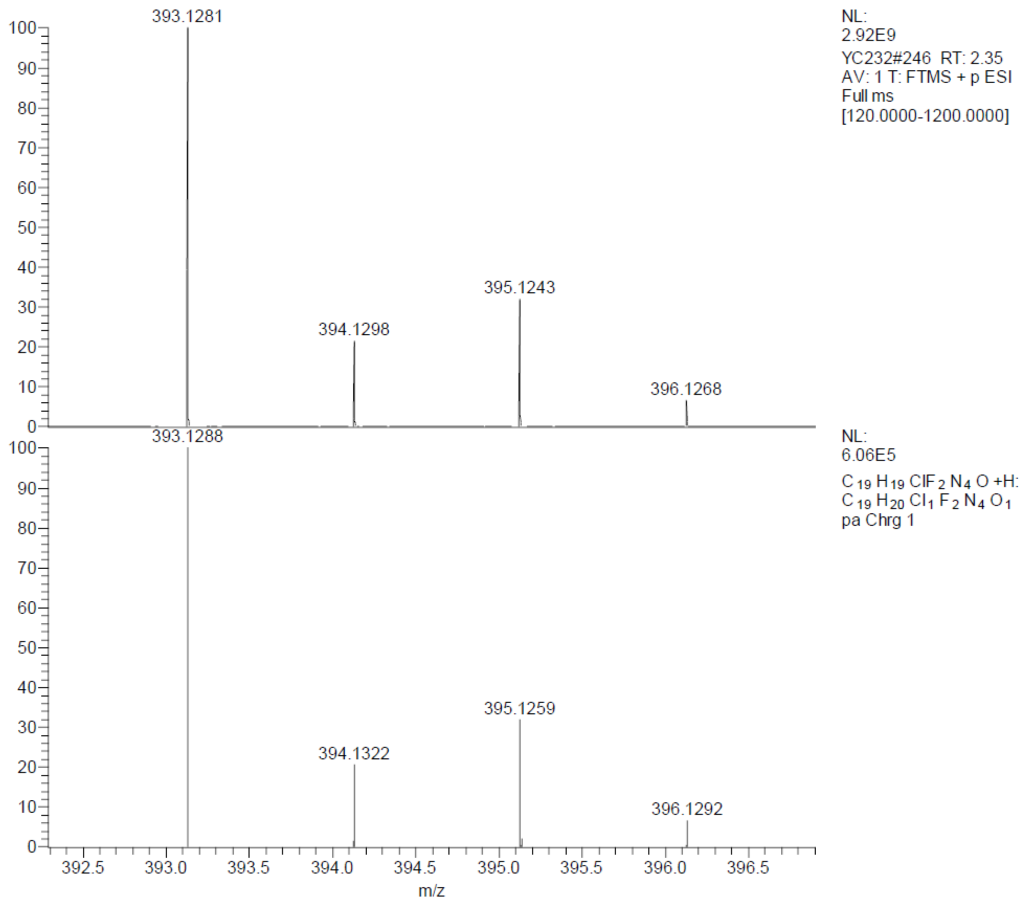

Figure S6-4: HRMS spectrum of Compound **6.**

1-((3-bromobenzyl)(methyl)amino)-2-(2,4-difluorophenyl)-3-(1H-1,2,4-triazol-1-yl)propan-2-ol (7)

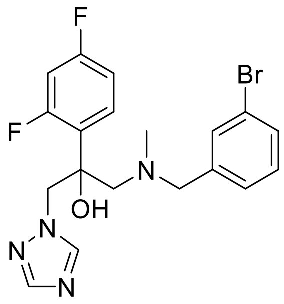

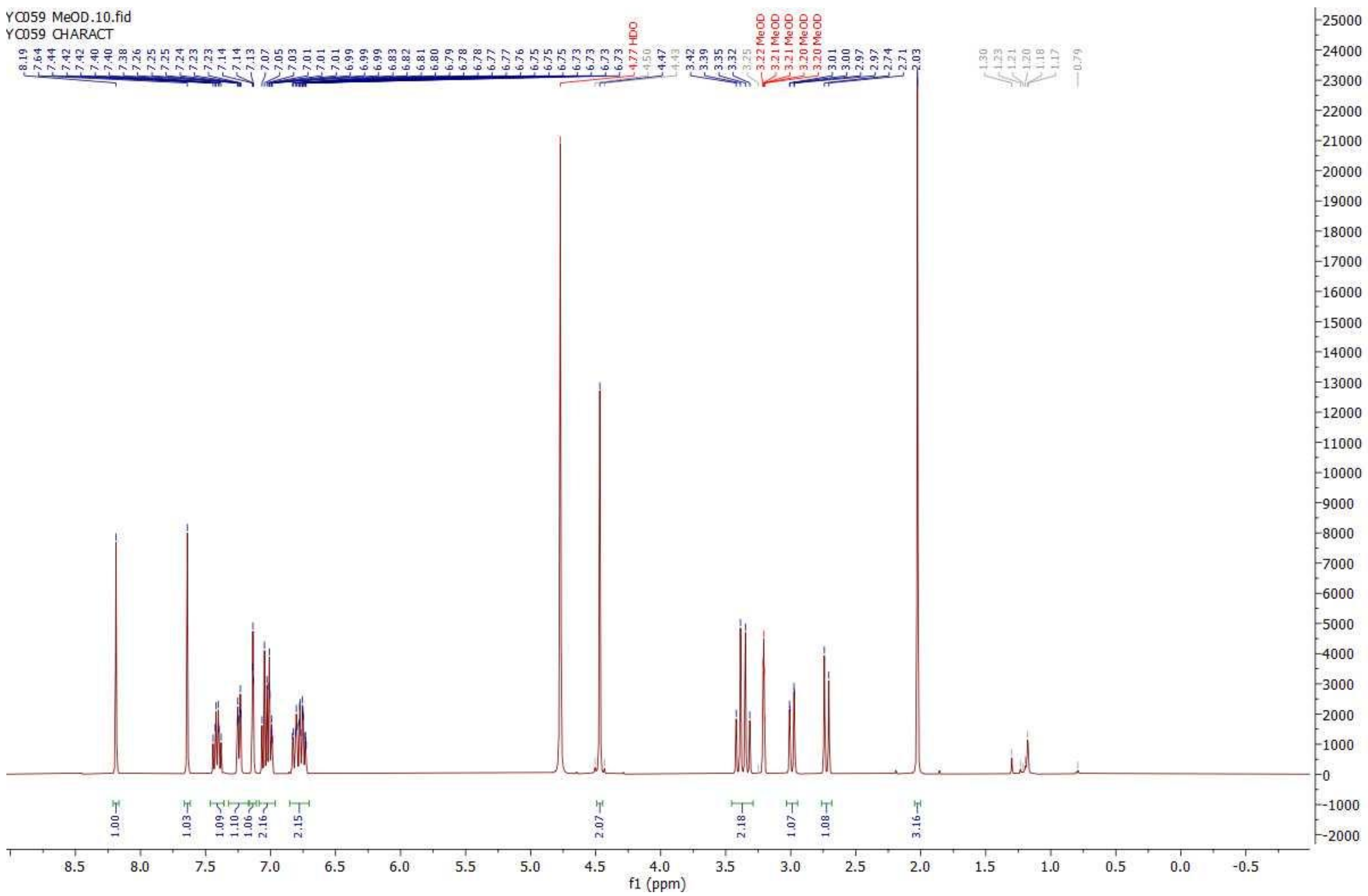

Figure S7-1: Proton ^{1}H NMR spectrum of Compound **7.**

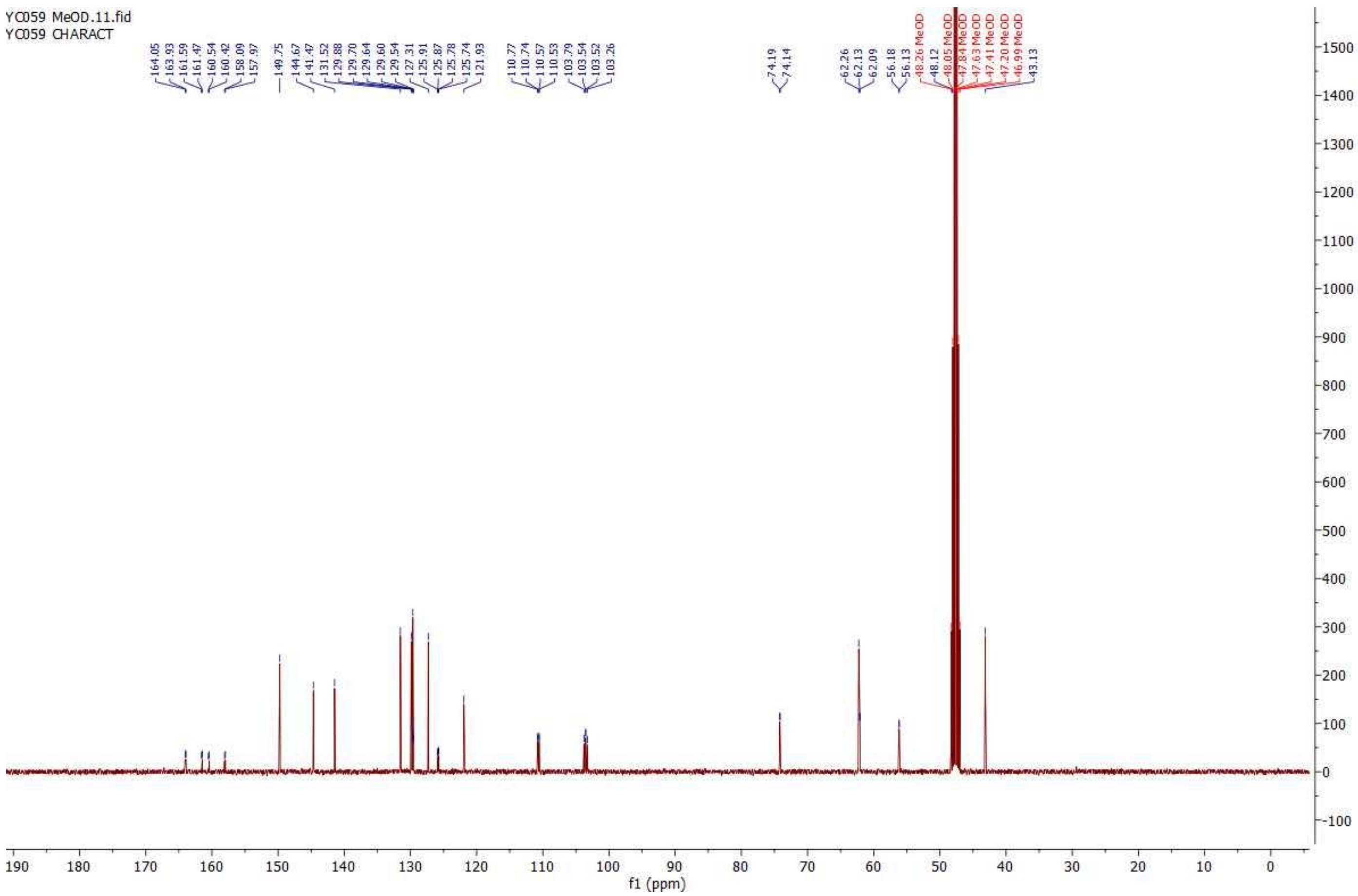

Figure S7-2: Carbon ^{13}C NMR spectrum of Compound **7.**

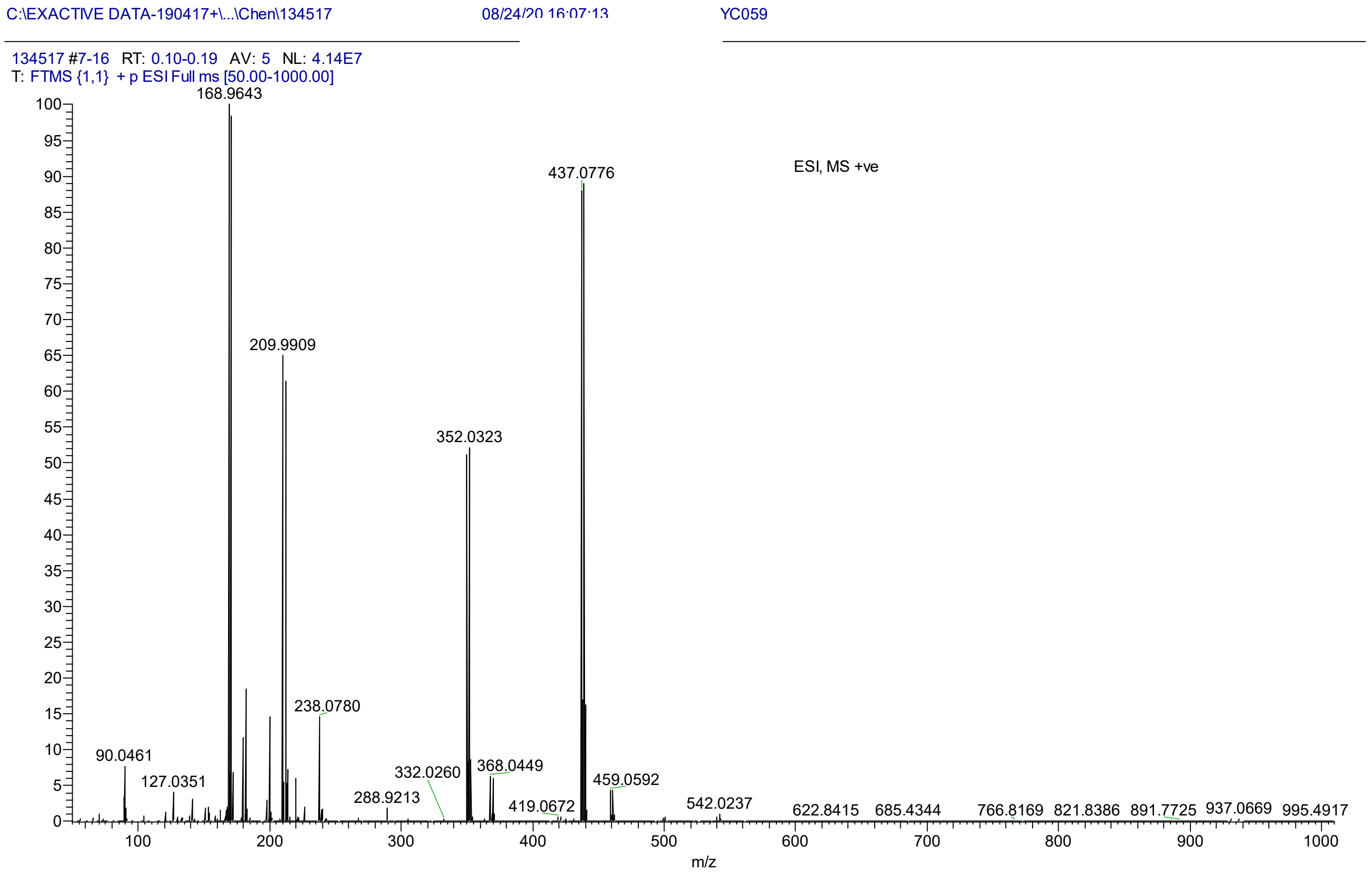

Figure S7-3: HRMS spectrum of Compound **7.**

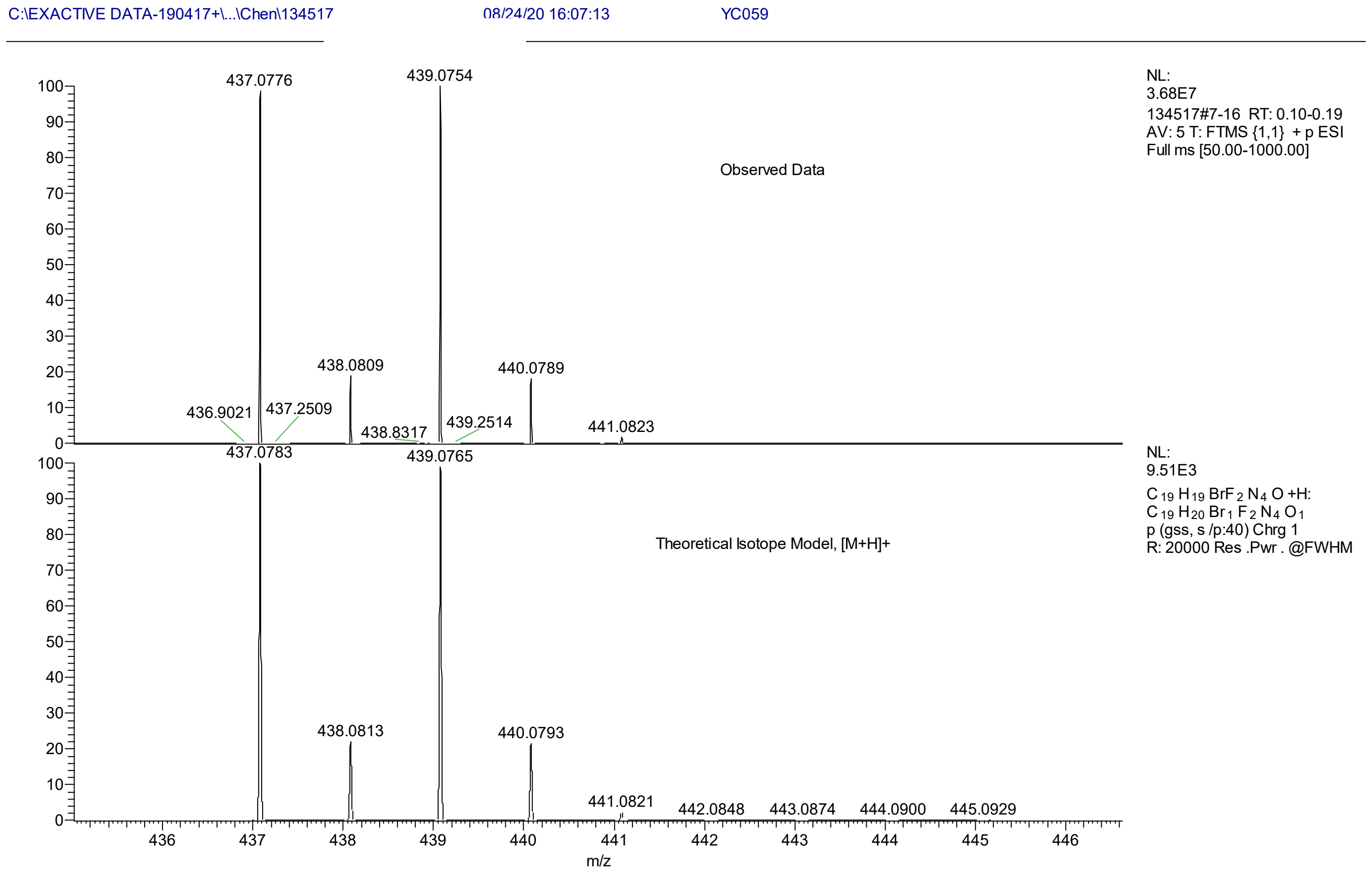

Figure S7-4: HRMS spectrum of Compound **7.**

2-(((2-(2,4-difluorophenyl)-2-hydroxy-3-(1H-1,2,4-triazol-1-yl)propyl)(methyl)amino)methyl)phenol (8)

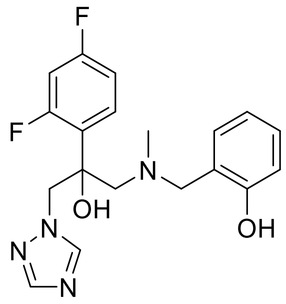

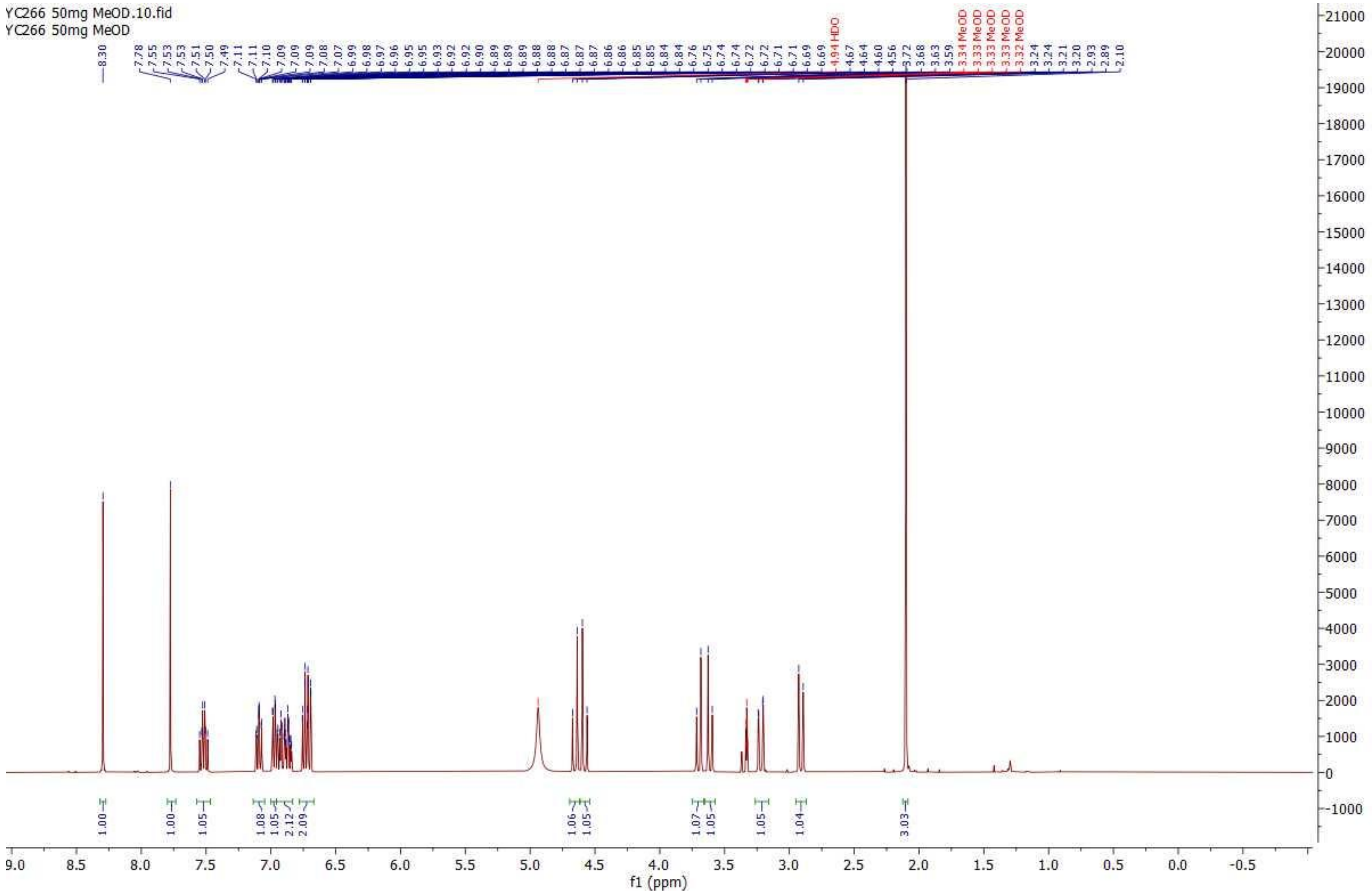

Figure S8-1: Proton ^1H NMR spectrum of Compound **8.**

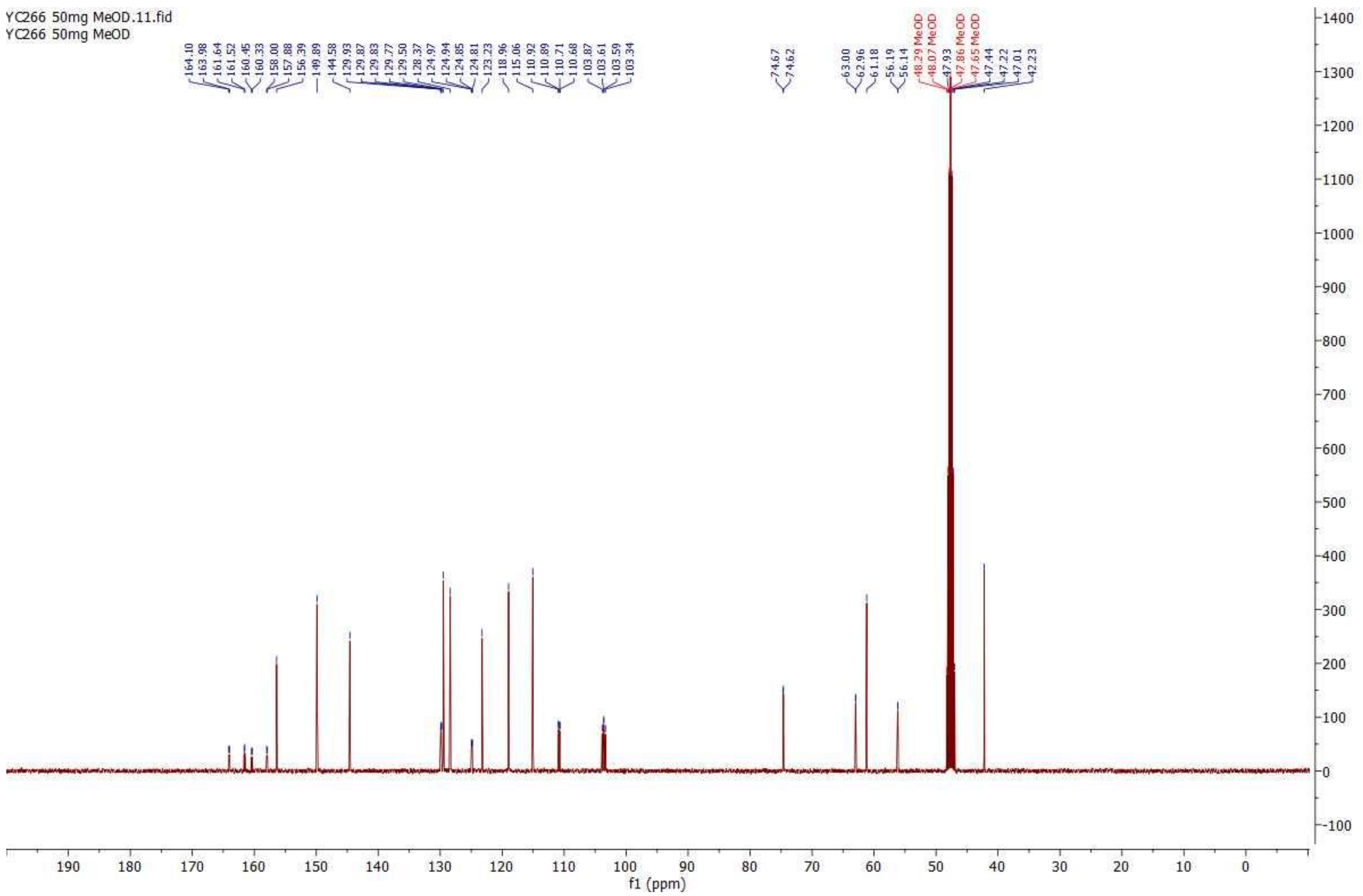

Figure S8-2: Carbon ^{13}C NMR spectrum of Compound **8.**

3-(((2-(2,4-difluorophenyl)-2-hydroxy-3-(1H-1,2,4-triazol-1-yl)propyl)(methyl)amino)methyl)phenol (9)

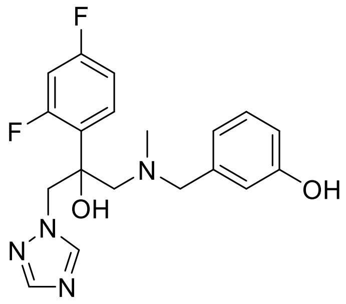

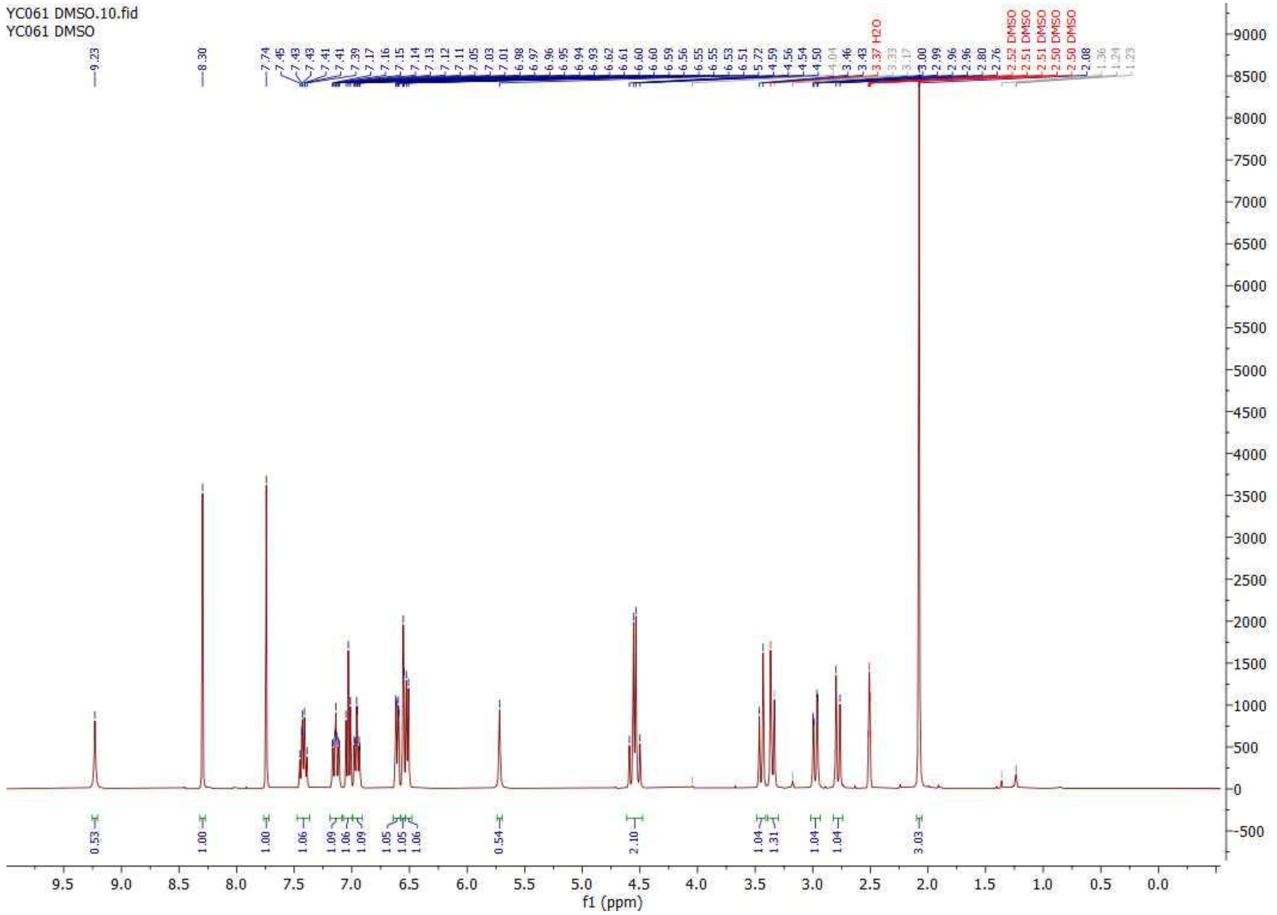

Figure S9-1: Proton ^{1}H NMR spectrum of Compound **9.**

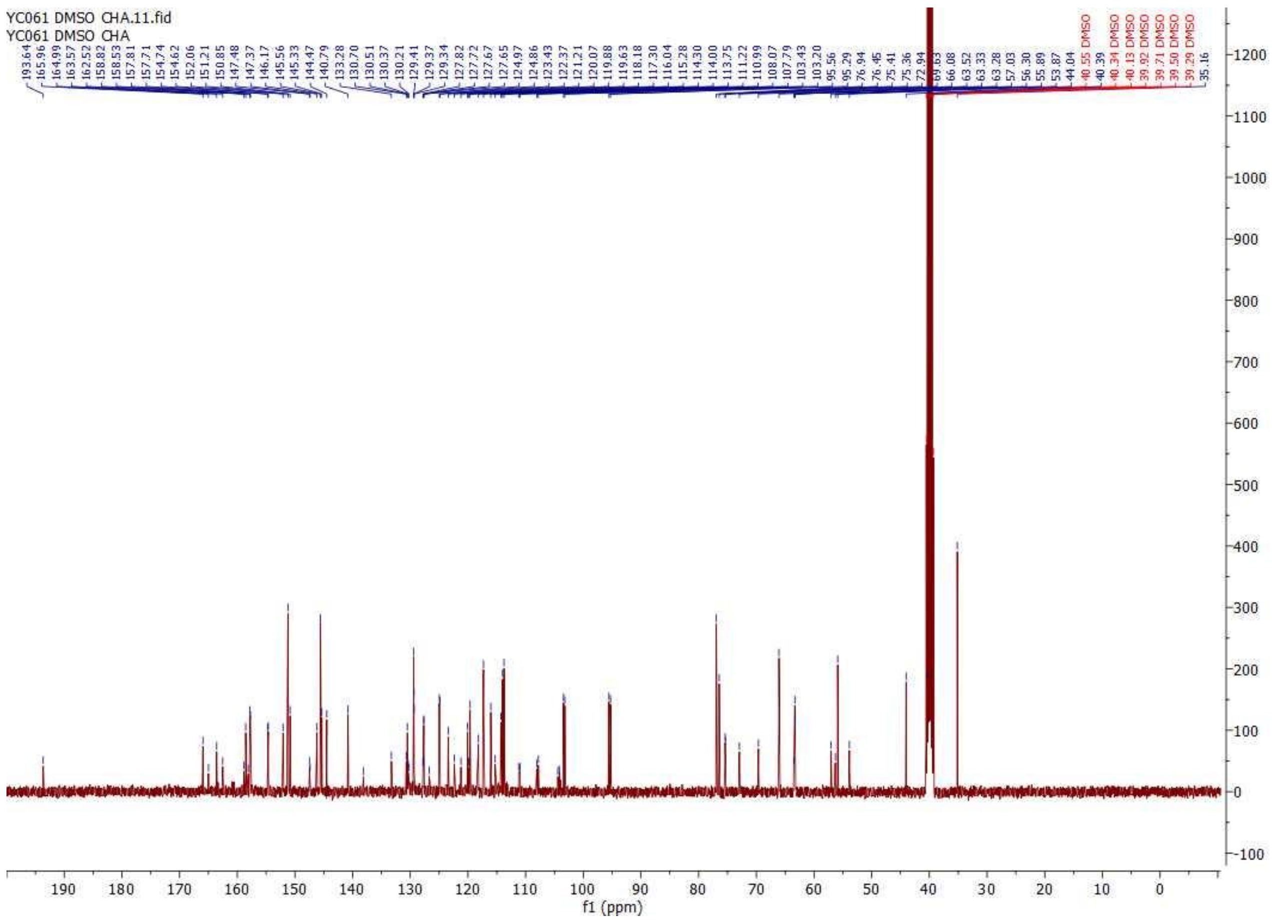

Figure S9-2: Carbon ^{13}C NMR spectrum of Compound **9.**

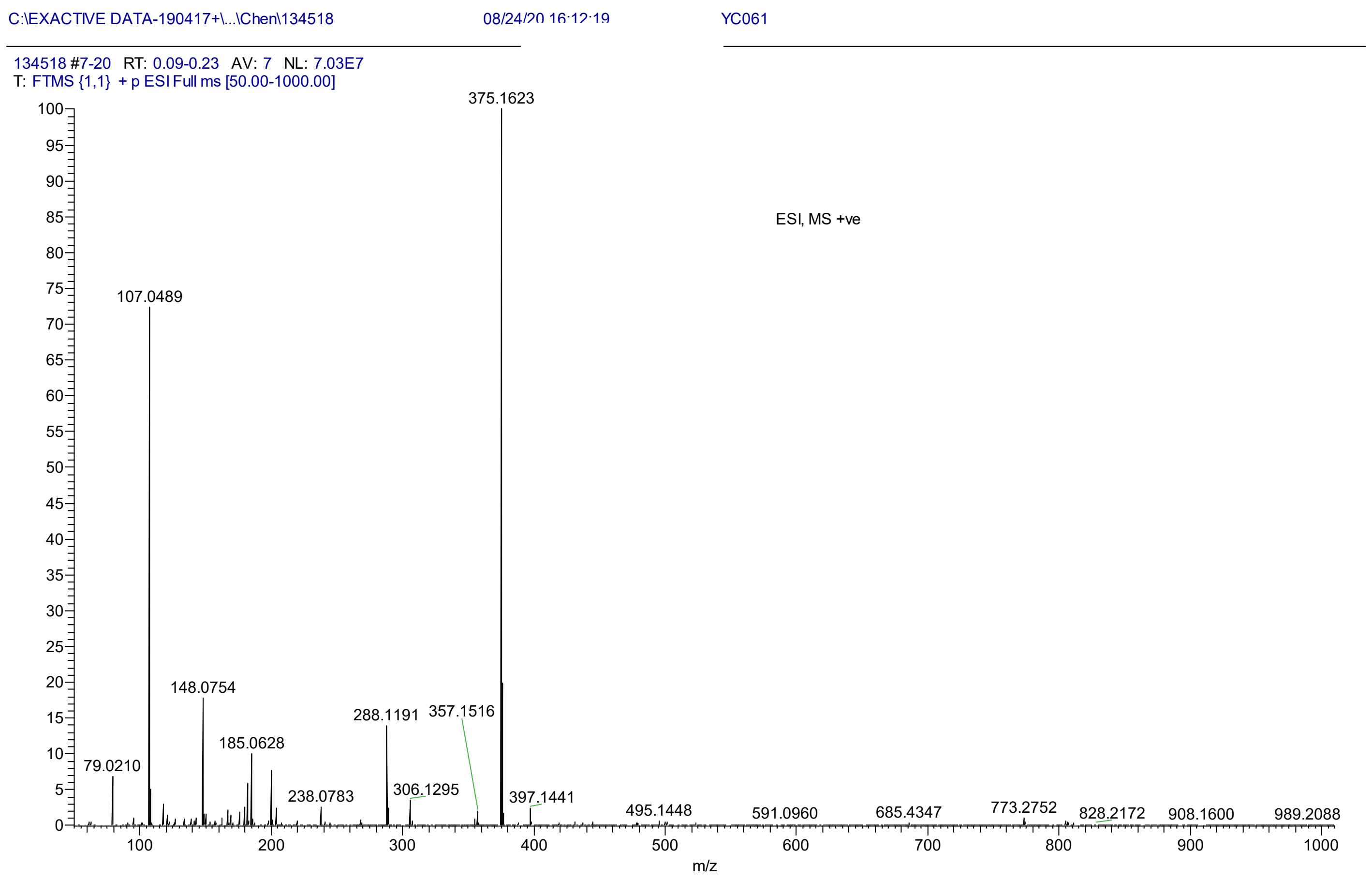

Figure S9-3: HRMS spectrum of Compound **9.**

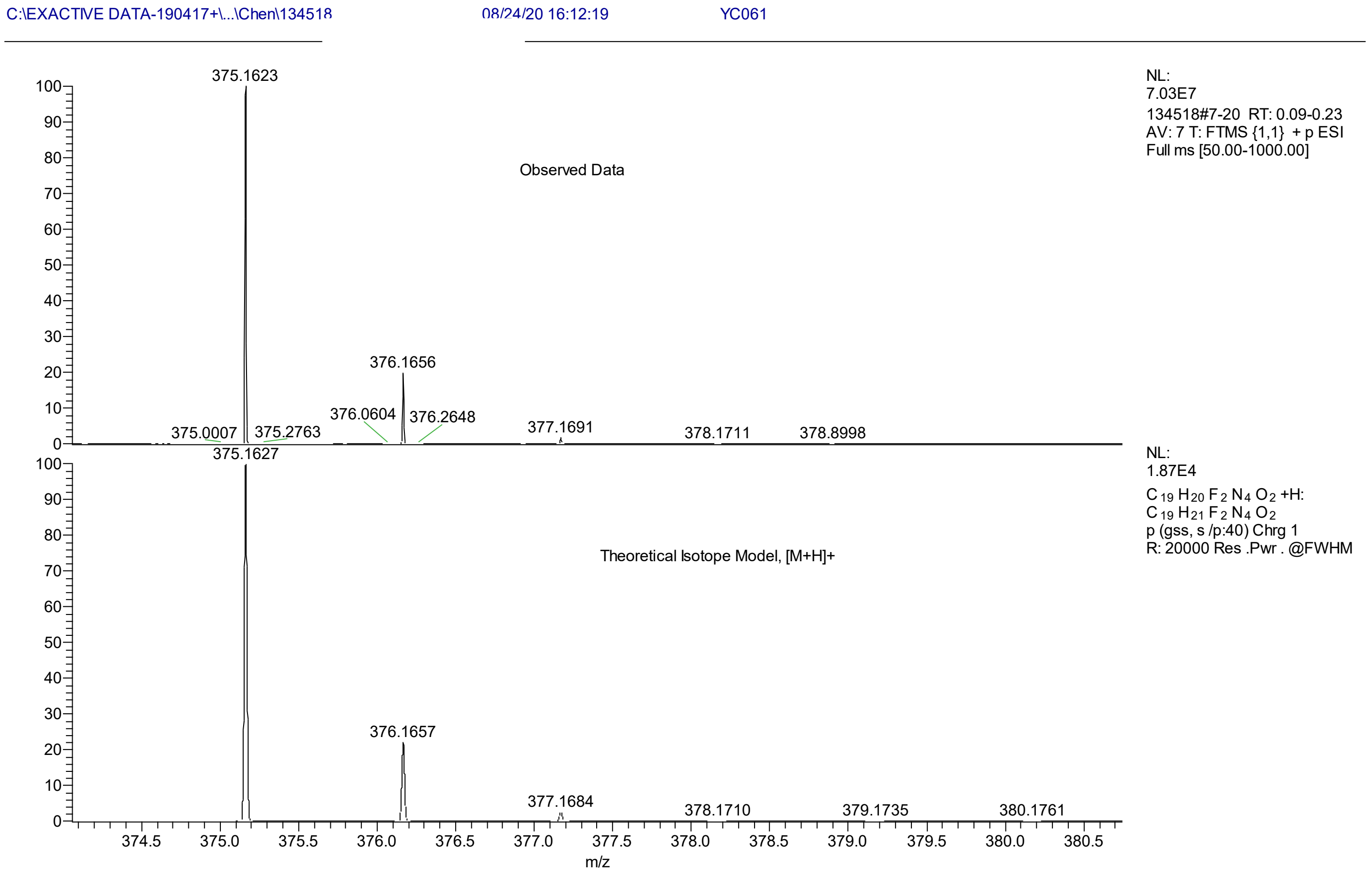

Figure S9-4: HRMS spectrum of Compound **9.**

2-(((2-(2,4-difluorophenyl)-2-hydroxy-3-(1H-1,2,4-triazol-1-yl)propyl)(methyl)amino)methyl)-4-fluorophenol (10)

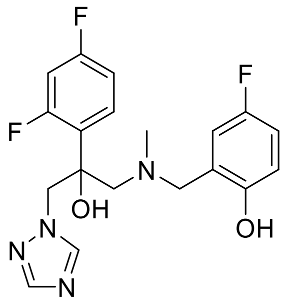

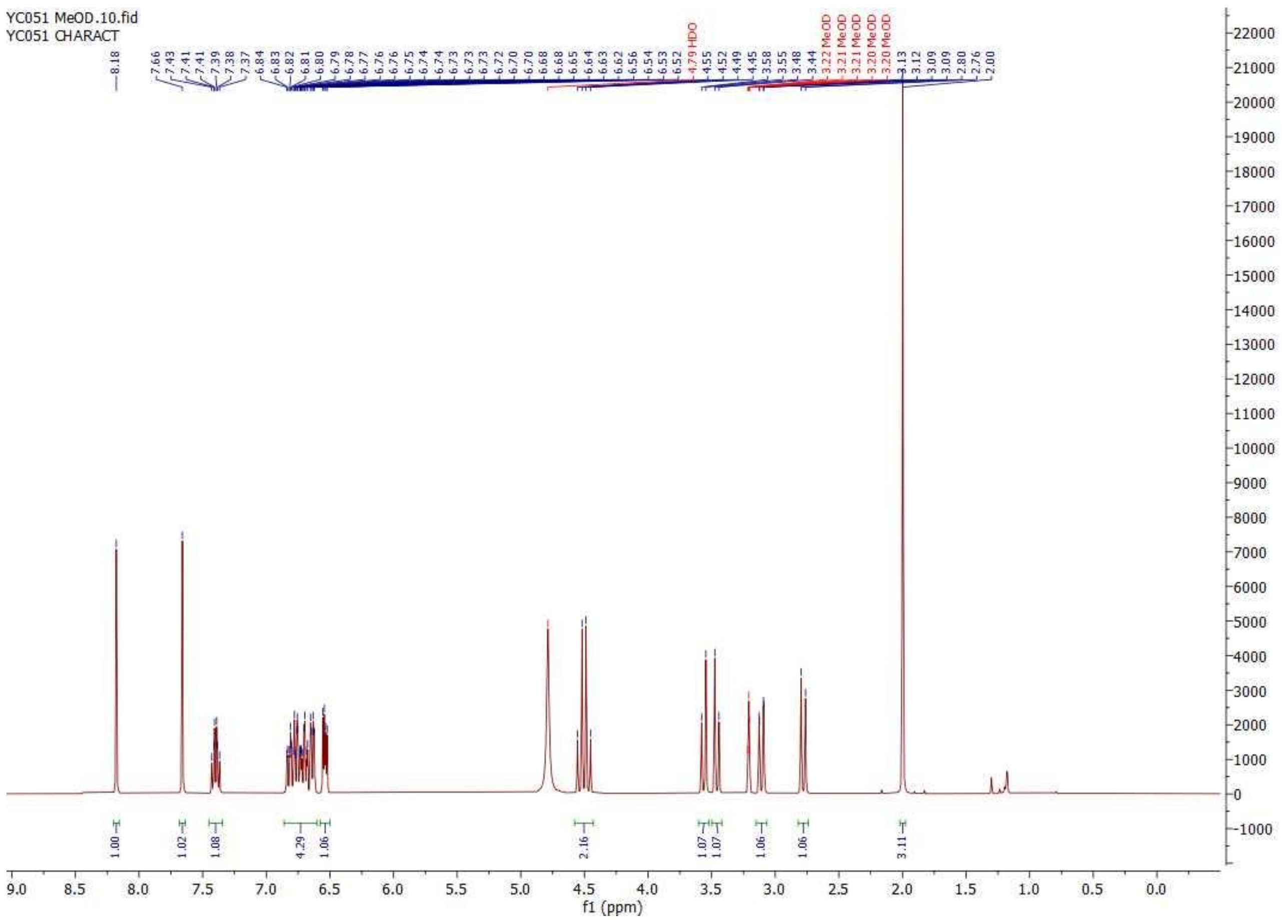

Figure S10-1: Proton ^{1}H NMR spectrum of Compound **10.**

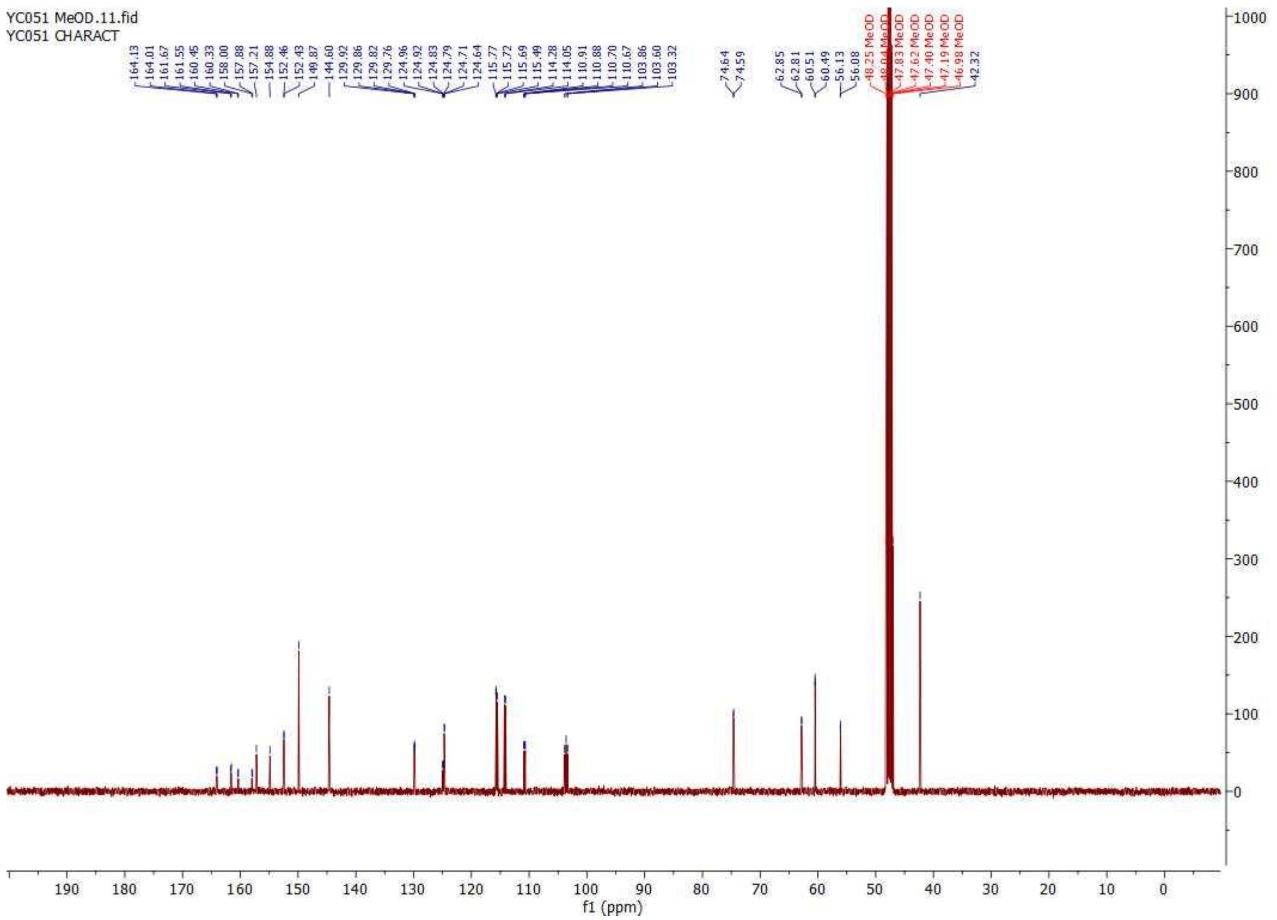

Figure S10-2: Carbon ^{13}C NMR spectrum of Compound **10.**

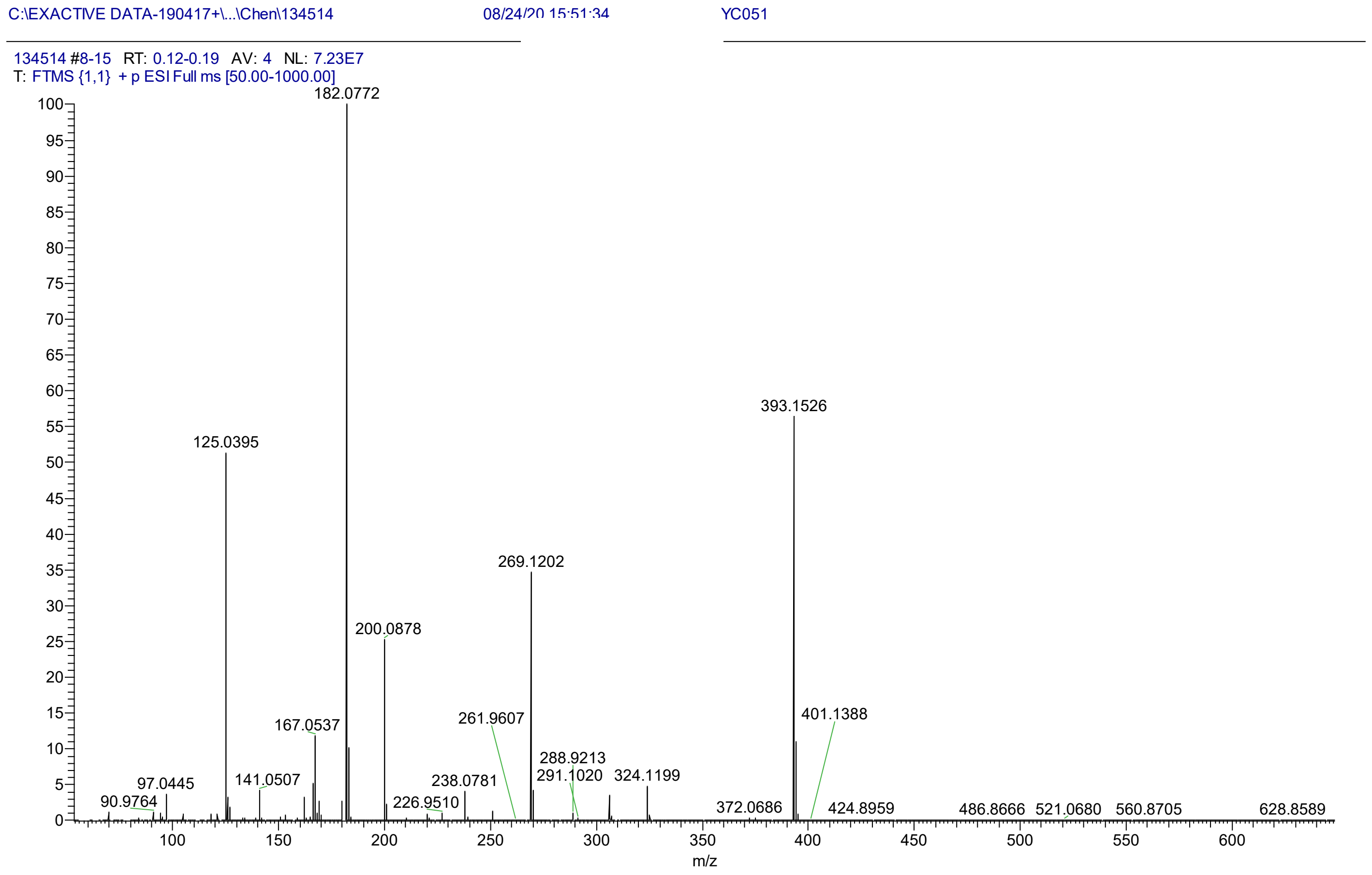

Figure S10-3: HRMS spectrum of Compound **10.**

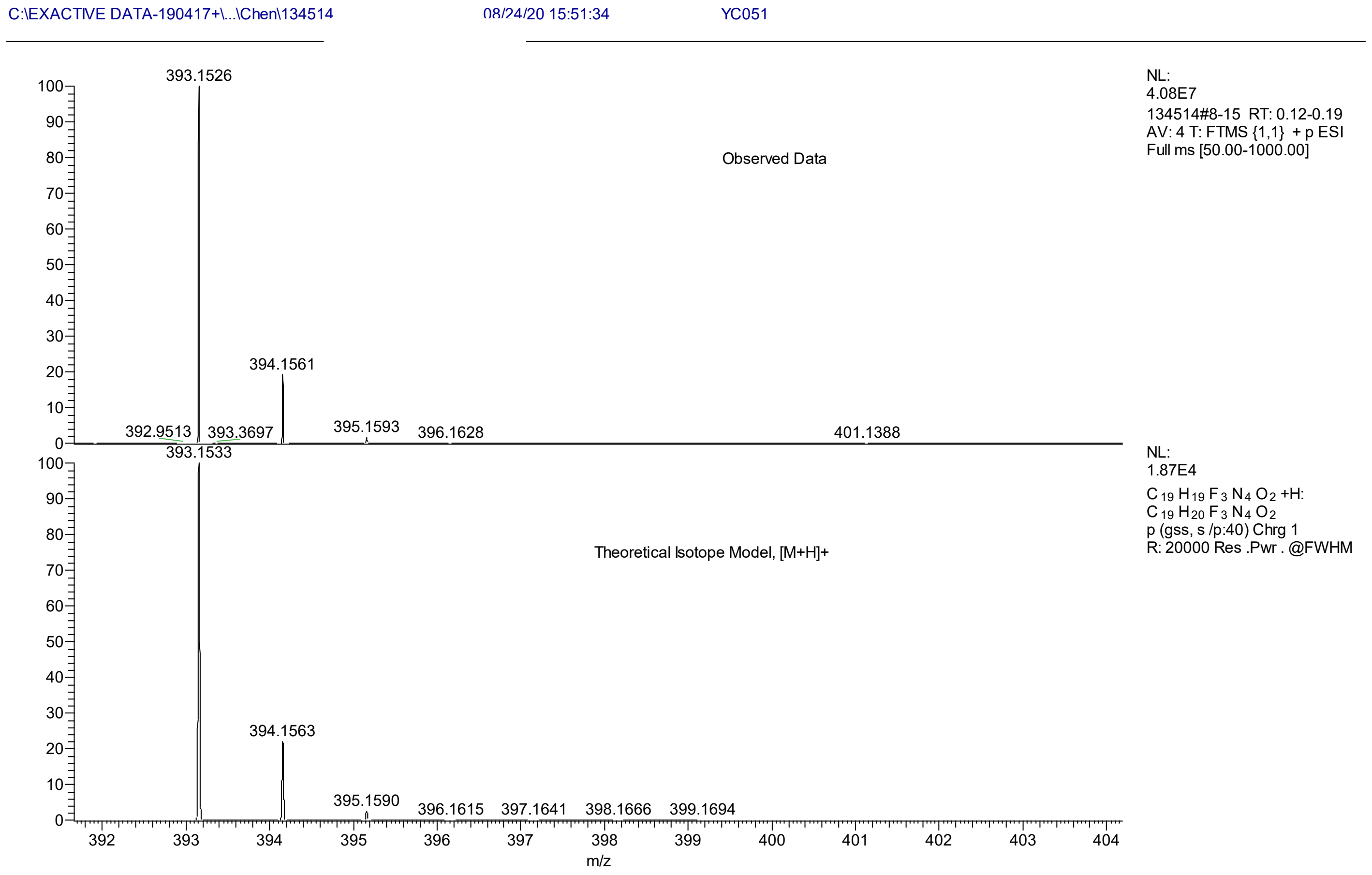

Figure S10-4: HRMS spectrum of Compound **10.**

4-chloro-2-(((2-(2,4-difluorophenyl)-2-hydroxy-3-(1H-1,2,4-triazol-1-yl)propyl)(methyl)amino)methyl)phenol (11)

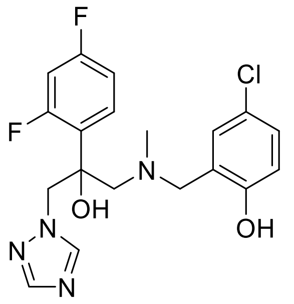

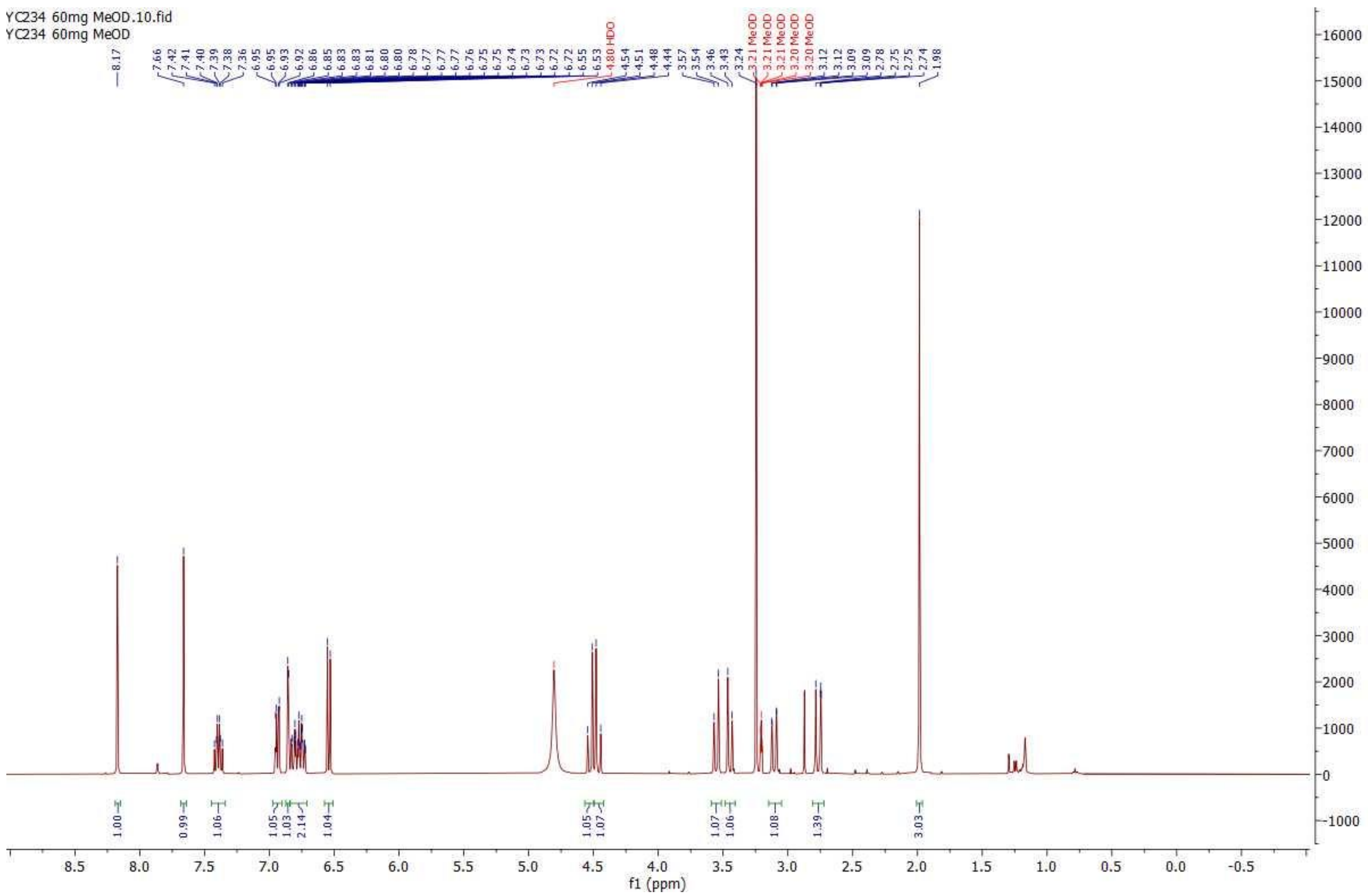

Figure S11-1: Proton ^{1}H NMR spectrum of Compound **11.**

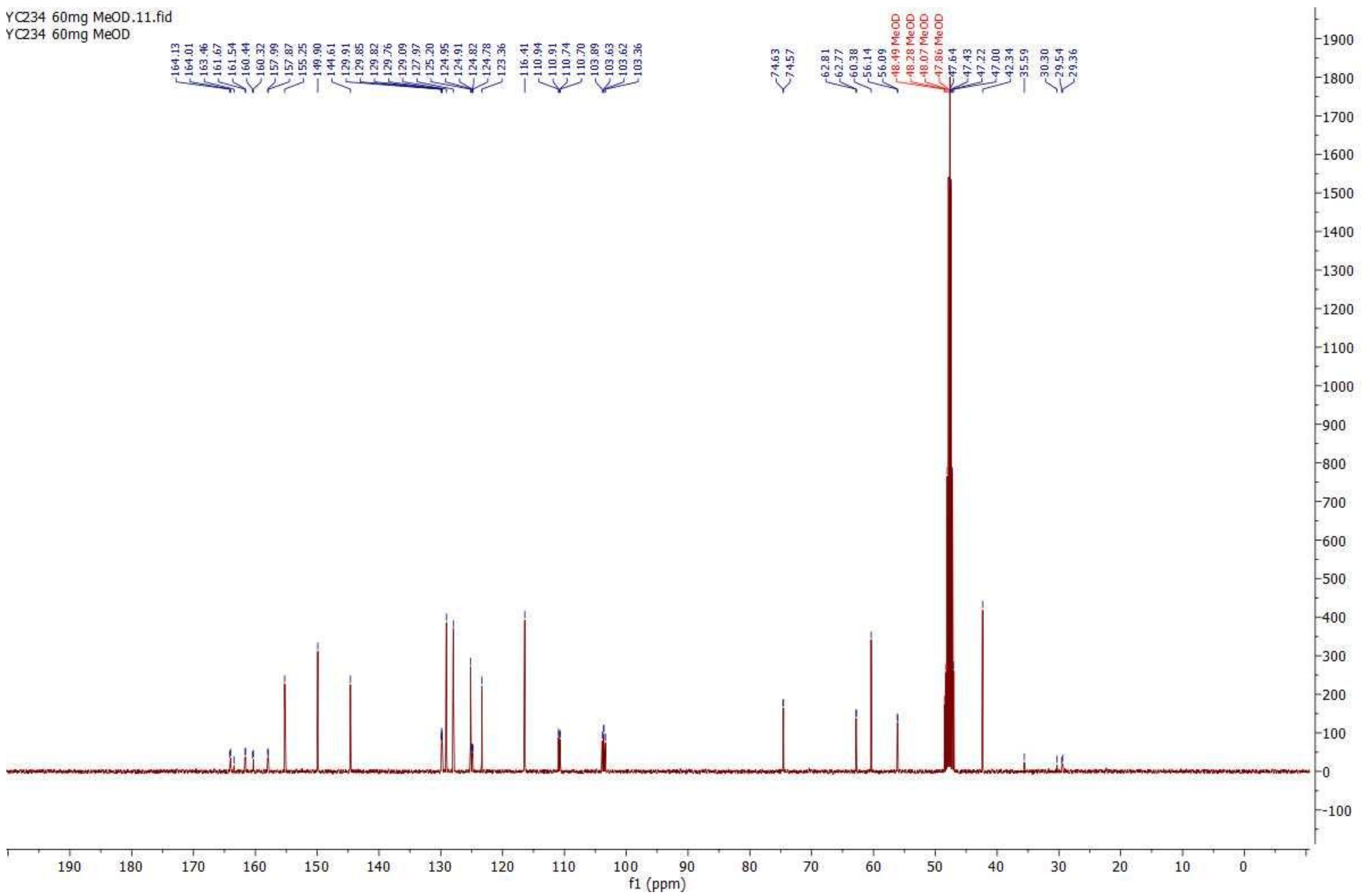

Figure S11-2: Carbon ^{13}C NMR spectrum of Compound **11.**

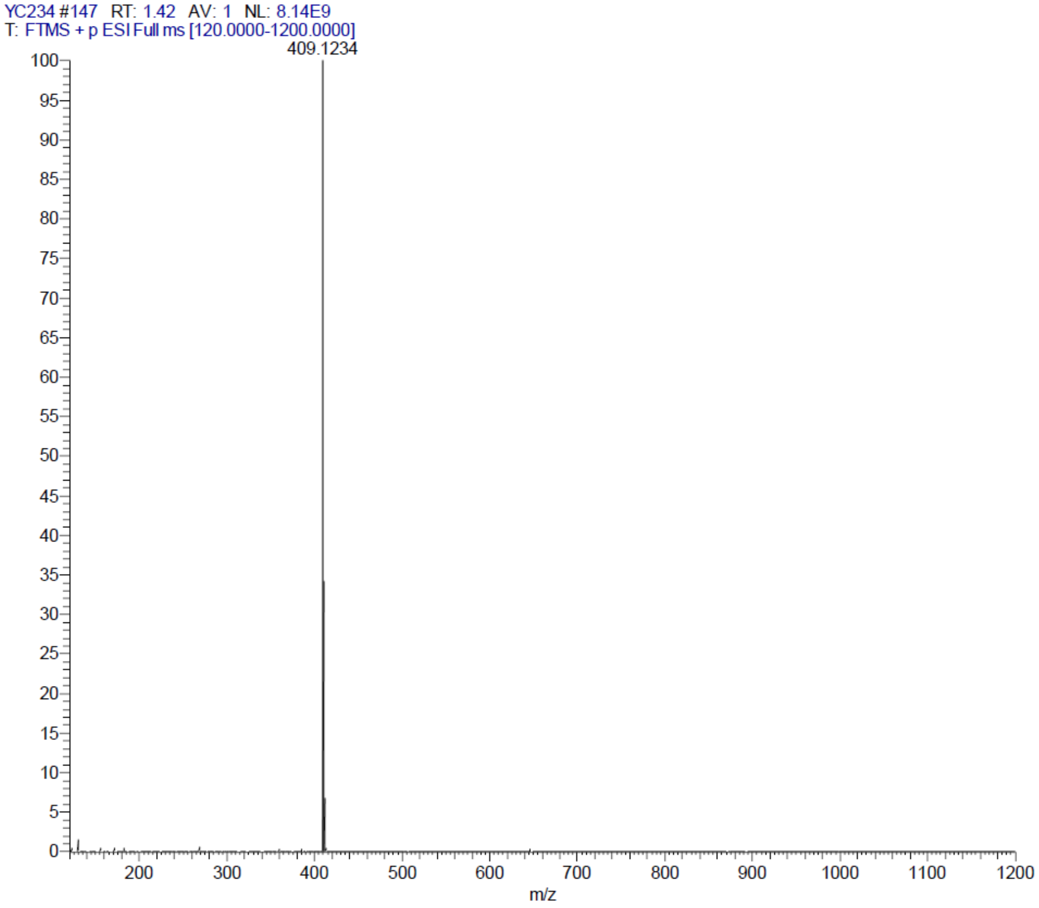

Figure S11-3: HRMS spectrum of Compound **11.**

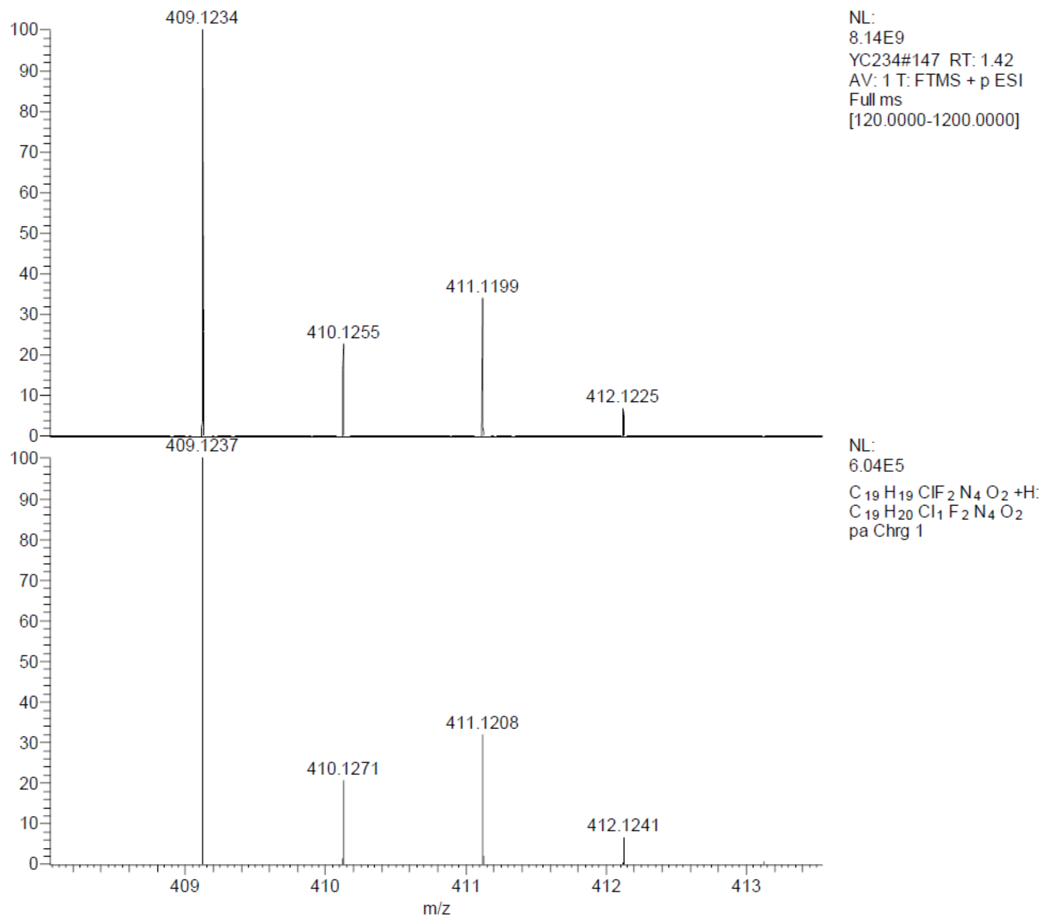

Figure S11-4: HRMS spectrum of Compound **11.**

4-bromo-2-(((2-(2,4-difluorophenyl)-2-hydroxy-3-(1H-1,2,4-triazol-1-yl)propyl)(methyl)amino)methyl)phenol (12)

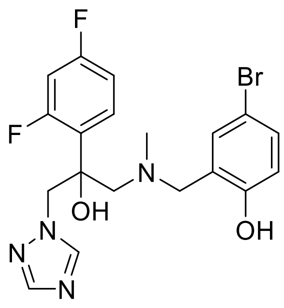

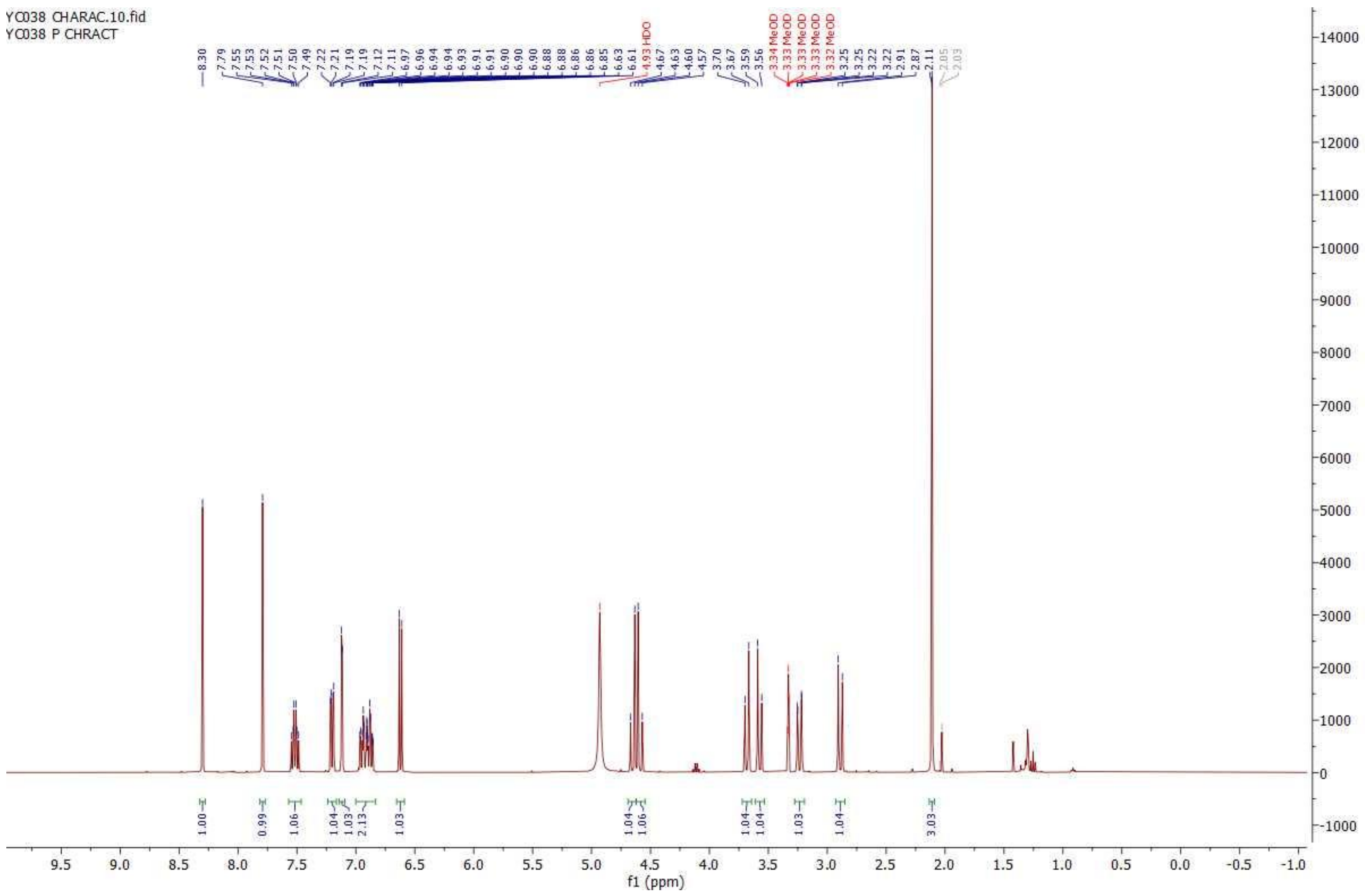

Figure S12-1: Proton ^{1}H NMR spectrum of Compound **12.**

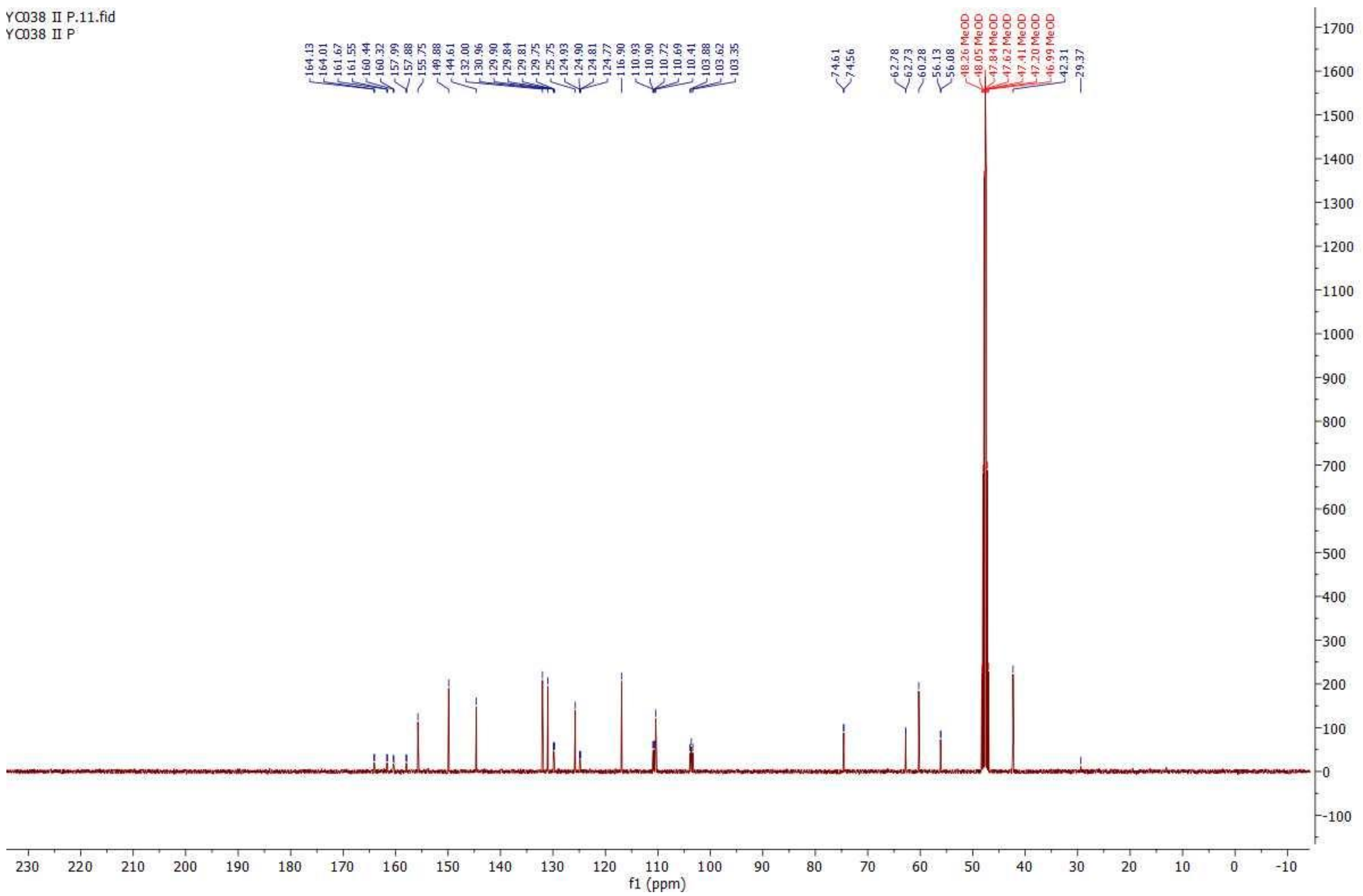

Figure S12-2: Carbon ^{13}C NMR spectrum of Compound **12.**

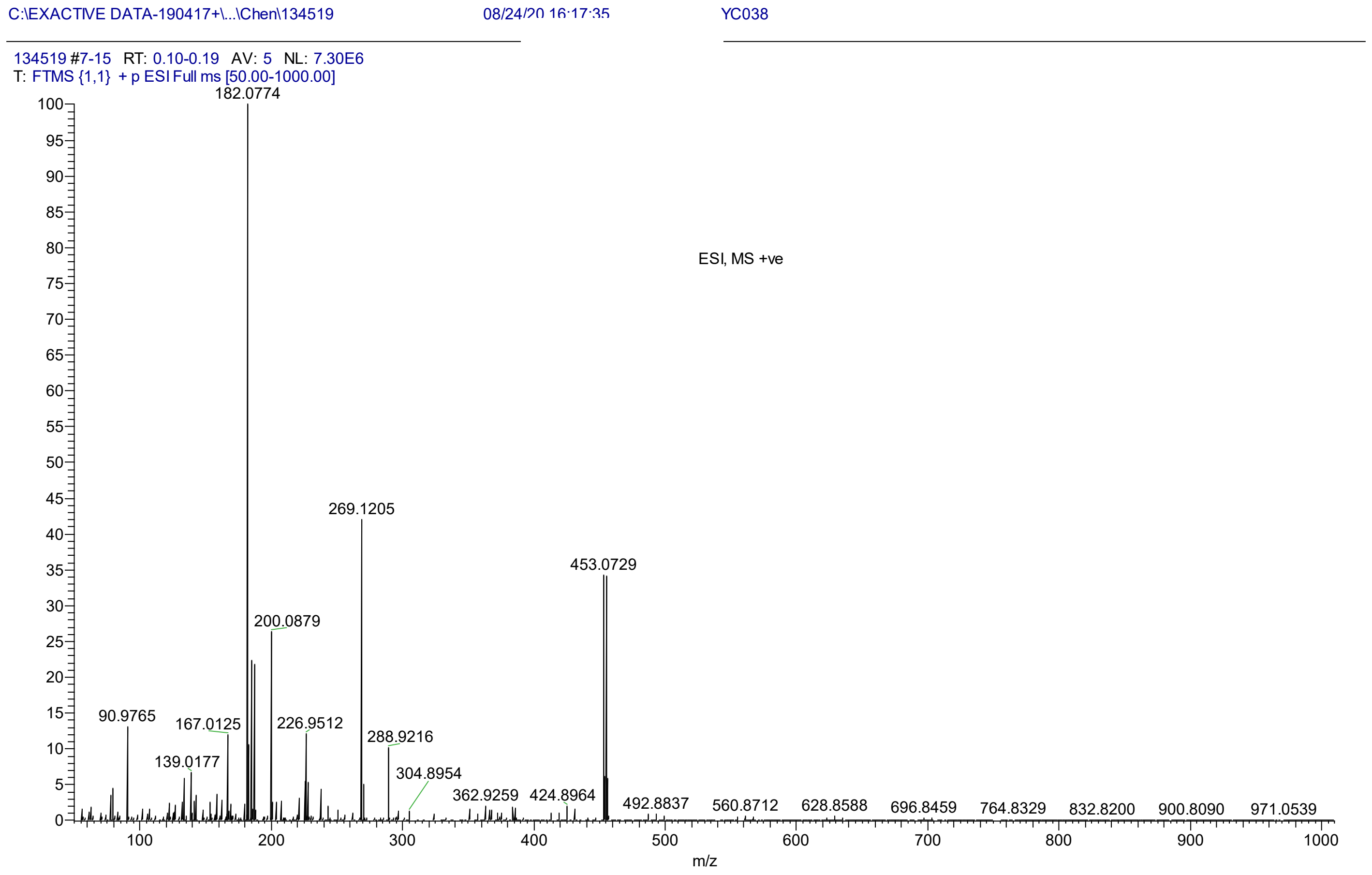

Figure S12-3: HRMS spectrum of Compound **12.**

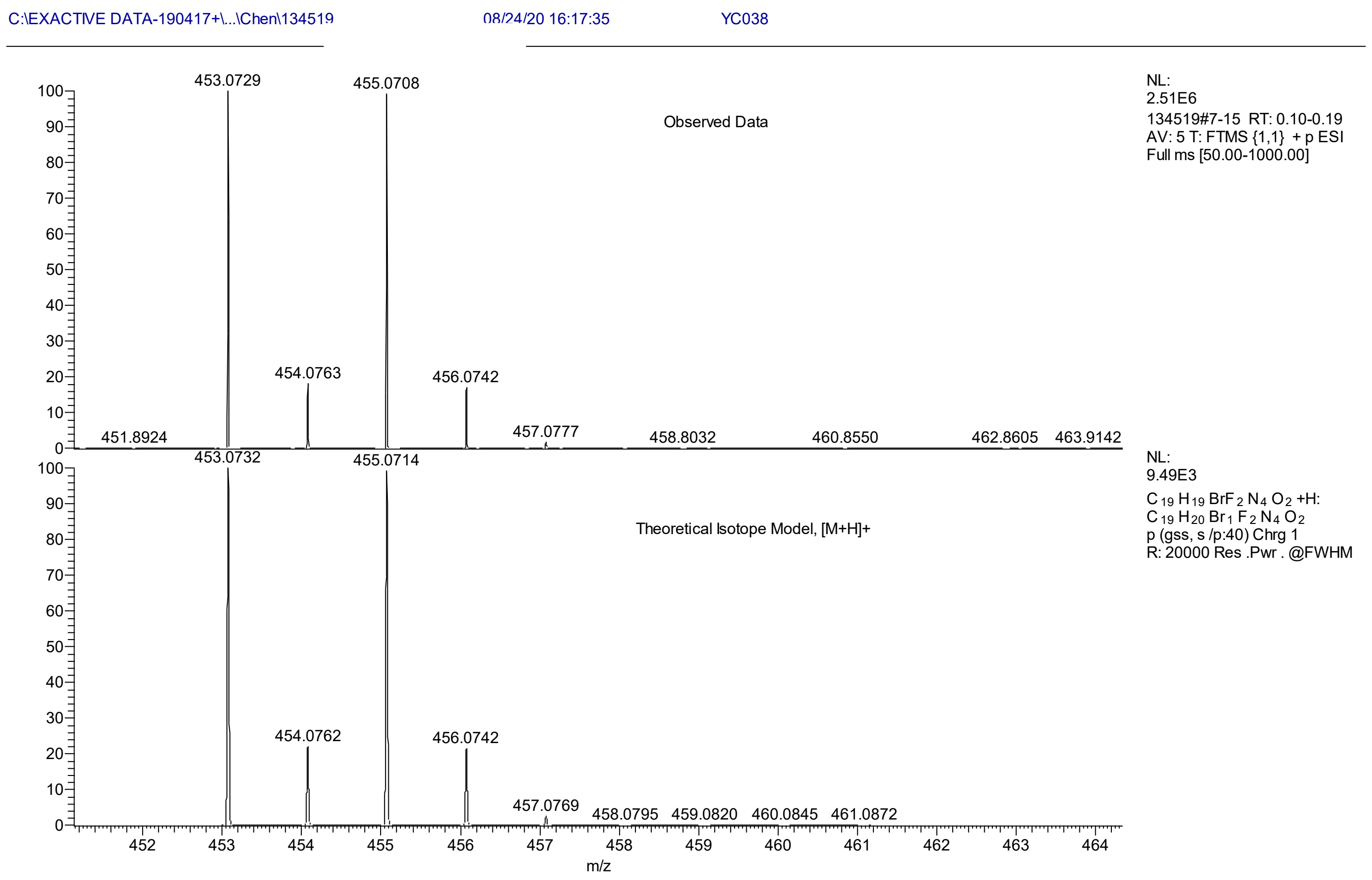

Figure S12-4: HRMS spectrum of Compound **12.**

3-(((2-(2,4-difluorophenyl)-2-hydroxy-3-(1H-1,2,4-triazol-1-yl)propyl)(methyl)amino)methyl)-5-fluorophenol (13)

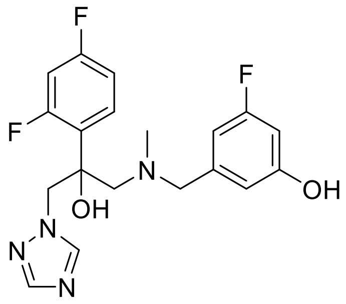

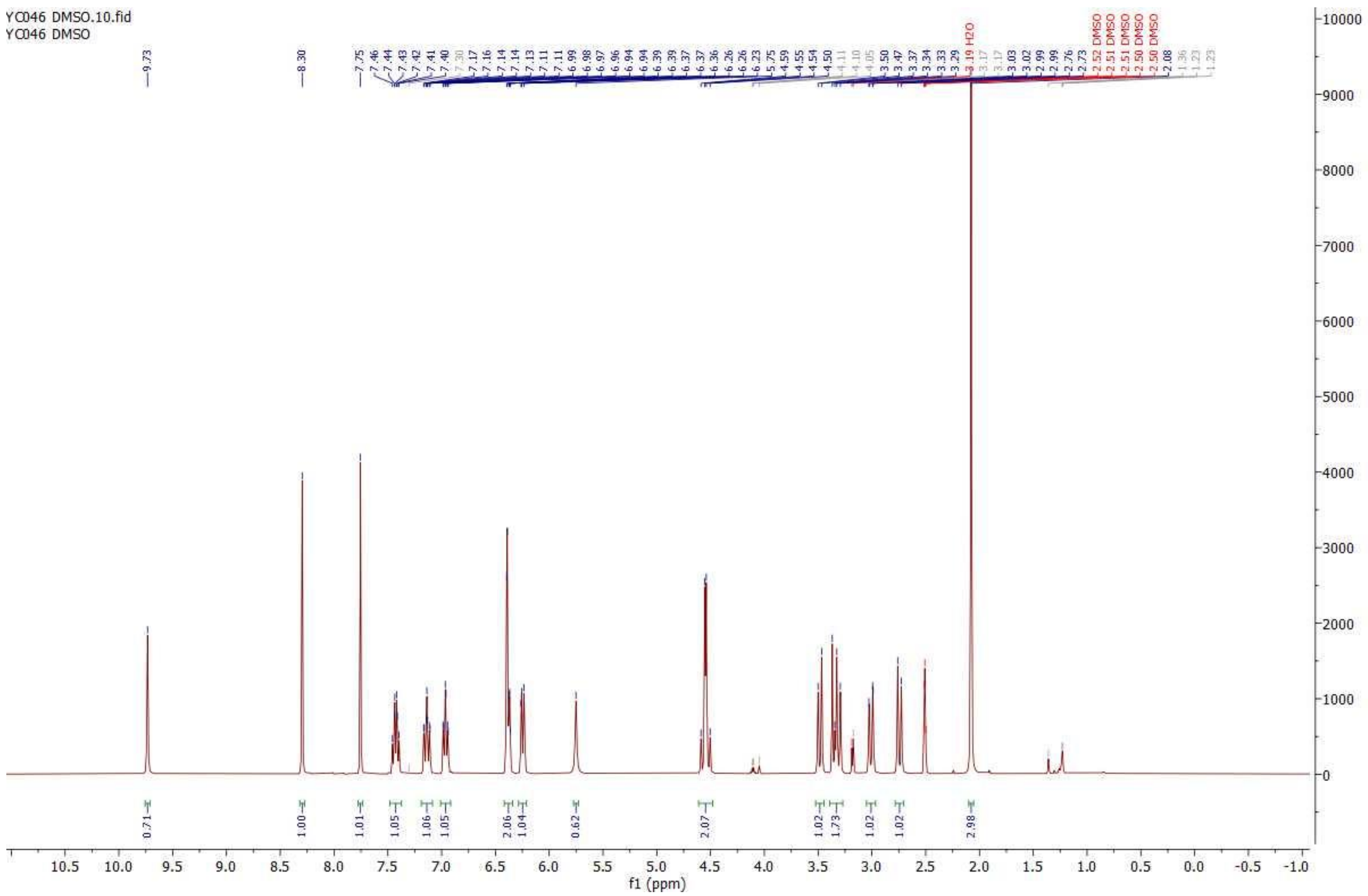

Figure S13-1: Proton ^{1}H NMR spectrum of Compound **13.**

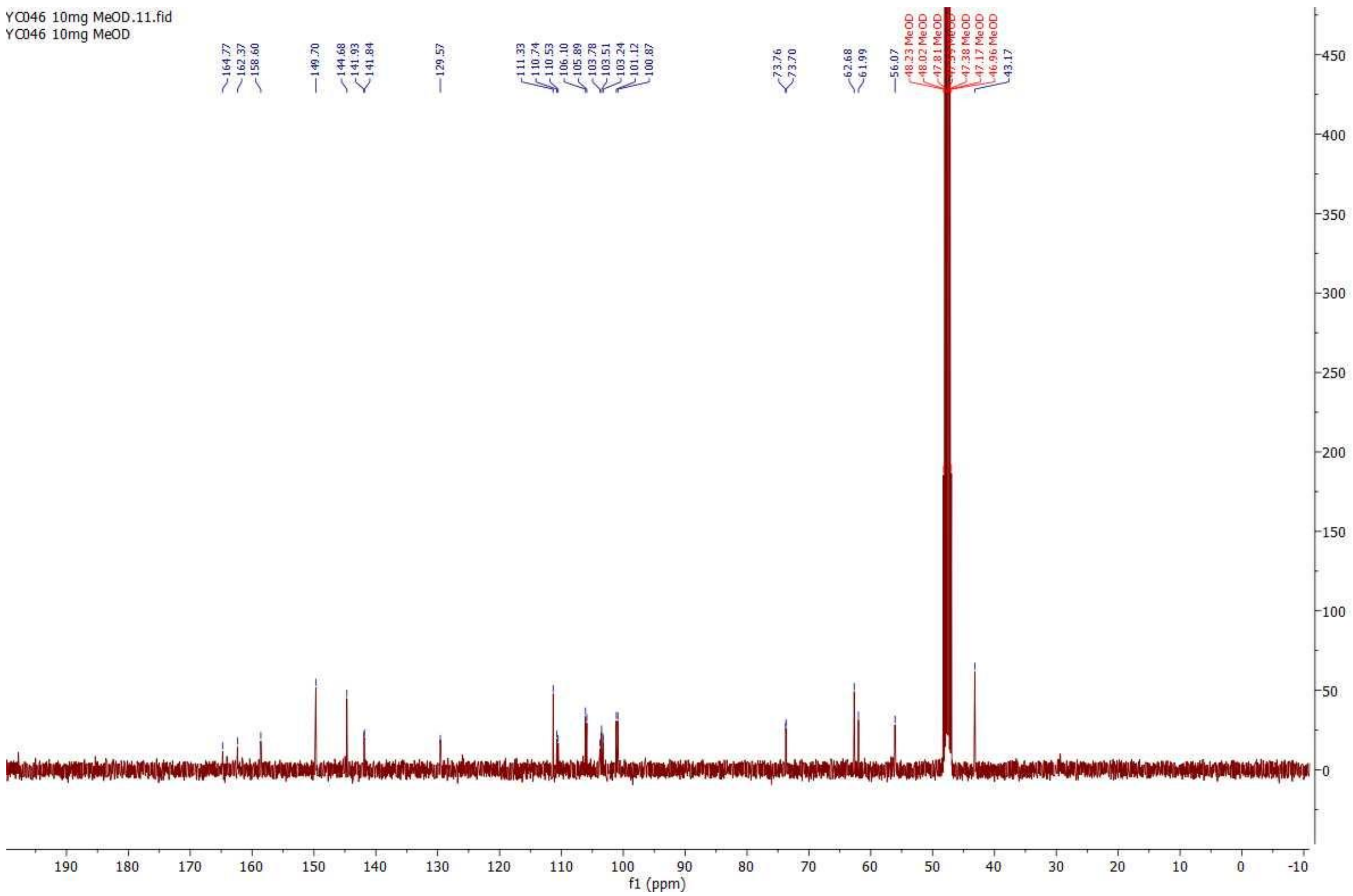

Figure S13-2: Carbon ^{13}C NMR spectrum of Compound **13.**

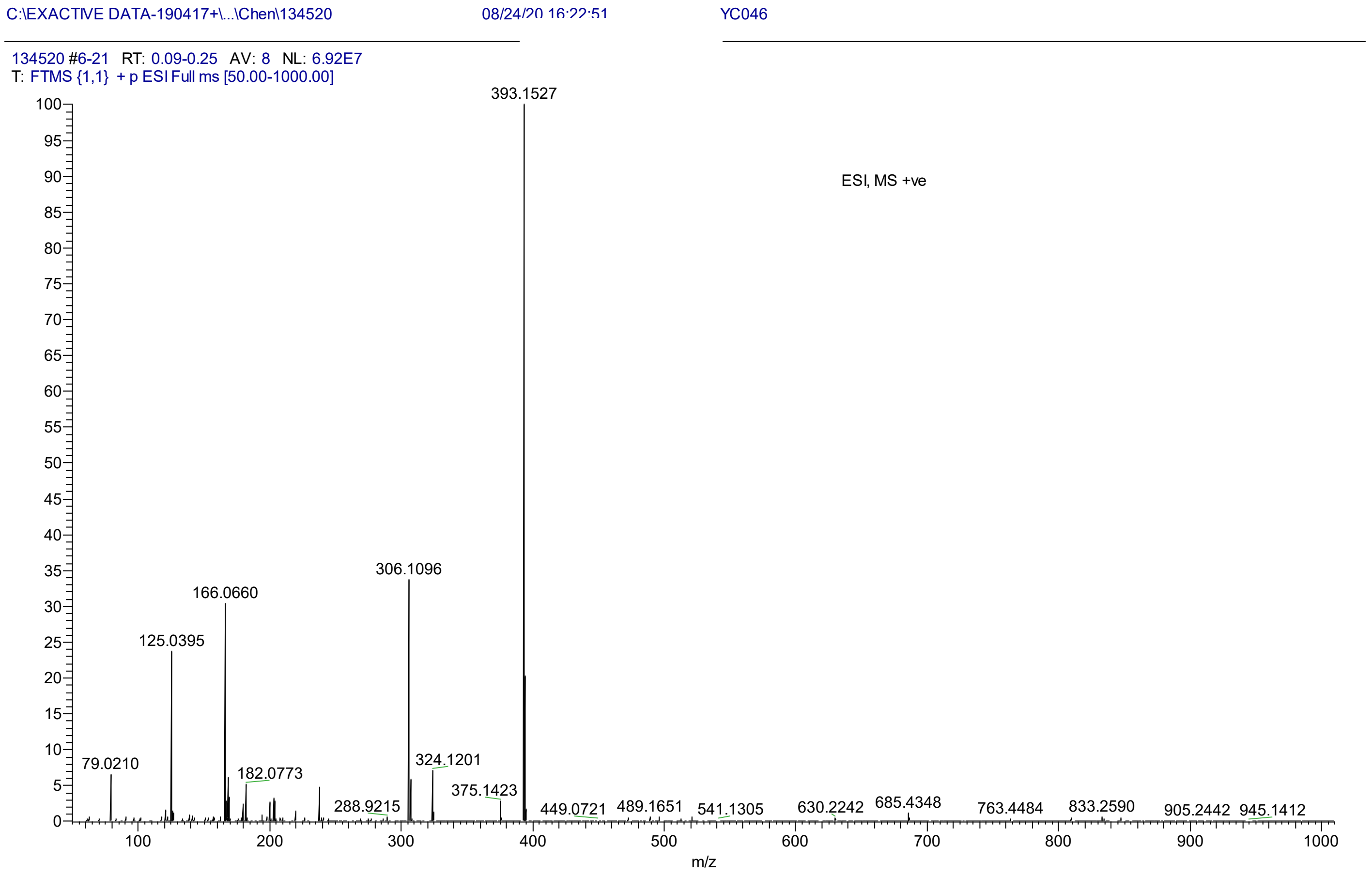

Figure S13-3: HRMS spectrum of Compound **13.**

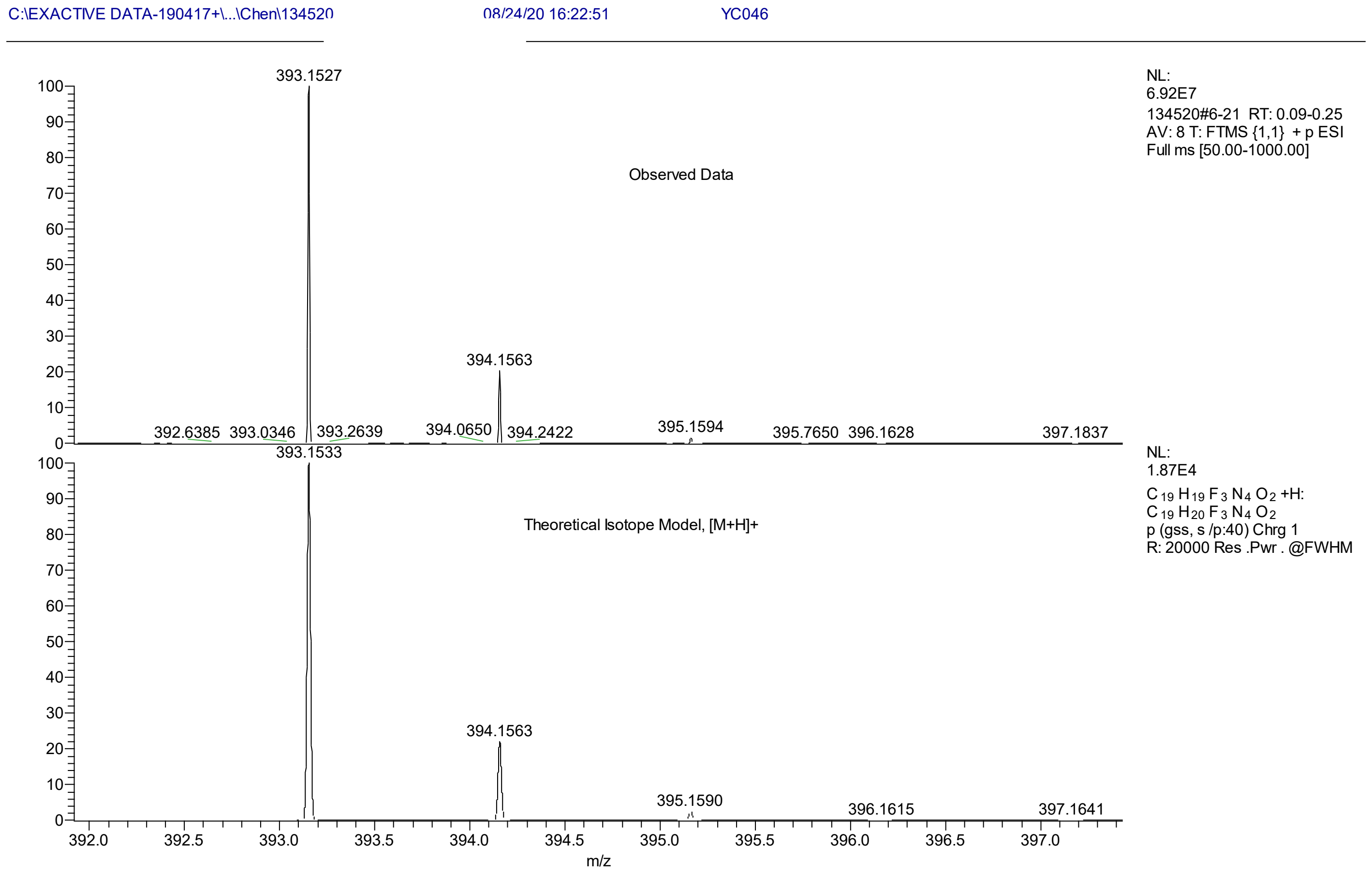

Figure S13-4: HRMS spectrum of Compound **14**

3-chloro-5-(((2-(2,4-difluorophenyl)-2-hydroxy-3-(1H-1,2,4-triazol-1-yl)propyl)(methyl)amino)methyl)phenol (14)

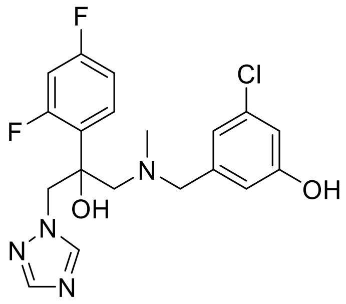

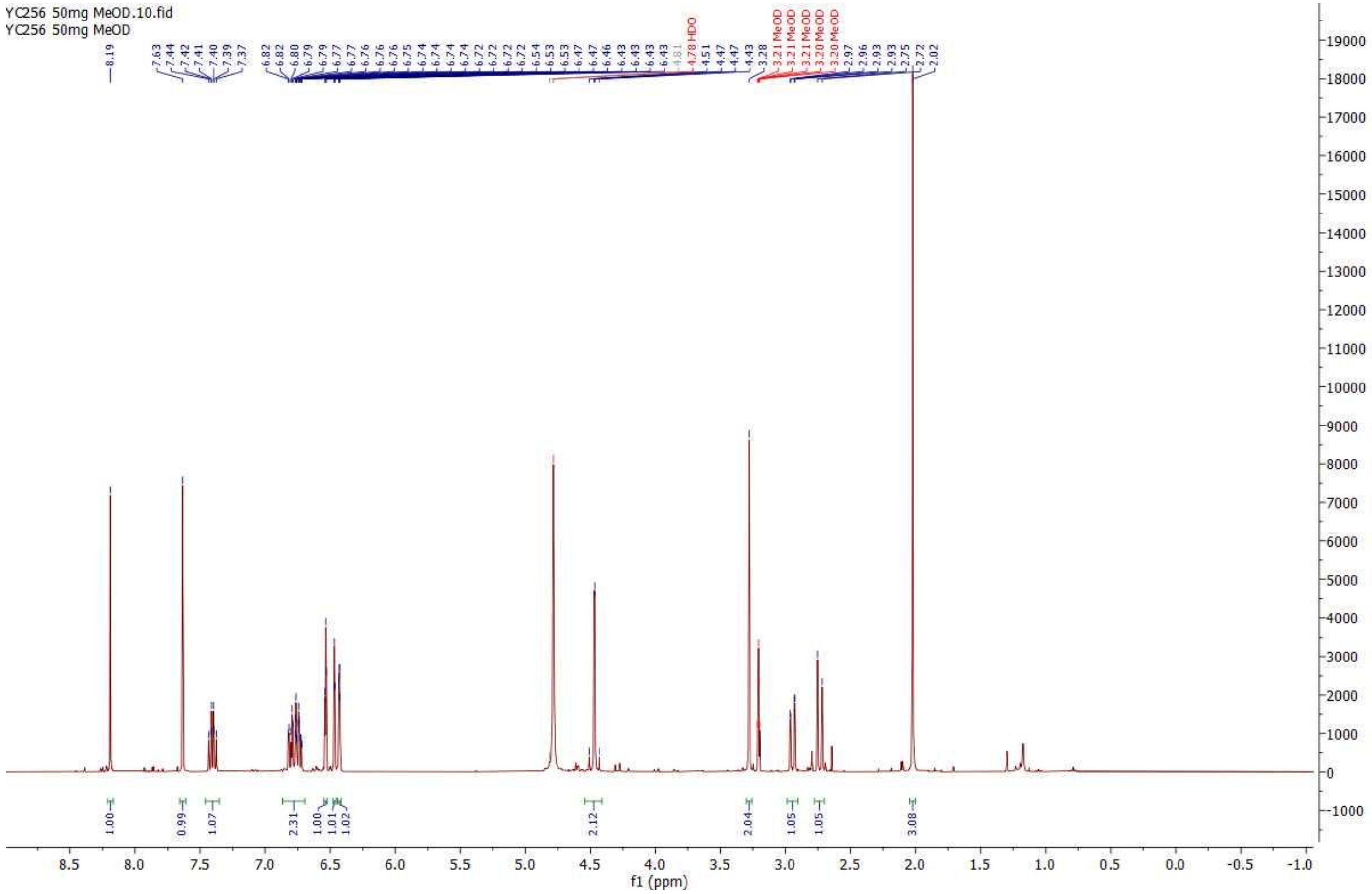

Figure S14-1: Proton ^{1}H NMR spectrum of Compound **14.**

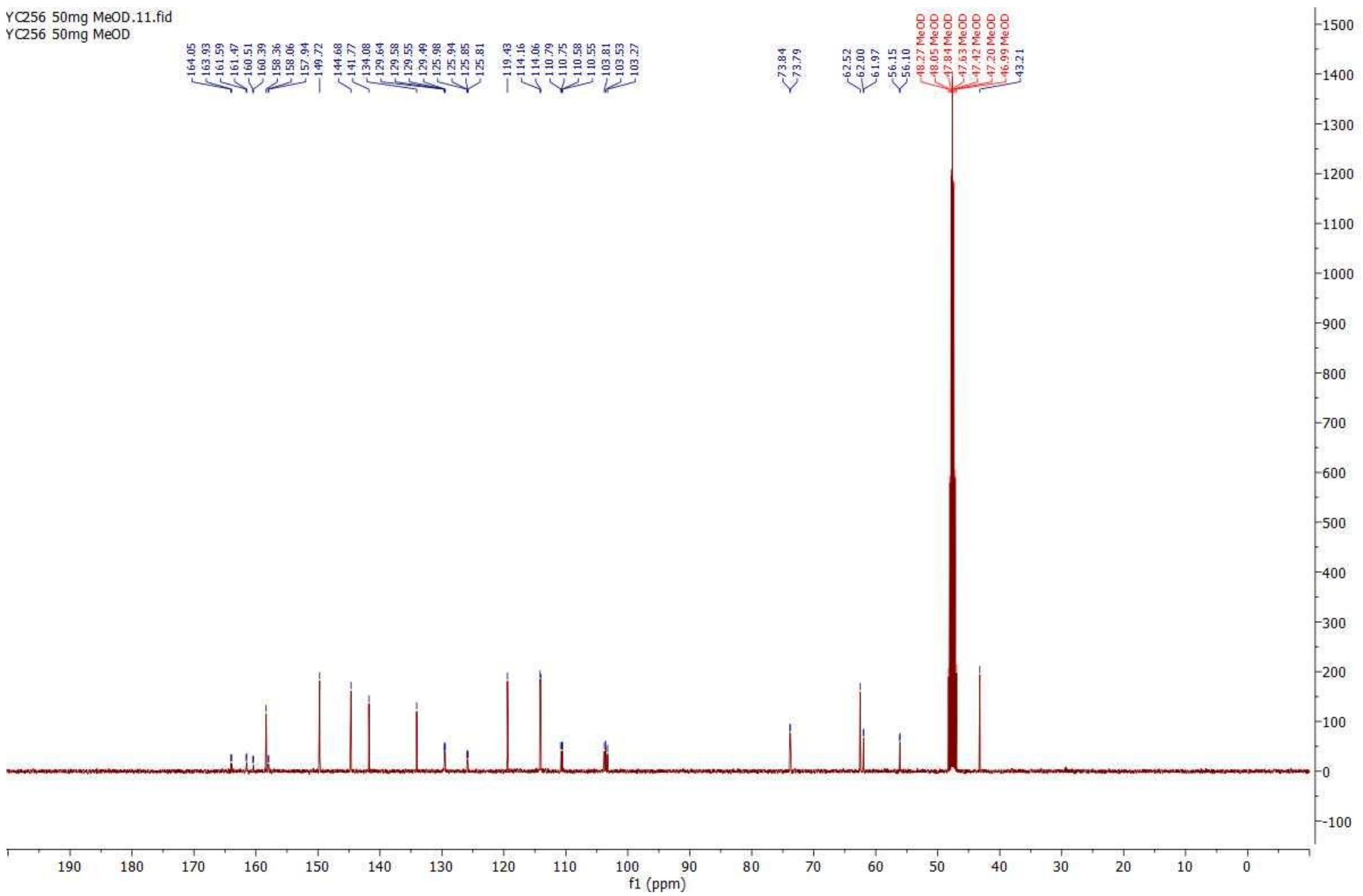

Figure S14-2: Carbon ^{13}C NMR spectrum of Compound **14.**

3-bromo-5-(((2-(2,4-difluorophenyl)-2-hydroxy-3-(1H-1,2,4-triazol-1-yl)propyl)(methyl)amino)methyl)phenol (15)

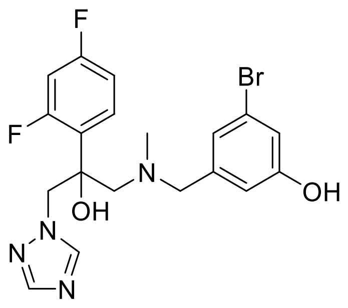

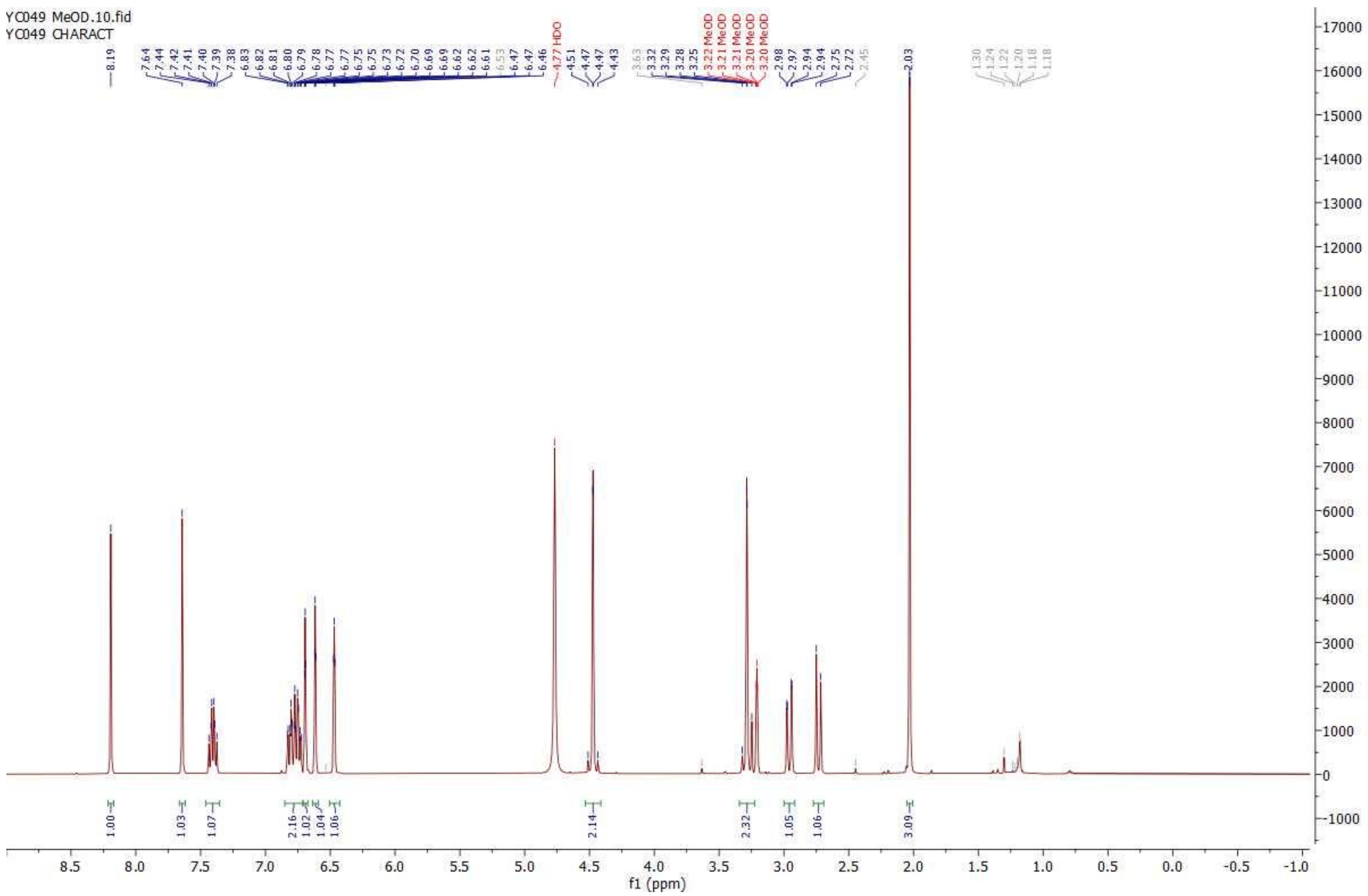

Figure S15-1: Proton ^{1}H NMR spectrum of Compound **15.**

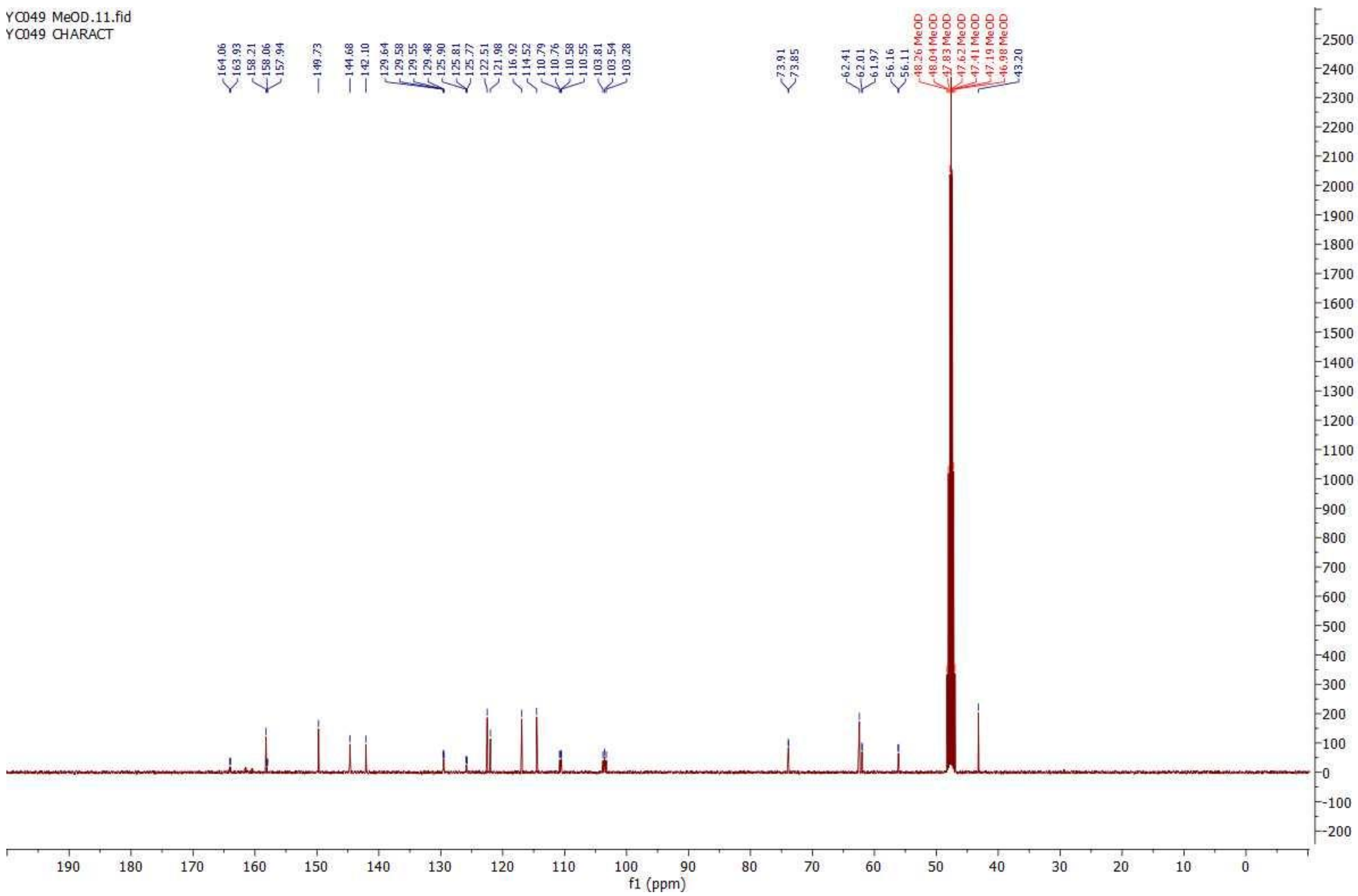

Figure S15-2: Carbon ^{13}C NMR spectrum of Compound **15.**

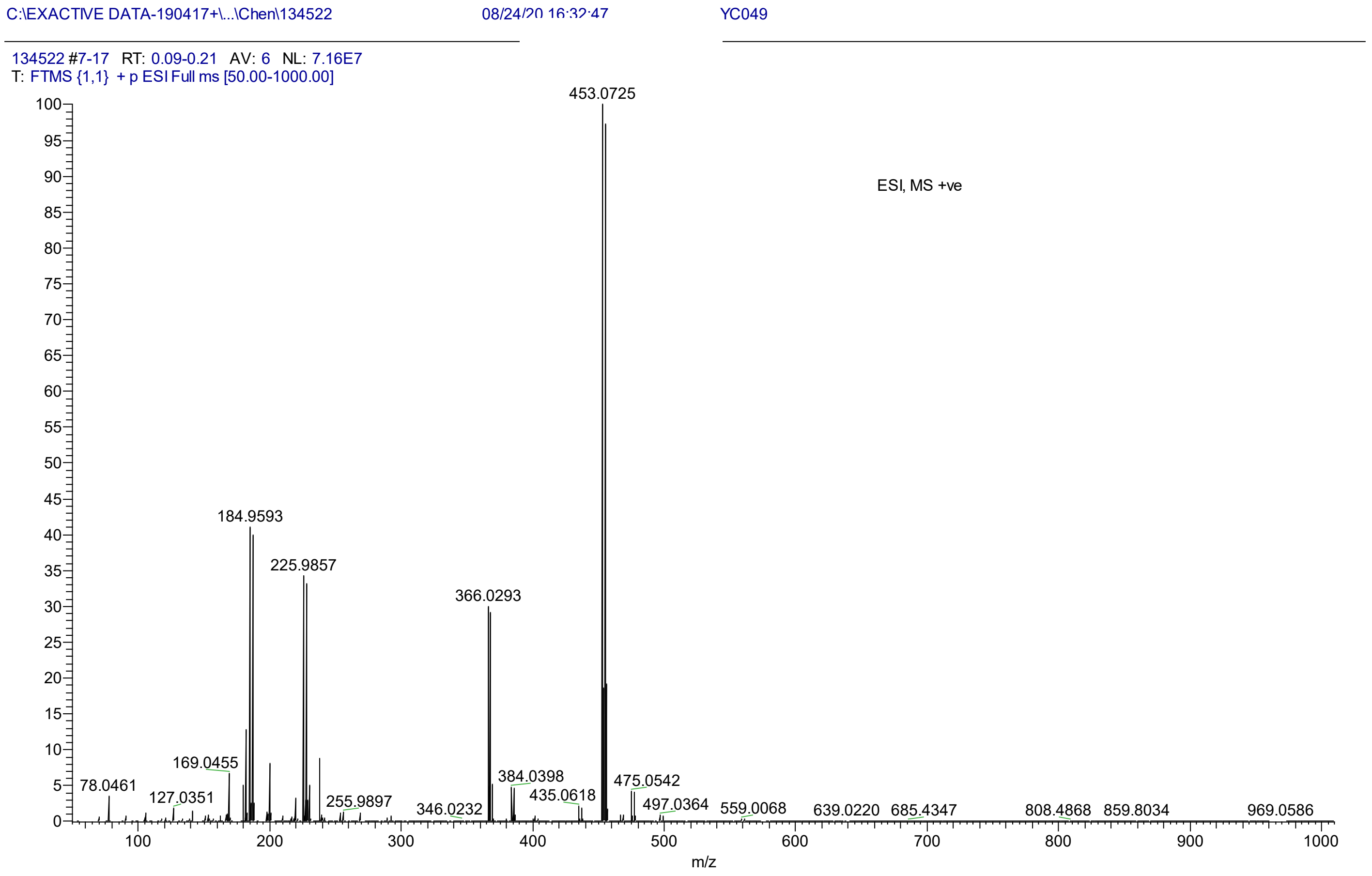

Figure S15-3: HRMS spectrum of Compound **15.**

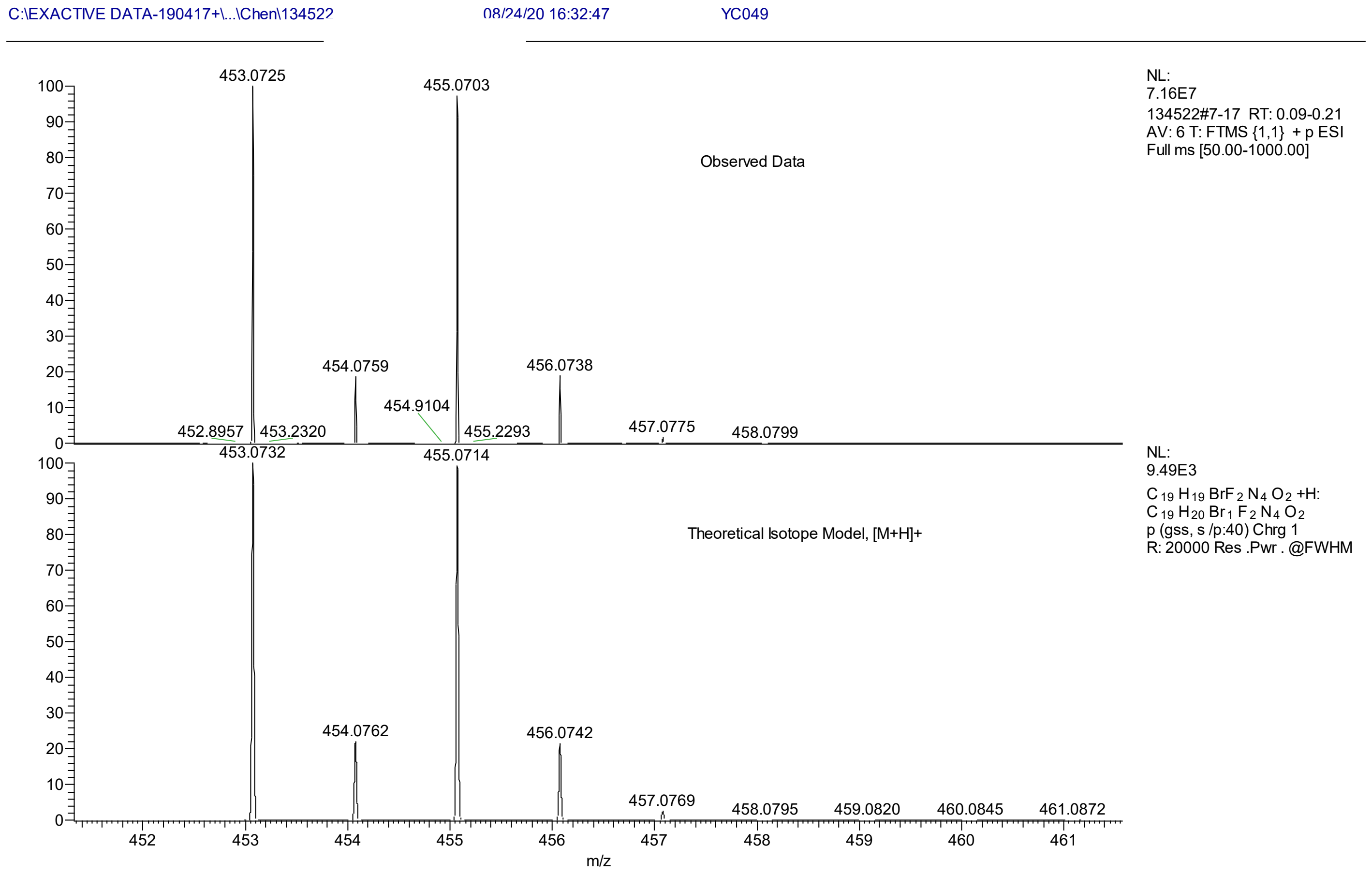

Figure S15-4: HRMS spectrum of Compound **15.**

1-((3,5-difluorobenzyl)(methyl)amino)-2-(2,4-difluorophenyl)-3-(1H-1,2,4-triazol-1-yl)propan-2-ol (16)

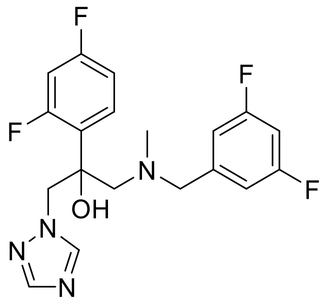

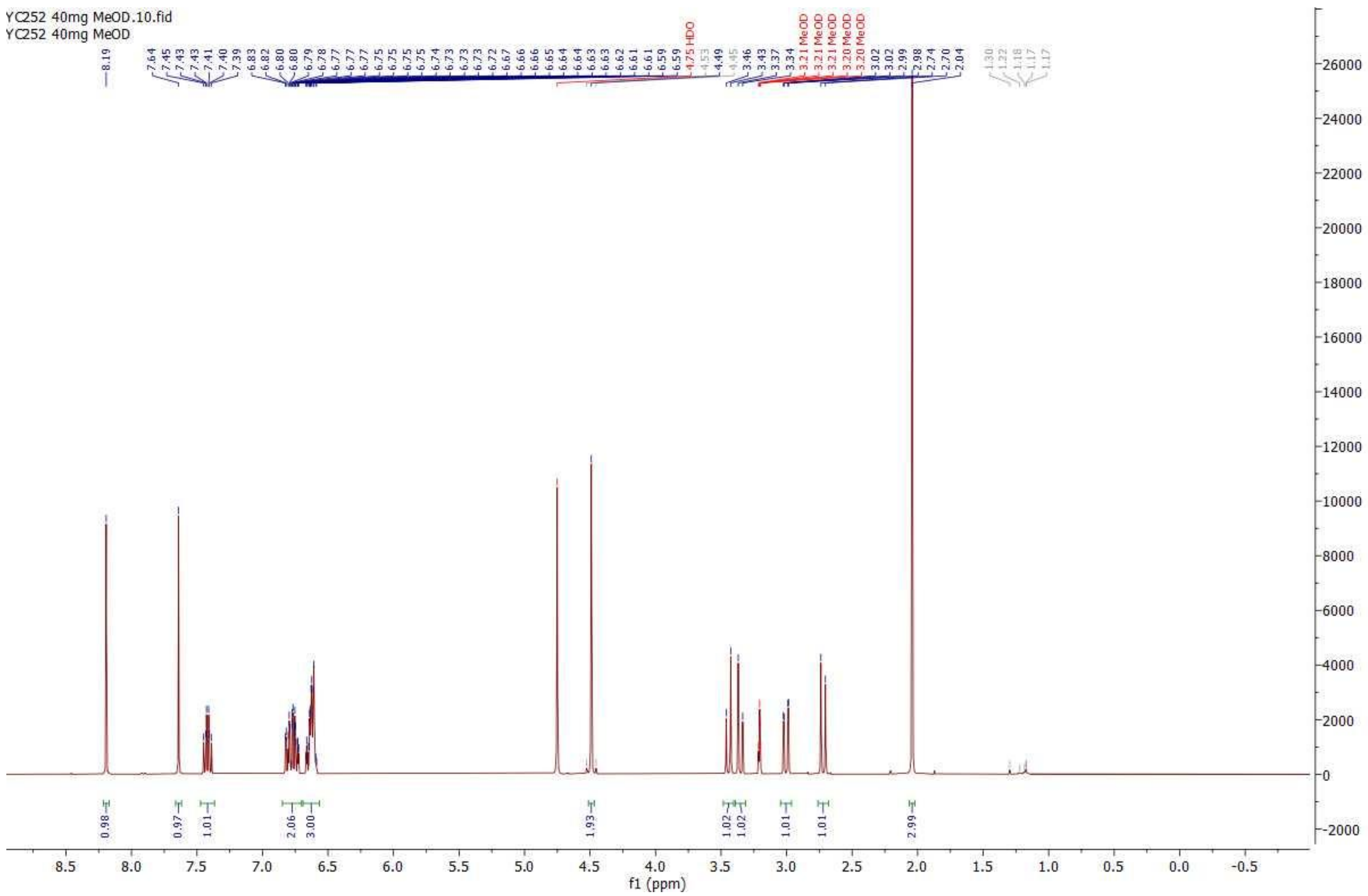

Figure S16-1: Proton ^{1}H NMR spectrum of Compound **16.**

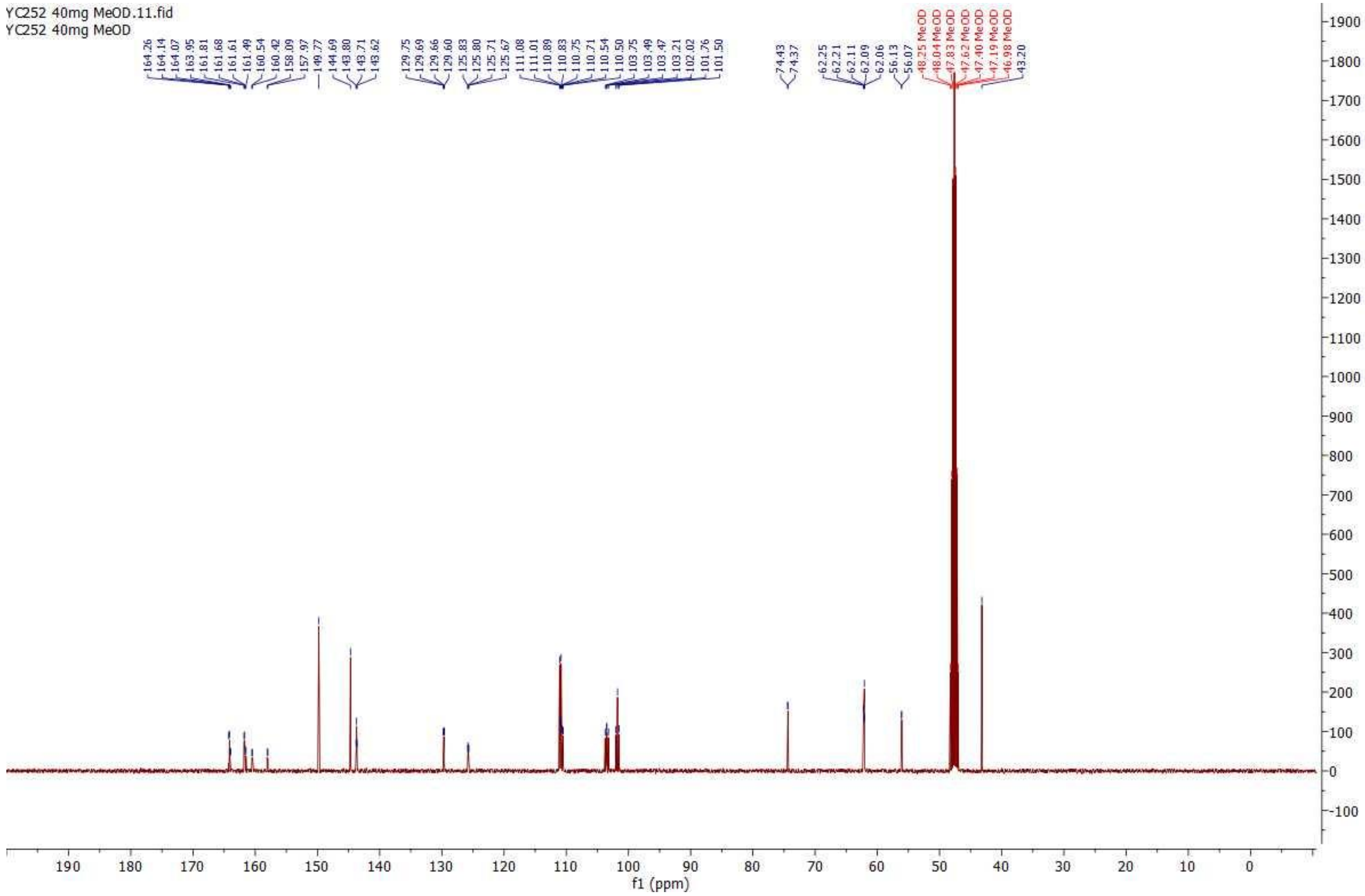

Figure S16-2: Carbon ^{13}C NMR spectrum of Compound **16.**

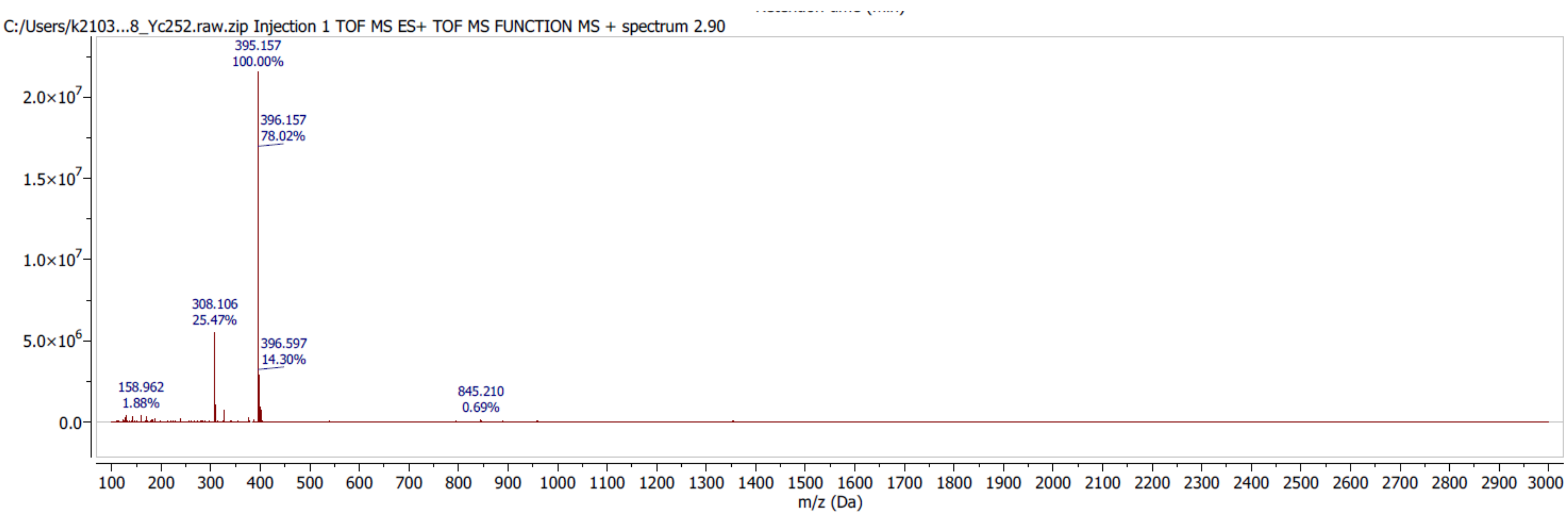

Figure S16-3: HRMS spectrum of Compound **16.**

1-((3-chloro-5-fluorobenzyl)(methyl)amino)-2-(2,4-difluorophenyl)-3-(1H-1,2,4-triazol-1-yl)propan-2-ol (17)

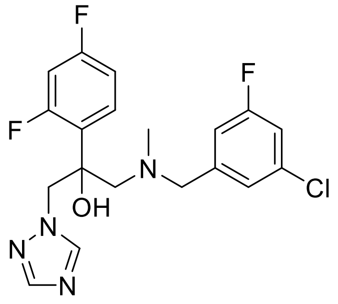

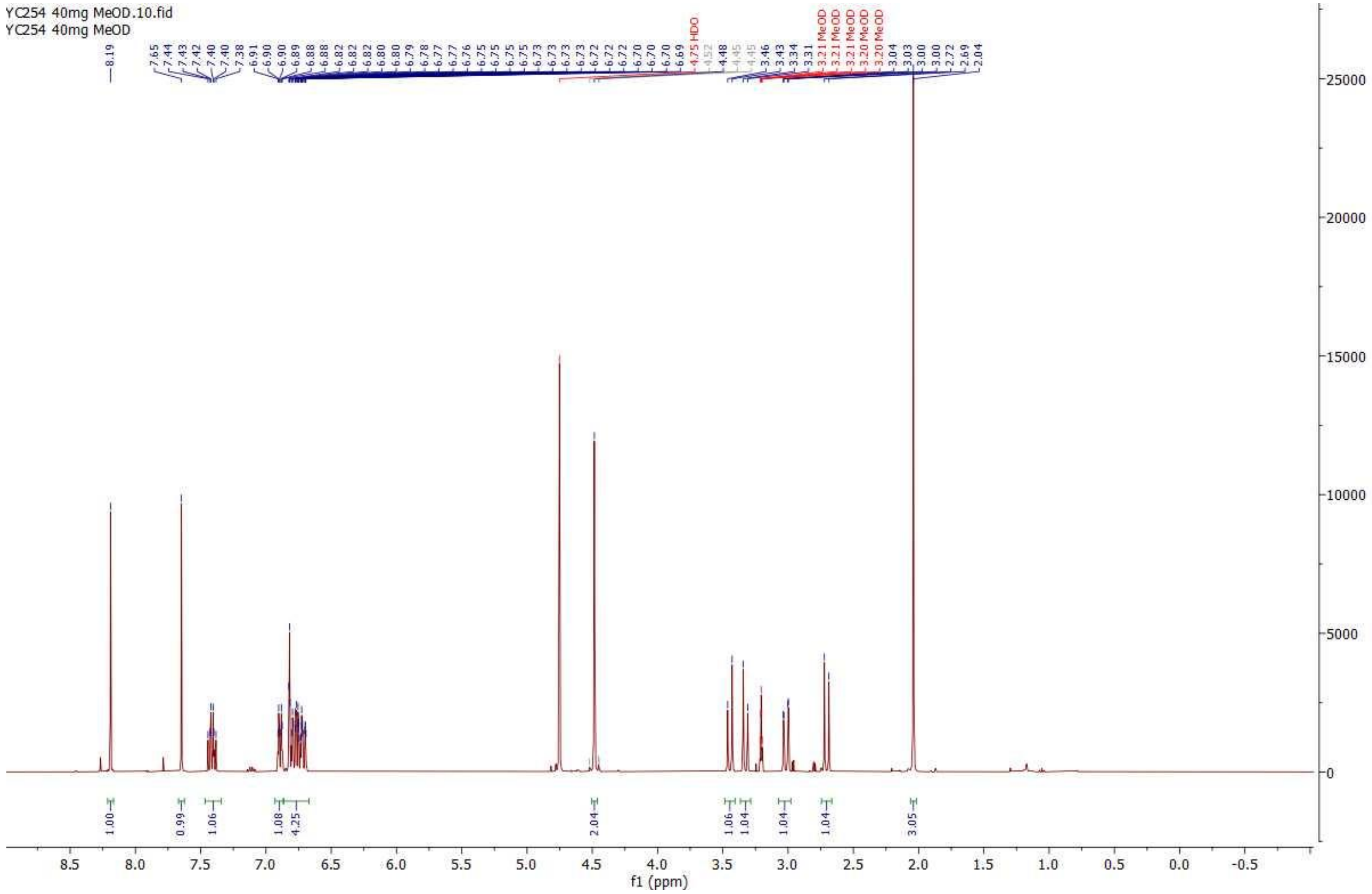

Figure S17-1: Proton ^{1}H NMR spectrum of Compound **17.**

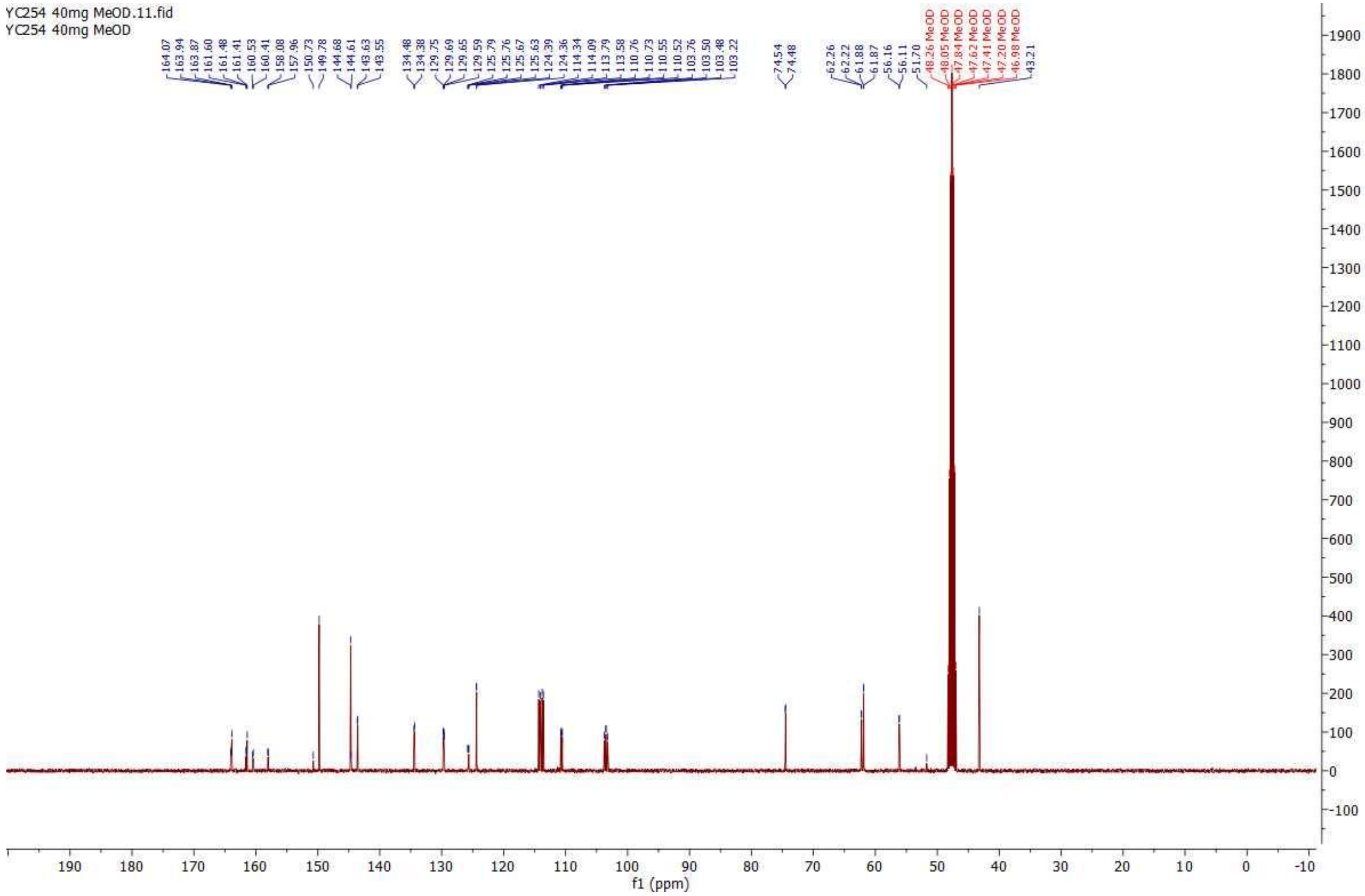

Figure S17-2: Carbon ^{13}C NMR spectrum of Compound **17.**

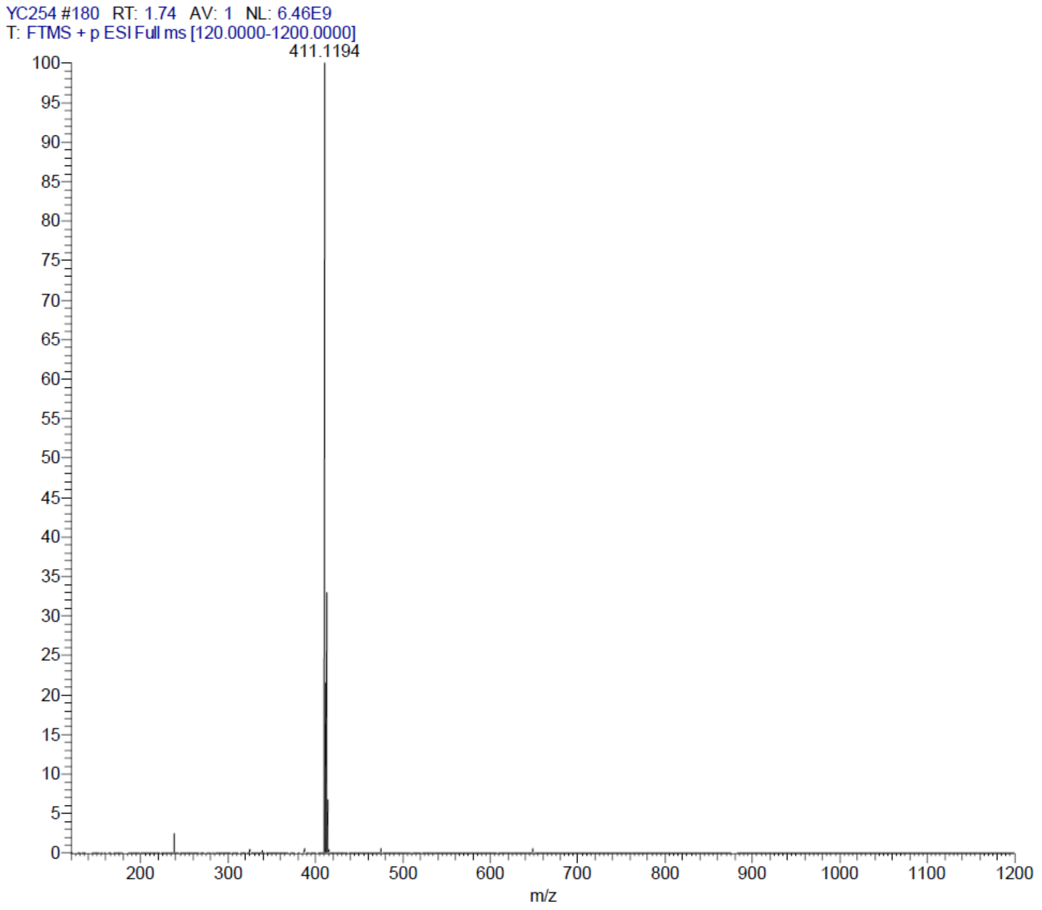

Figure S17-3: HRMS spectrum of Compound **17.**

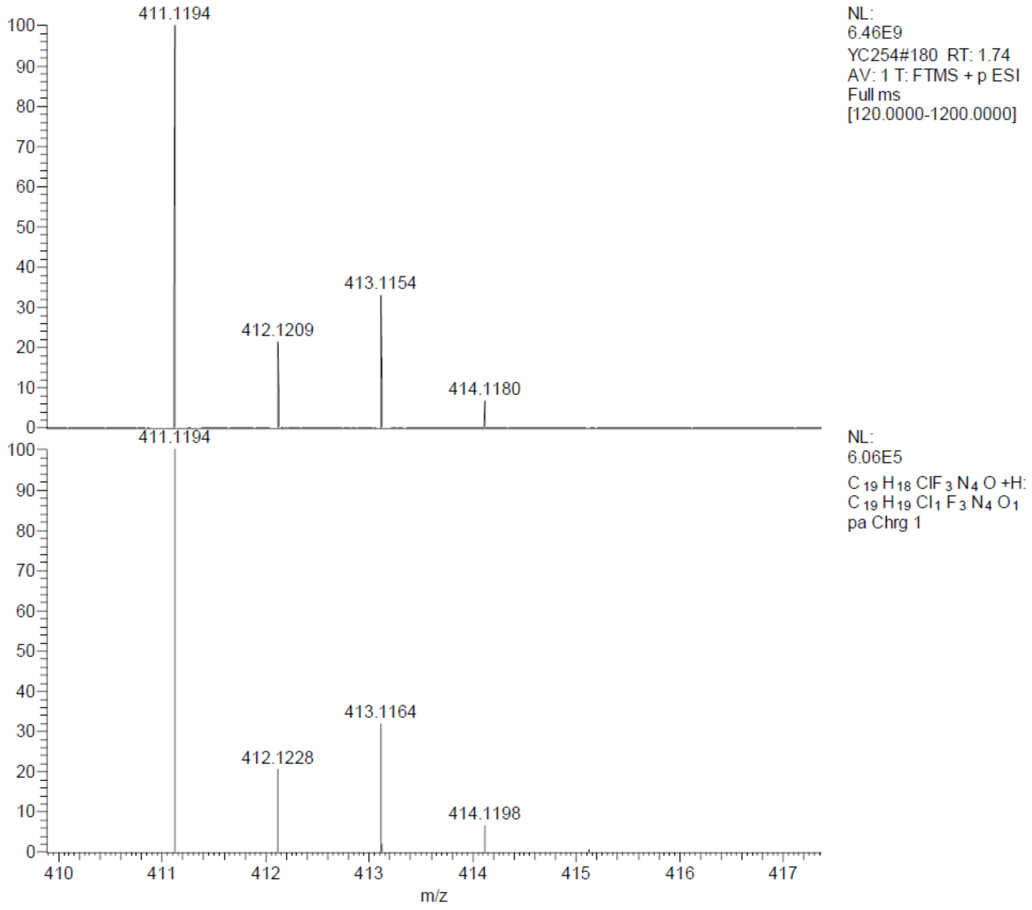

Figure S17-4: HRMS spectrum of Compound **17.**

1-((3-bromo-5-fluorobenzyl)(methyl)amino)-2-(2,4-difluorophenyl)-3-(1H-1,2,4-triazol-1-yl)propan-2-ol (18)

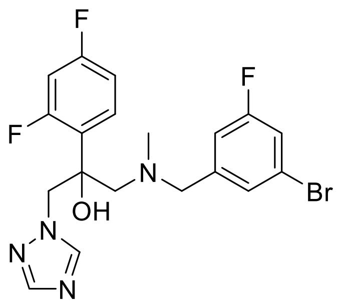

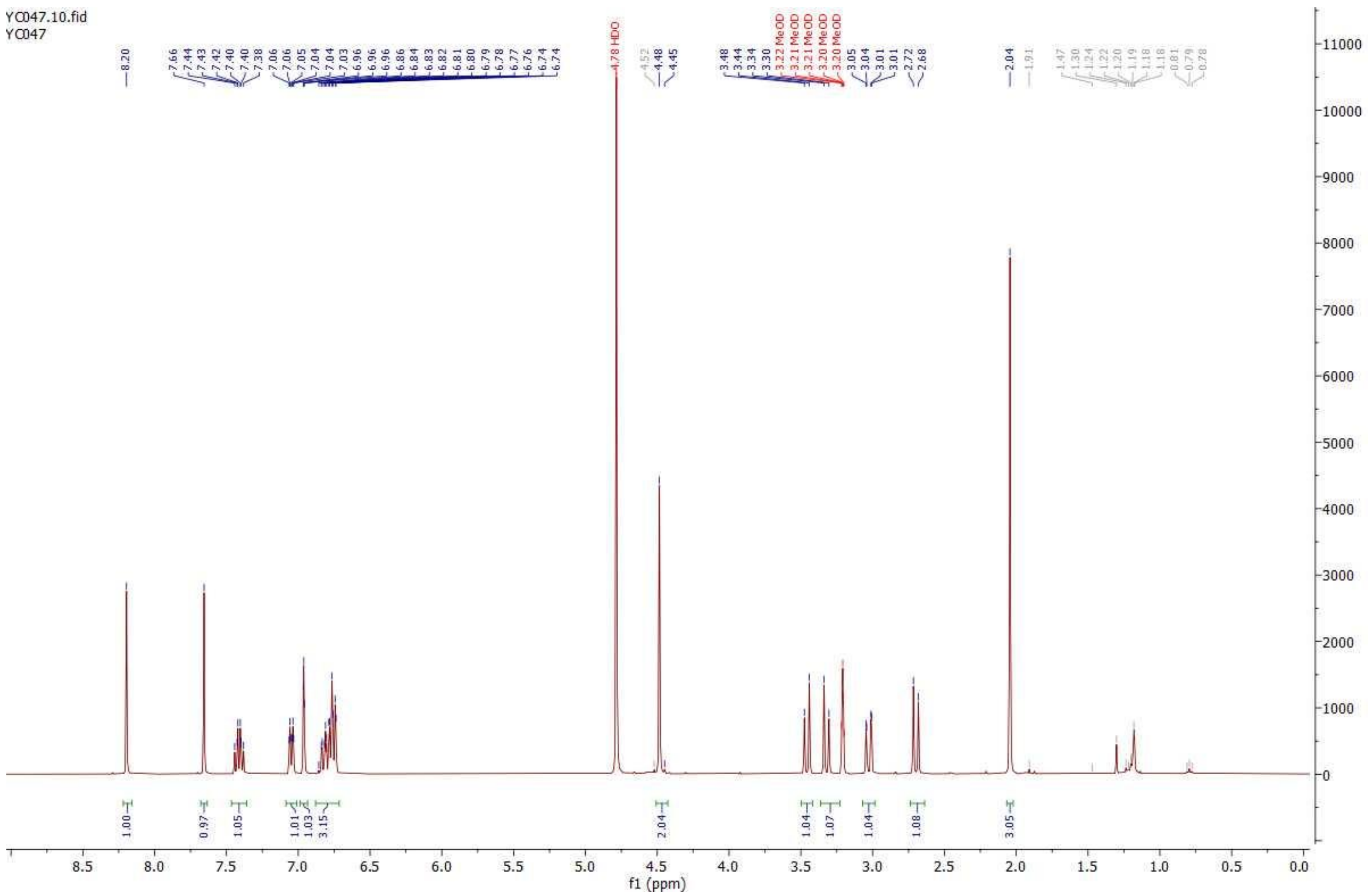

Figure S18-1: Proton ^{1}H NMR spectrum of Compound **18.**

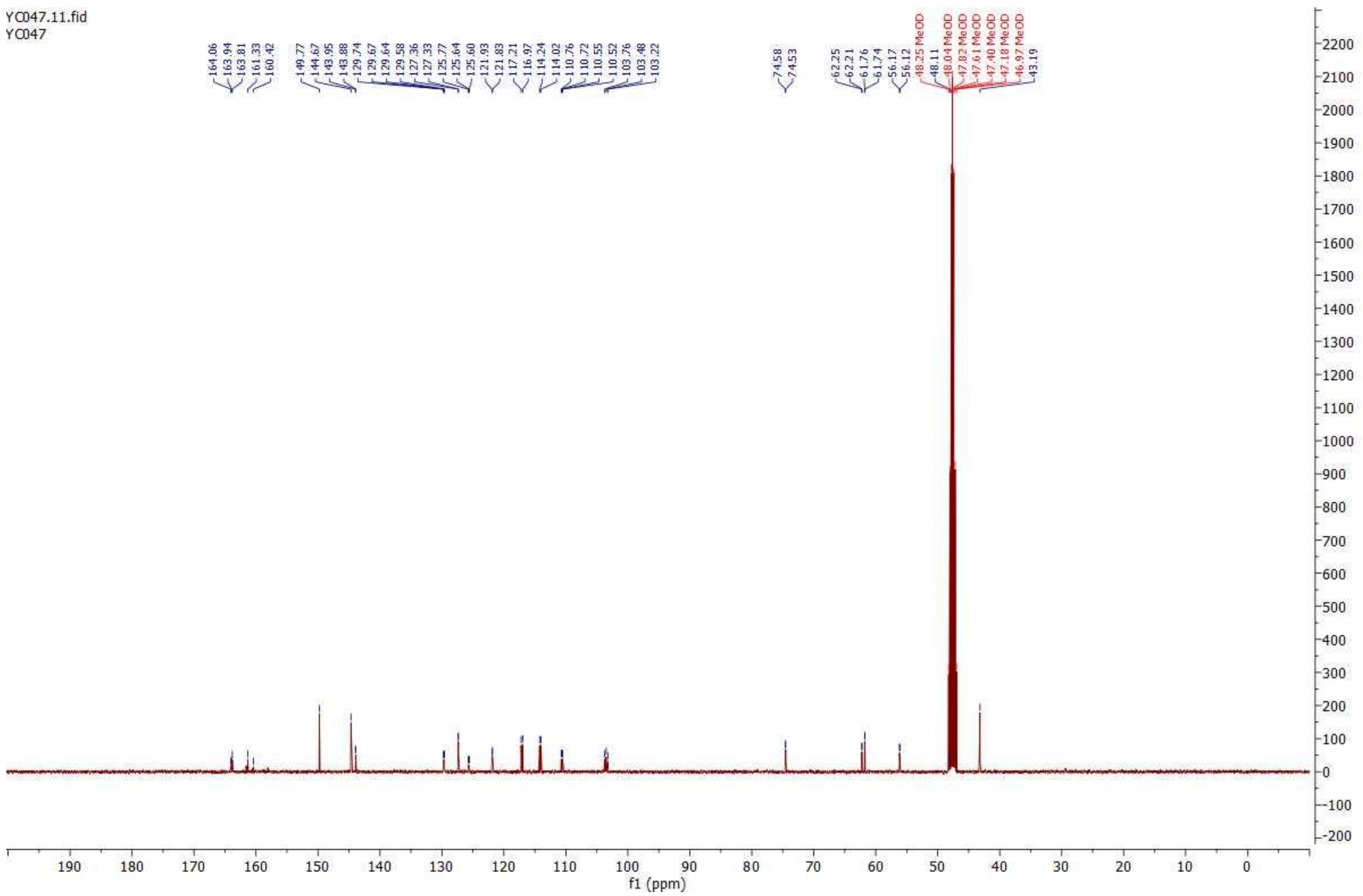

Figure S18-2: Carbon ^{13}C NMR spectrum of Compound **18.**

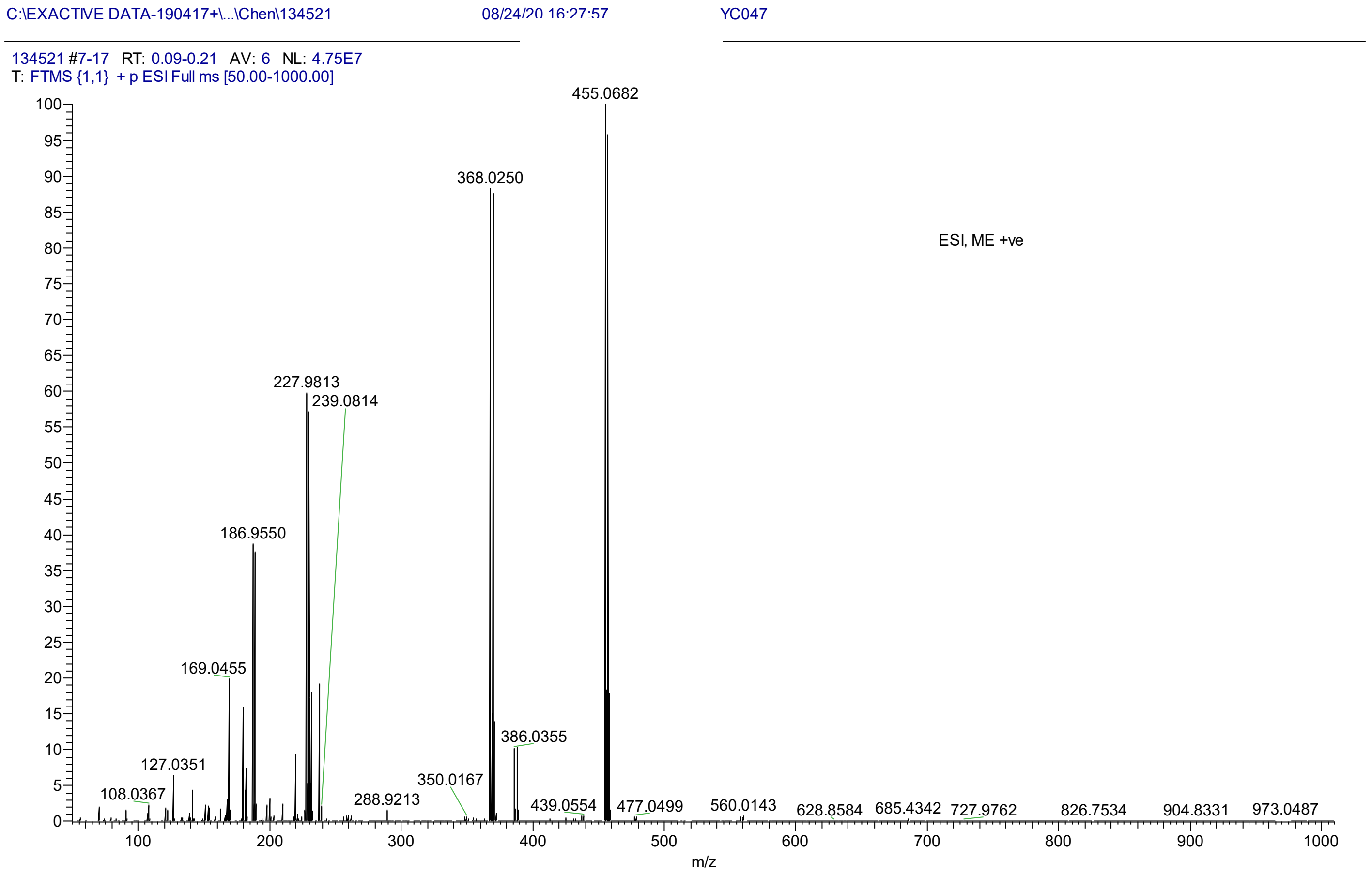

Figure S18-3: HRMS spectrum of Compound **18.**

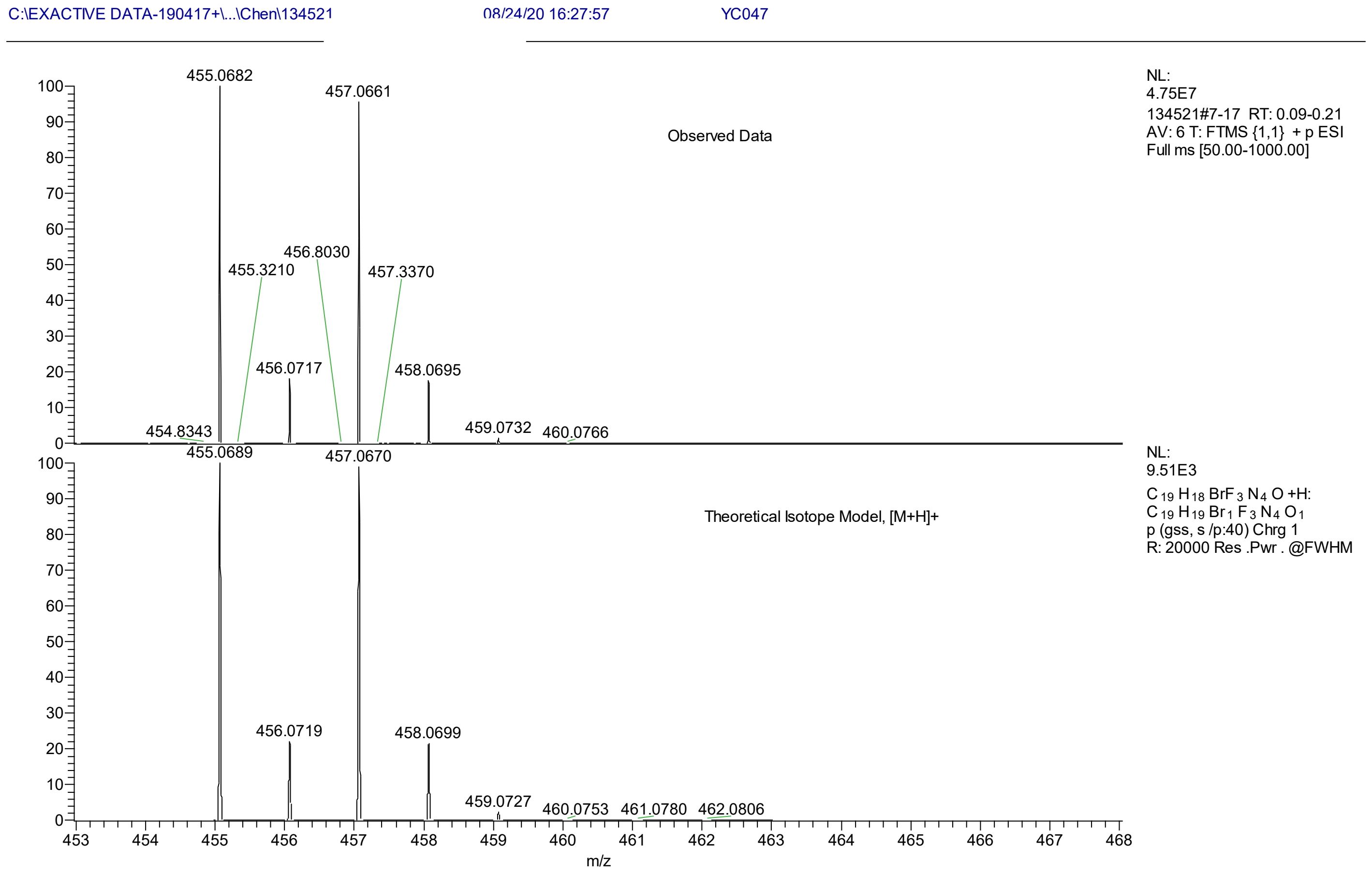

Figure S18-4: HRMS spectrum of Compound **18.**

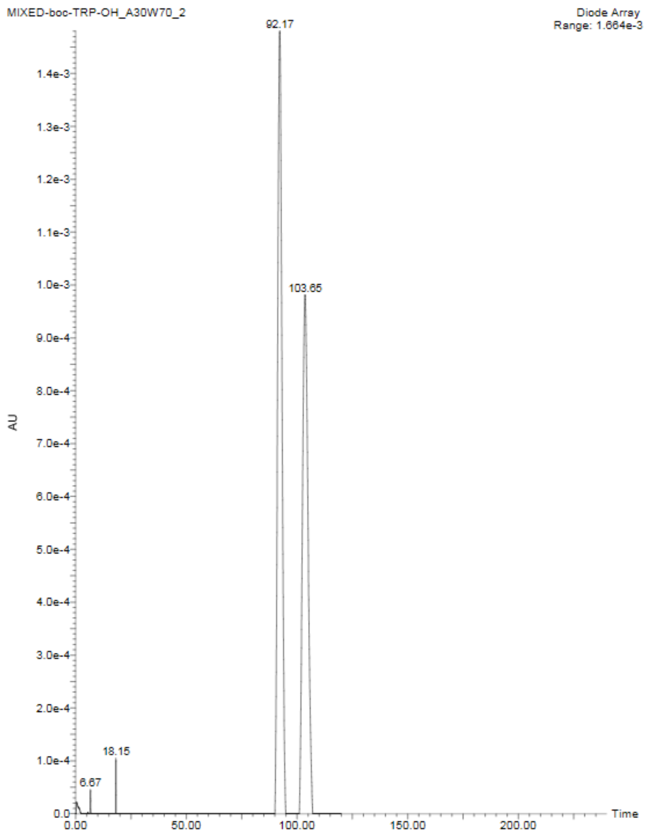

Figure S19-A: Reverse phase (Method Validation) chiral HPLC analysis of R-Boc-Trp-OH and D-Boc-Trp-OH racemic reference mixture showing two peaks at RT 92.17 and 103.65 minutes. HPLC method: MeCN/H2O/formic acid (30/70/0.1 %), isocratic run for 120 mins, flow rate 0.5 mL min^{-1}, monitored at λ = 260 nm.

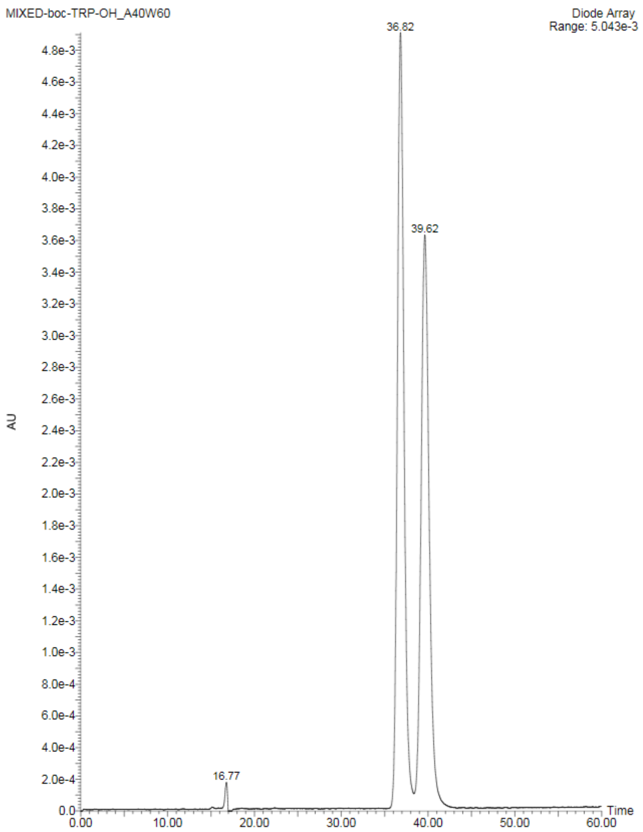

Figure S19-B: Reverse phase (Method Validation) chiral HPLC analysis of R-Boc-Trp-OH and D-Boc-Trp-OH racemic reference mixture showing two peaks at RT 36.82 and 39.62 minutes. HPLC method: MeCN/H2O/formic acid (40/60/0.1 %), isocratic run for 60 mins, flow rate 0.5 mL min^{-1}, monitored at λ = 260 nm.

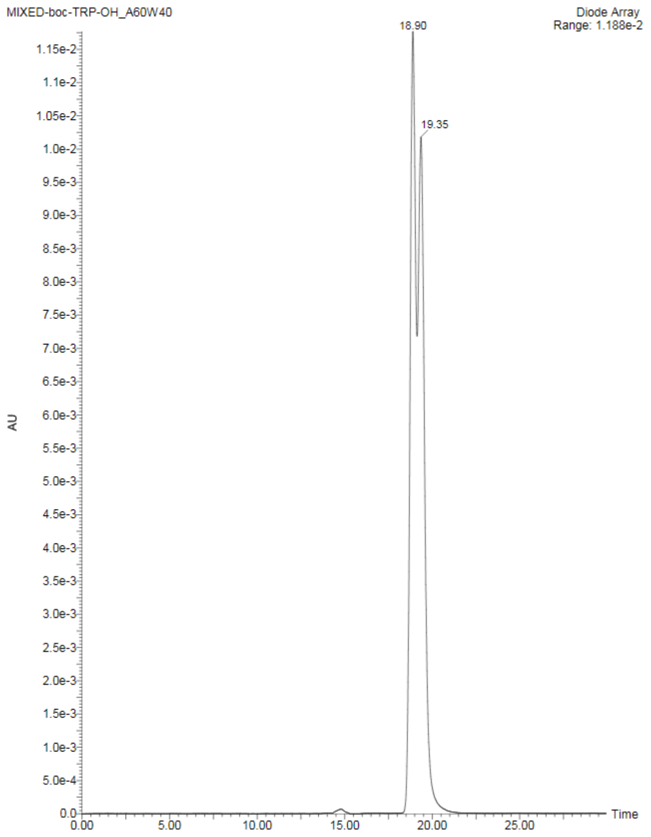

Figure S19-C: Reverse phase (Method Validation) chiral HPLC analysis of R-Boc-Trp-OH and D-Boc-Trp-OH racemic mixture showing two peaks at RT 18.90 and 19.35 minutes. HPLC method: MeCN/H2O/formic acid (60/40/0.1 %), isocratic run for 30 mins, flow rate 0.5 mL min^{-1}, monitored at λ = 260 nm.

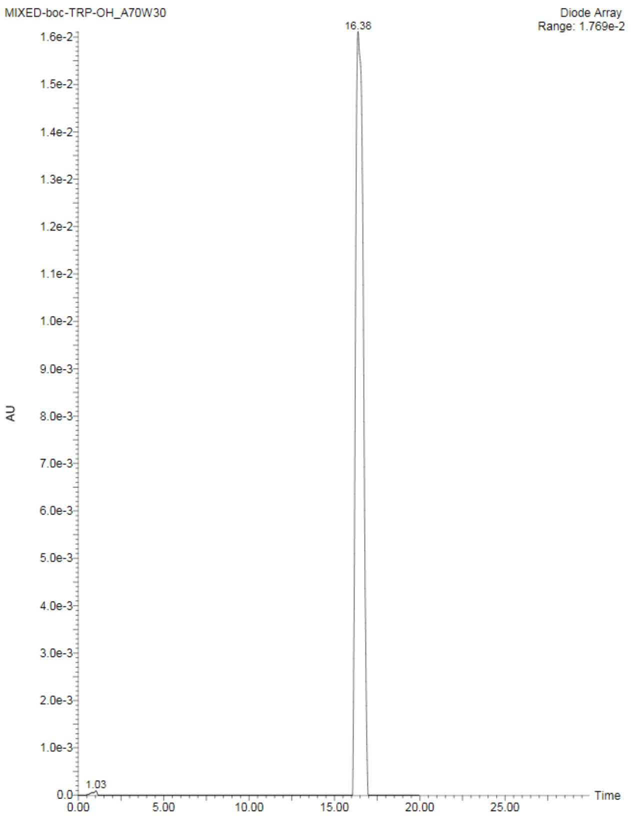

Figure S19-D: Reverse phase (Method validation) chiral HPLC analysis of R-Boc-Trp-OH and D-Boc-Trp-OH racemic mixture showing a single peak at RT 16.38 minutes. HPLC method: MeCN/H2O/formic acid (70/30/0.1 %), isocratic run for 20 mins, flow rate 0.5 mL min^{-1}, monitored at λ = 260 nm.

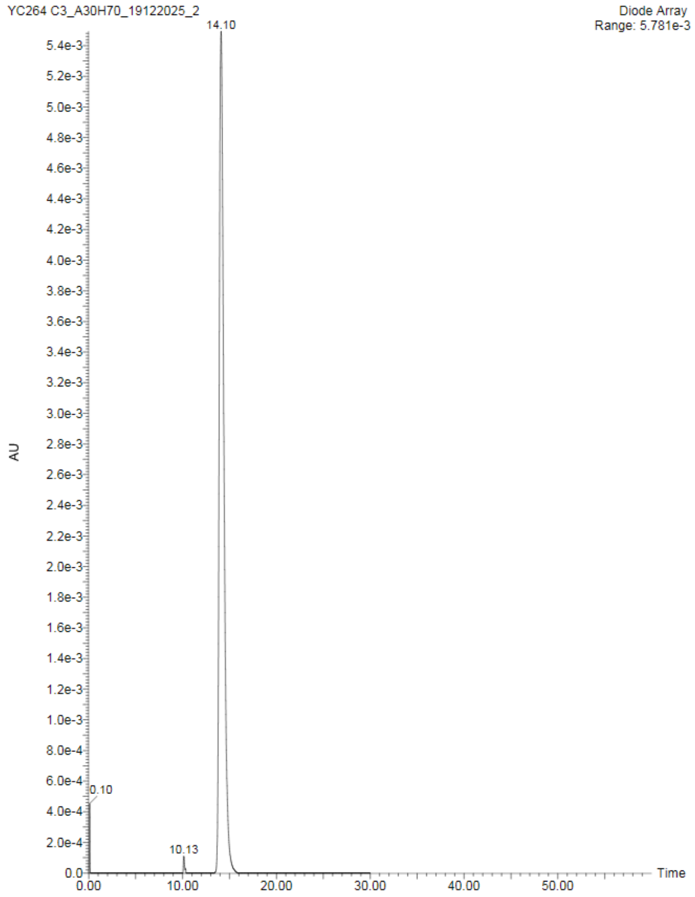

Figure S20-A: Reverse phase chiral HPLC analysis of compound **3** (YC-264) showing a single peak at RT 14.10 minutes. HPLC method: MeCN/H2O/formic acid (30/70/0.1 %), isocratic run for 30 mins, flow rate 0.5 mL min^{-1}, monitored at λ = 260 nm.

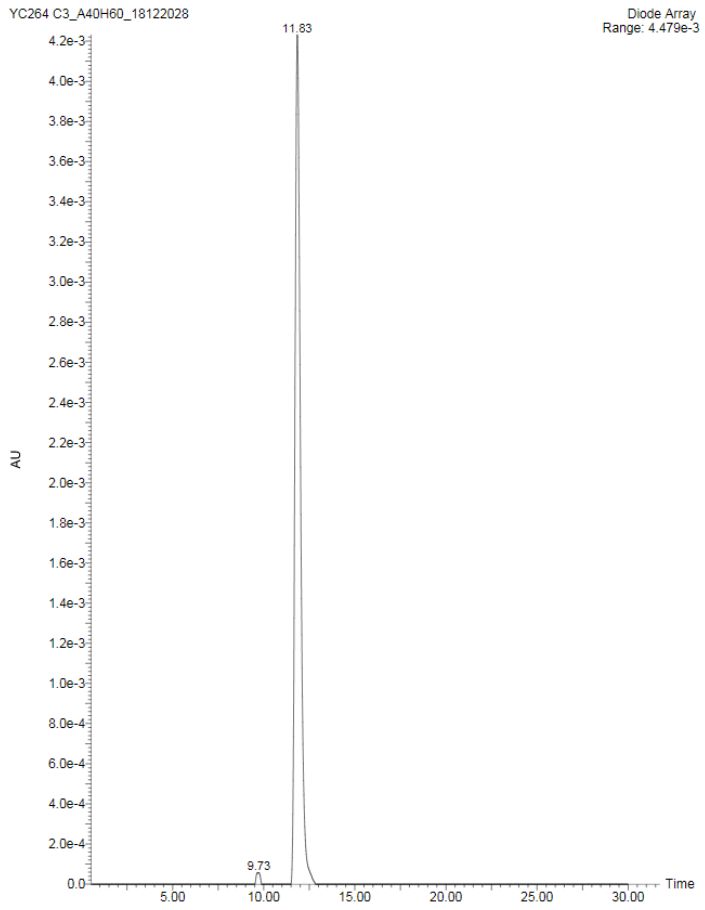

Figure S20-B: Reverse phase chiral HPLC analysis of compound **3** (YC-264) showing a single peak at RT 11.83 minutes. HPLC method: MeCN/H2O/formic acid (40/60/0.1 %), isocratic run for 30 mins, flow rate 0.5 mL min^{-1}, monitored at λ = 260 nm.

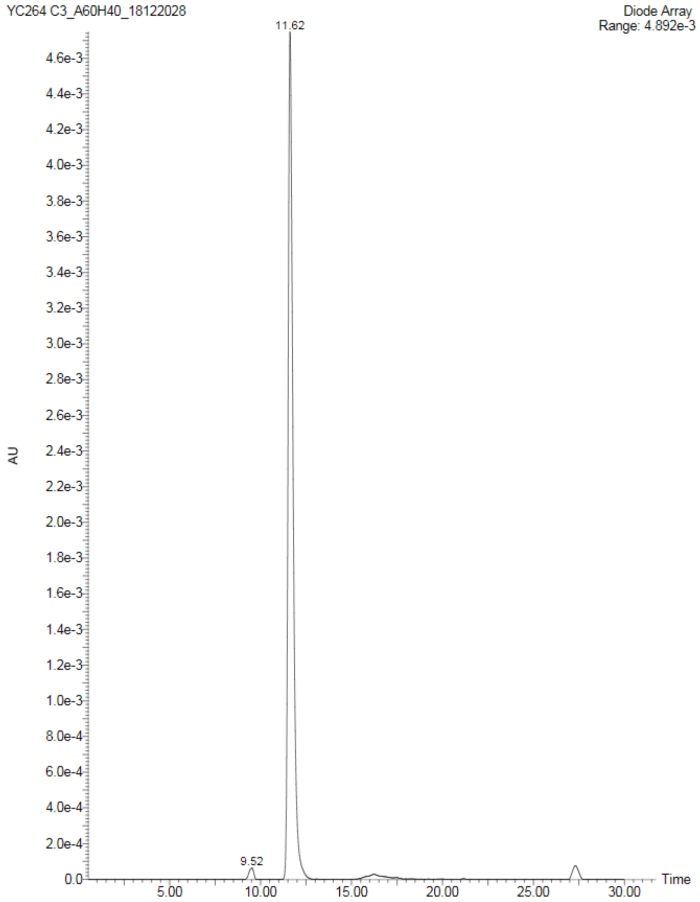

Figure S20-C. Reverse phase chiral HPLC analysis of compound **3** (YC-264) showing a single peak at RT 11.62 minutes. HPLC method: MeCN/H2O/formic acid (60/40/0.1 %), isocratic run for 30 mins, flow rate 0.5 mL min^{-1}, monitored at λ = 260 nm.

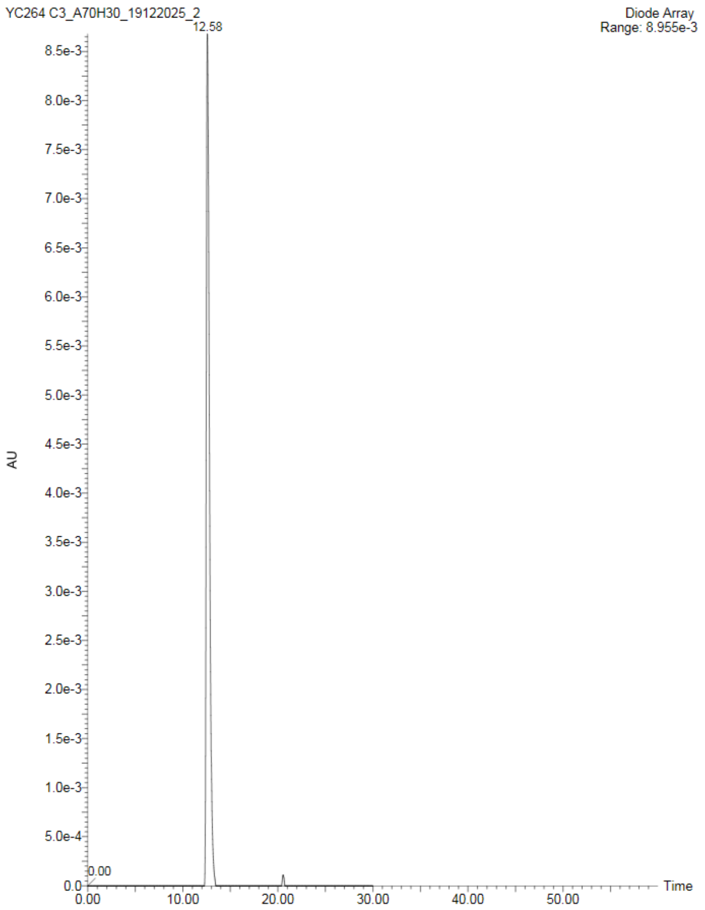

Figure S20-D: Reverse phase chiral HPLC analysis of compound **3** (YC-264) showing a single peak at RT 12.58 minutes. HPLC method: MeCN/H2O/formic acid (70/30/0.1 %), isocratic run for 30 mins, flow rate 0.5 mL min^{-1}, monitored at λ = 260 nm.

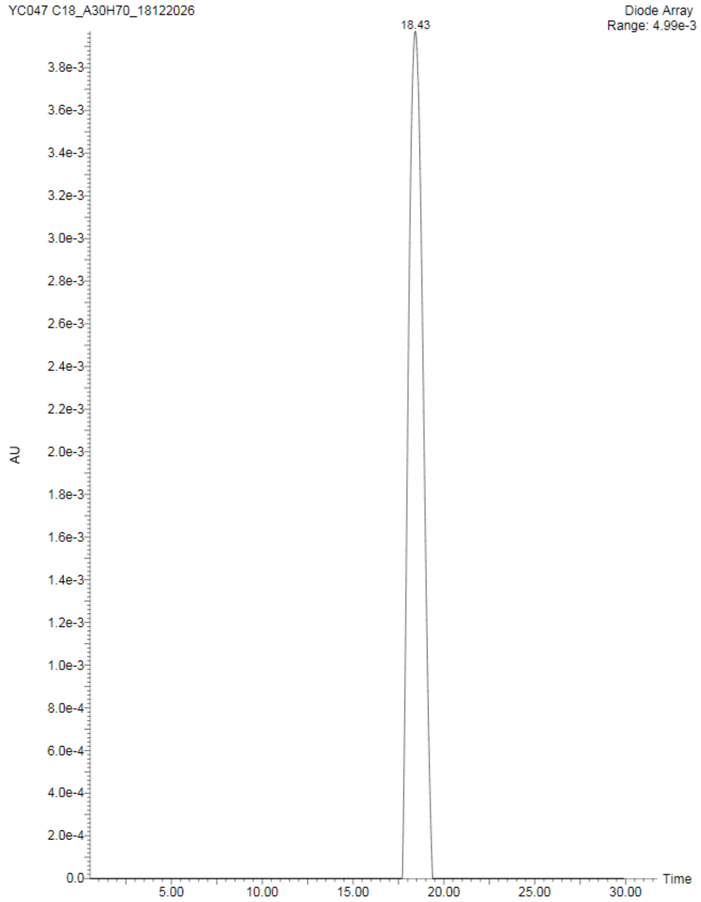

Figure S21-A: Reverse phase chiral HPLC analysis of compound **18** (YC-047) showing a single peak at RT 18.43 minutes. HPLC method: MeCN/H2O/formic acid (30/70/0.1 %), isocratic run for 30 mins, flow rate 0.5 mL min^{-1}, monitored at λ = 260 nm.

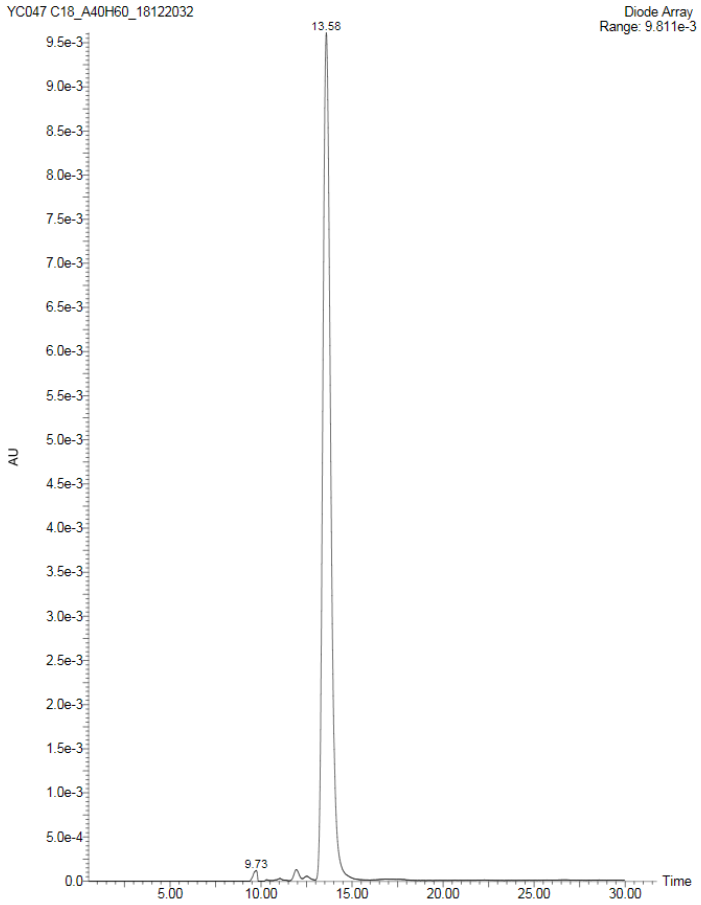

Figure S21-B: Reverse phase chiral HPLC analysis of compound **18** (YC-047) showing a single peak at RT 13.58 minutes. HPLC method: MeCN/H2O/formic acid (40/60/0.1 %), isocratic run for 30 mins, flow rate 0.5 mL min^{-1}, monitored at λ = 260 nm.

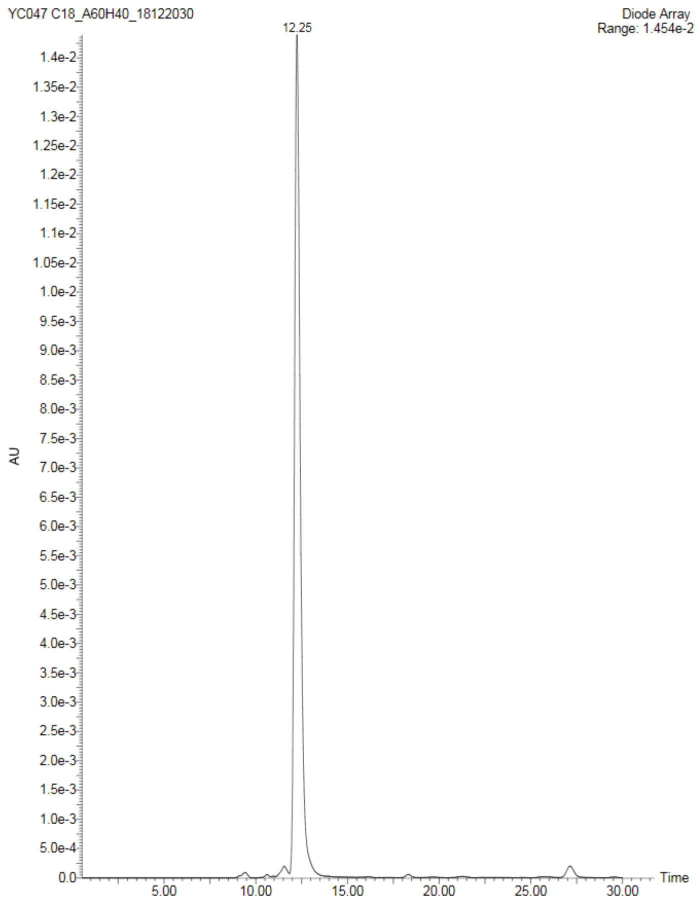

Figure S21-C: Reverse phase chiral HPLC analysis of compound **18** (YC-047) showing a single peak at RT 12.25 minutes. HPLC method: MeCN/H2O/formic acid (60/40/0.1 %), isocratic run for 30 mins, flow rate 0.5 mL min^{-1}, monitored at λ = 260 nm.

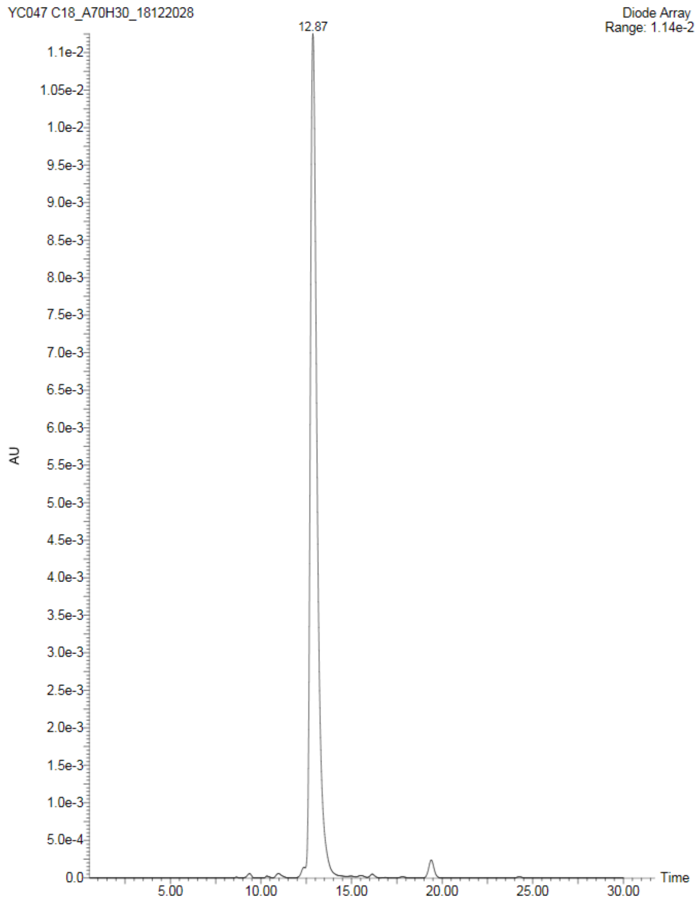

Figure S21-D: Reverse phase chiral HPLC analysis of compound **18** (YC-047) showing a single peak at RT 12.87 minutes. HPLC method: MeCN/H2O/formic acid (70/30/0.1 %), isocratic run for 30 mins, flow rate 0.5 mL min^{-1}, monitored at λ = 260 nm.

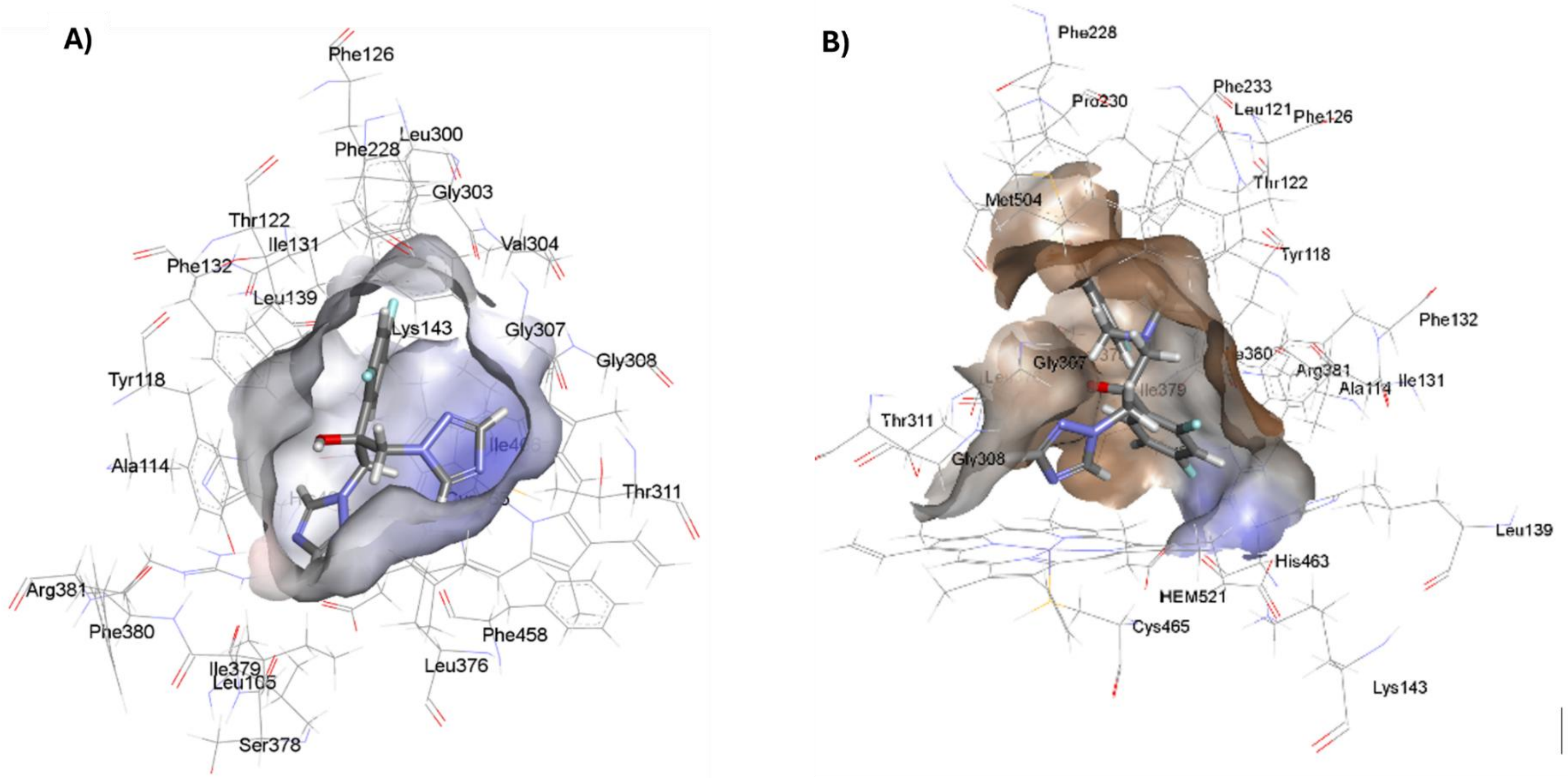

Figure S22: Differences in 3D binding interactions observed for fluconazole (A), and compound **18** (B) with lanosterol 14α-demethylase (LDM) enzyme in *C. auris*. (PDB ID 5TZ1 was used as a template for homology modelling).

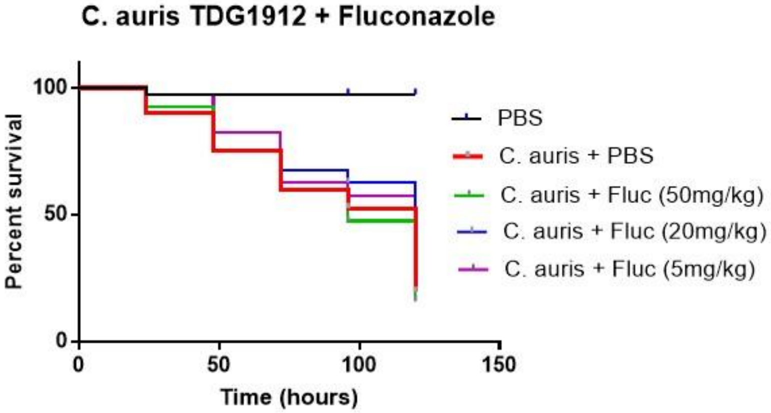

Figure S23. Efficacy of fluconazole against *C. auris* TDG1912 infection in *G. mellonella* model**.**
